# Enhancing Photoredox Catalysis in Aqueous Environments: Ruthenium Aqua Complex Derivatization of Graphene Oxide and Graphite Rods for Efficient Visible-Light-Driven Hybrid Catalysts

**DOI:** 10.1021/acsami.3c13156

**Published:** 2023-12-19

**Authors:** Syrine Affès, Akrivi-Maria Stamatelou, Xavier Fontrodona, Ahlem Kabadou, Clara Viñas, Francesc Teixidor, Isabel Romero

**Affiliations:** † Departament de Química and Serveis Tècnics de Recerca, Universitat de Girona, C/M. Aurèlia Campmany, 69, Girona E-17003, Spain; ‡ Laboratoire des Sciences des Matériaux et d’Environnement, Faculté des Sciences, Université de Sfax, Sfax 3000, Tunisie; § Institut de Ciencia de Materials de Barcelona, ICMAB-CSIC, Campus UAB, Bellaterra E-08193, Spain

**Keywords:** ruthenium, photoredox oxidation, graphene oxide, heterogeneous catalysis, graphite, aqueous medium

## Abstract

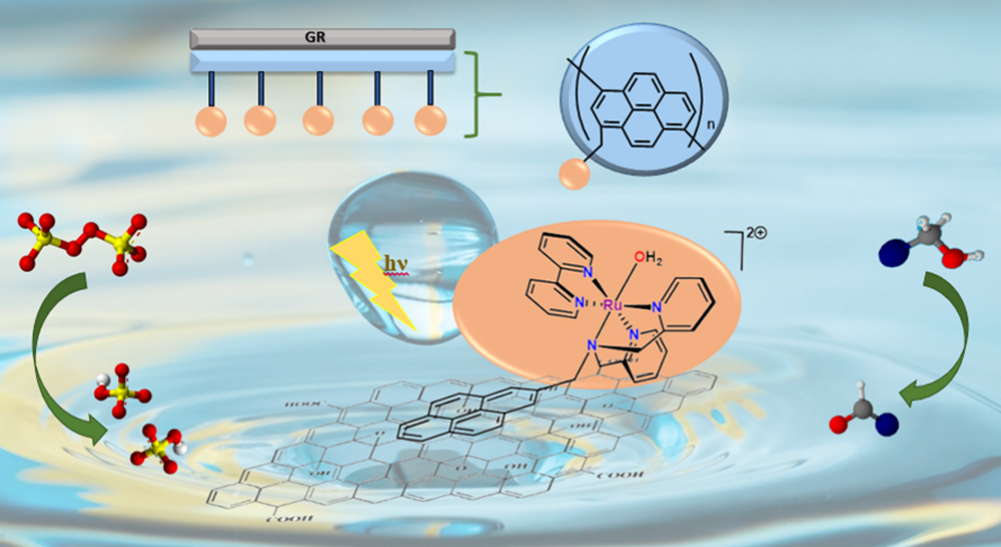

A ruthenium aqua photoredox catalyst has been successfully heterogeneneized on graphene oxide (GO@trans-fac-**3**) and graphite rods (GR@trans-fac-**3**) for the first time and have proven to be sustainable and easily reusable systems for the photooxidation of alcohols in water, in mild and green conditions. We report here the synthesis and total characterization of two Ru(II)-polypyridyl complexes, the chlorido trans-fac-[RuCl(bpea-pyrene)(bpy)](PF_6_) (trans-fac-**2**) and the aqua trans-fac-[Ru(bpea-pyrene)(bpy)OH_2_](PF_6_)_2_ (trans-fac-**3**), both containing the *N*-tridentate, 1-[bis(pyridine-2-ylmethyl)amino]methylpyrene (bpea-pyrene), and 2,2′-bipyridine (bpy) ligands. In both complexes, only a single isomer, the trans-fac, has been detected in solution and in the solid state. The aqua complex trans-fac-**3** displays bielectronic redox processes in water, assigned to the Ru(IV/II) couple. The trans-fac-**3** complex has been heterogenized on different types of supports, (i) on graphene oxide (GO) through π-stacking interactions between the pyrene group of the bpea-pyrene ligand and the GO and (ii) both on glassy carbon electrodes (GC) and on graphite rods (GR) through oxidative electropolymerization of the pyrene group, which yield stable heterogeneous photoredox catalysts. GO@trans-fac-**3**- and GR/poly trans-fac-**3-**modified electrodes were fully characterized by spectroscopic and electrochemical methods. Trans-fac-**3** and GO@trans-fac-**3** photocatalysts (without a photosensitizer) showed good catalytic efficiency in the photooxidation of alcohols in water under mild conditions and using visible light. Both photocatalysts display high selectivity values (>99%) even for primary alcohols in accordance with the presence of two-electron transfer processes (2e^–^/2H^+^). GO@trans-fac-**3** keeps intact its homogeneous catalytic properties but shows an enhancement in yields. GO@trans-fac-**3** can be easily recycled by filtration and reused for up to five runs without any significant loss of catalytic activity. Graphite rods (GR@trans-fac-**3**) were also evaluated as heterogeneous photoredox catalysts showing high turnover numbers (TON) and selectivity values.

## Introduction

One of the great challenges in catalysis is the development of high-efficiency selective and sustainable catalysts. The conversion of solar energy into chemical energy inspired by photosynthesis^1,2^ is one of the main pillars of sustainable catalytic processes in the search for clean energy sources.^3,4^ Moreover, mimicking processes that occur in nature through photoredox catalysis is one step forward in the development of a sustainable and green chemistry.^5^

The photooxidation of organic substrates such as alcohols has a particular interest in the development of renewable clean energy as occurs with hydrogen. Both systems involve a two-electron two proton-coupled process.^6^ Cooperative photoredox catalysis based on ruthenium compounds are the systems most commonly studied in photooxidation of organic substrates, which involve a photocatalyst, acting as the light-harvesting antenna, combined with a transition metal catalyst, which can activate the organic substrate through proton-coupled electron transfer (PCET) mechanisms.^7−11^ However, in photooxidation catalysis, there are few examples where only one photocatalyst participates in the process^12−15^ and, specifically, few ruthenium photocatalysts based on polypyridyl ligands acting as both photosensitizers and catalysts have been described in the literature^16−20^ and scarcely are those based on ruthenium aqua complexes.^21,22^

It is known that catalytic processes where the oxidation of the Ru(II)-aqua to the Ru(IV)-oxo catalytic species involves two one-electron processes display low or lack of selectivity in the resulting oxidation products, because of the presence of radical species in the reaction pathways.^23^ Therefore, developing Ru(II)-aqua complexes, in which two proton-coupled two-electron transfer processes occur, is desirable to obtain more selective catalysts.

From the perspective of sustainability and industrial large-scale applications, the development of efficient and reusable heterogeneous photocatalysts is of interest owing to their advantages of simple workup, reduced cost and pollution, and continuous work.^24^ Moreover, the anchoring of the homogeneous catalysts on supports can minimize their deactivation and increase their performance.

Among the different strategies of catalyst immobilization, the noncovalent functionalization of supports such as GO^25−27^ stands out, as it often involves π–π interactions between the support and metal complexes containing ligands functionalized with aromatic groups. Noncovalent functionalization leads to an enhancement of reactivity, binding capacity, dispersibility, and biocompatibility, among others. Graphene and its derivatives are two-dimensional nanostructured supports with high specific area and minimal mass transfer resistance. These materials are of considerable interest due to their low cost, excellent thermal and chemical stability, and rich surface chemistry. GO is both hydrophilic and easily dispersible in water. Additionally, it contains hydrophobic domains on the basal plane.^28^ Supported photocatalysts on GO exhibit an improved photocatalytic performance since the support prevents the recombination of charge, facilitating the electron transfer.^29^ Few metal complexes containing ligands functionalized with the pyrene group have been anchored on GO.^30,31^ The various photocatalysts supported on GO described in the literature, nanoparticles, TiO_2_, quantum dots, and metal clusters are fundamentally highlighted.^32,33^ Some of these exhibit significant environmental applications, particularly in the elimination of persistent organic pollutants for wastewater.^34^

Electropolymerization is another strategy for the immobilization of catalysts that involves the formation of a polymeric film containing the catalyst on the surface of electrodes upon oxidation or reduction. Graphite rods are carbon-based electrodes with low cost, simplicity, and commercial availability.^35,36^ Considerable research effort has been done on developing electrochemically active polymer materials, including those based on pyrene for its applications in electrochemical energy storage.^37^ However, no examples of electropolymerization of pyrene monomers on graphite electrodes are known and, so far, pyrene-based ruthenium aqua complexes have never been anchored on graphite electrodes, nor have the resulting systems been applied as oxidation photocatalysts. Based on the current state of knowledge, our goal is to design sustainable and selective photocatalysts using a ruthenium aqua complex containing an *N*-tridentate ligand modified with the pyrene group. We aim to explore the potential of the heterogenization of these catalysts through a noncovalent interaction between the pyrene group and GO support. Additionally, we plan to investigate their electrosynthesis on both glassy carbon electrodes and graphite rods. Moreover, we want to study their performance as photocatalysts in alcohol oxidation in water under visible light.

With all these considerations in mind, this paper presents an alternative and straightforward synthetic route for the functionalized *N*-tridentate ligand 1-[bis(pyridine-2-ylmethyl)amino]methylpyrene (bpea-pyrene), together with the synthesis and full characterization of the molecular ruthenium compounds trans-fac-[Ru^II^(bpea-pyrene)(bpy)X]^*n*+^ (trans-fac-**2**, X = Cl, *n* =1; trans-fac-**3**, X = H_2_O, *n* =2), with (bpy) being the bidentate bipyridine ligand. Complex trans-fac-**3** has been immobilized on GO via π-stacking interactions, and the resulting hybrid system, GO@trans-**3**, has been characterized. Both trans-fac-**2** and trans-fac-**3** ruthenium complexes have been anchored on both GC and GR electrodes, upon anodic oxidation of the pyrene group of bpea-pyrene ligand, generating the corresponding Ru-based metallopolymers. In addition, we present a comprehensive analysis of the performance of both homogeneous and various heterogeneous Ru aqua photocatalysts for alcohol oxidation reactions, all conducted in water without the need for an additional photosensitizer. We expect that anchoring our catalyst to GO will translate into an improvement of its performance in terms of higher yields, due to a better interaction between the different substrates with the active center of our catalyst and, as we have commented previously, also due to the easier electronic transfer. This prevents the recombination of the electron–hole pair during light excitation, which would also increase the corresponding yields. Furthermore, we explore the potential for the reutilization of the corresponding Ru aqua-supported photocatalysts and propose a plausible mechanism to elucidate the observed reactions.

## Results and Discussion

### Synthesis and Structural Characterization

#### Ligand and Molecular Complexes

**Scheme 1:**
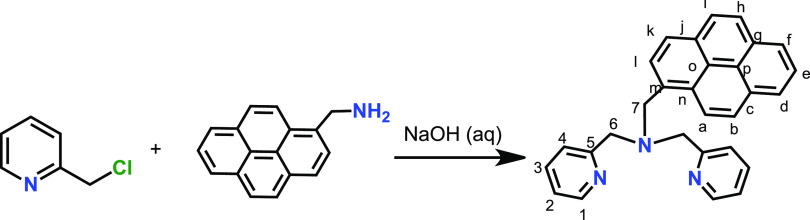
Synthetic Strategy for the Preparation of bpea-Pyrene

**Scheme 2:**
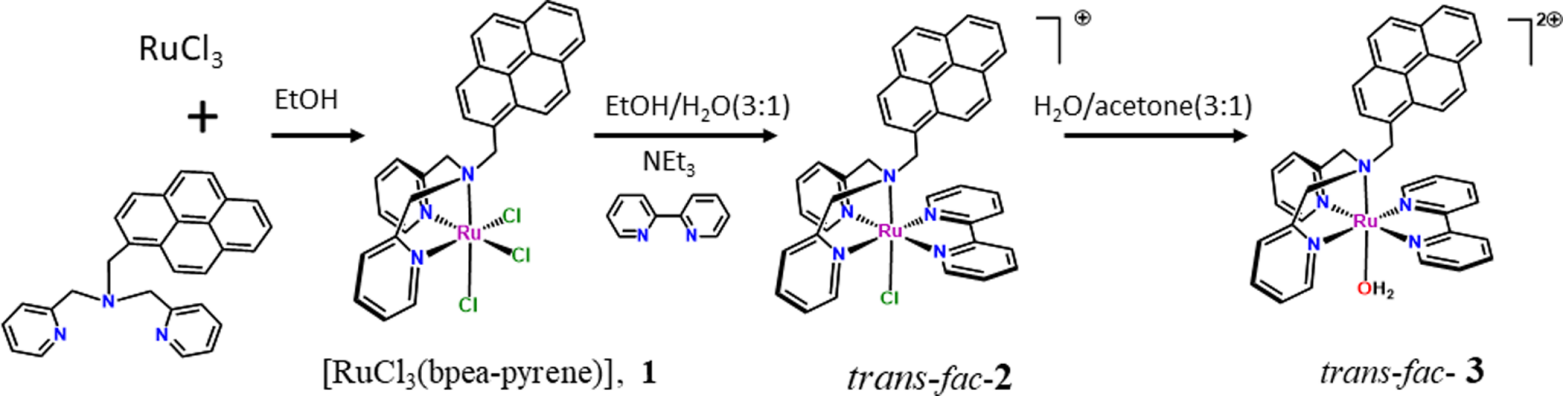
Synthetic Strategy for the Preparation of **1**, Trans-fac-**2**, and Trans-fac-**3** Complexes

The ligand bpea-pyrene was obtained following a different method to literature procedures^38^ (Scheme 1). The synthetic preparation of the complexes is displayed in Scheme 2. The addition of the ligand bpea-pyrene to RuCl_3_ salt leads to the formation of complex [RuCl_3_(bpea-pyrene)], **1**, which is used as starting material for the preparation of complex trans-fac-**2**. Reaction of an equimolar amount of **1** and the ligand 2,2′-bipyridine (bpy) in EtOH:H_2_O (3:1) at reflux in the presence of Et_3_N resulted in the formation of the chlorido Ru^II^ complex trans-fac-**2**, which was isolated as the salt of [PF_6_ ]^−^ after the addition of a saturated NH_4_PF_6_ aqueous solution and purification through a chromatography column. The corresponding Ru–OH_2_ complex trans-fac-**3** is easily obtained from the corresponding Ru–Cl in water/acetone (3:1), in the presence of AgNO_3_ after 4 h of reaction. Although the complex can also be obtained by dissolving the chlorido complex in a mixture of water and acetone and refluxing it for 8 h without the addition of Ag^+^ ions, as we have previously done with other Ru compounds,^39^ some of them containing the pyrene group, it leads to increased lability of the Cl ligand.^22^

The flexibility of the *N*-tridentate bpea-pyrene ligand allows it to act either as a meridional (mer-) or as a facial (fac-) ligand when co-ordinating to a ruthenium metal center (see Scheme S1). When the ligand is coordinated in a facial way, the monodentate ligands (Cl^–^ or H_2_O) can be located trans or cis with regard to the aliphatic nitrogen (N_al_) of the ligand, and other different stereoisomers could be obtained, the trans-fac and cis-fac; the nomenclature trans- or cis- refers to the relative position of the monodentate ligands, Cl^–^ or H_2_O. In both cases, for the chlorido and aqua complexes, we have detected a single isomer the trans-fac (see below) being the coordination fac-the thermodynamically stable arrangement of the bpea-pyrene ligand in this kind of compounds.^40^

Complex trans-fac-**2** has been characterized by single-crystal X-ray since suitable single crystals were obtained by diffusion of diethyl ether into a CHCl_3_ solution.

**Figure 1:**
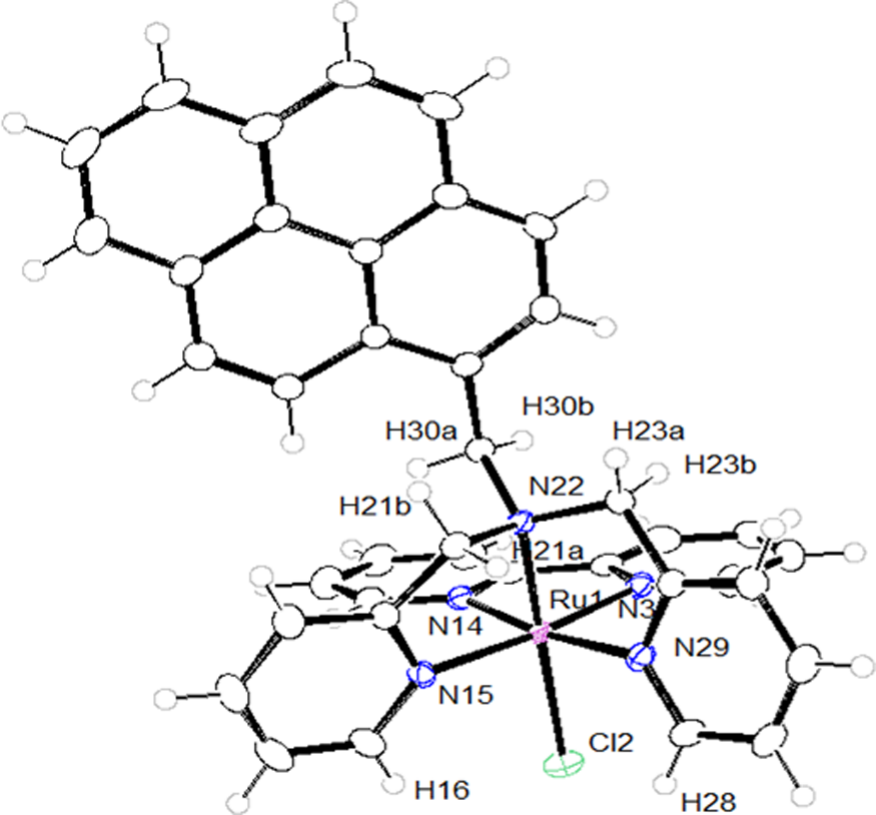
Molecular structure and labeling schemes for trans-fac-**2**.

Figure 1 displays the molecular structure and Figures S1 and S2 the hydrogen bond interactions and the packing arrangements, respectively. Tables S1 and S2 show the main crystallographic data and selected bond distances and angles. The description of the structure is shown in the SI.

#### Spectroscopic Characterization

Characterization of ligand bpea-pyrene and trans-fac-**2** and -**3** complexes was done through IR spectra and one- (1D) and two-dimensional (2D) NMR spectra (Figures S3–S6). The solid IR spectra obtained for the ligand and complexes (Figure S3) show vibrations around 3100–2700 and 1600–1000 cm^–1^, which can be respectively assigned to ν_N–H_, ν_=C–H_, and ν_C=N_ stretching modes of the polypyridyl ligands. The spectrum of trans-fac-**3** displays an additional band centered at ca. 3315 cm^–1^, which corresponds to the υ_O–H_ stretching vibration of the water coordinated to ruthenium.

The ^1^H NMR spectrum of the bpea-pyrene ligand shows two sets of resonances, one in the aromatic region corresponding to the pyrene and pyridylic protons, and the resonances corresponding to the benzylic (H6) and methylene (H7) protons appear as two singlets at 3.9 and 4.4 ppm corresponding to four and two hydrogen atoms, respectively (Figure S4).

When the ligand bpea-pyrene is coordinated to the ruthenium metal in both complexes, the resonances observed are in accordance with the presence of the trans-fac isomers, consistent with the structure observed in the solid state. For the trans-fac isomers, the molecules display a plane that contains the Ru atom, the monodentate ligand (Cl^–^ or H_2_O), and the aliphatic nitrogen, all the pyridyl rings being equivalent, and therefore only one set of aromatic resonances should be observed in NMR for both ligands, bpy and bpea-pyrene ligands. However, the resonances corresponding to the benzylic protons become magnetically different (H6a and H6b), appearing as two doublets due to their different environments. The assignation of these hydrogen atoms (Figure S5) has been done through the NOE observed between H6a and H8, in both compounds (see Figures S5d and S6c). The two H6a correspond to H23b and H21b in the crystal structure of trans-fac-**2** (Figure 1). However, the resonances corresponding to the methylene protons H7 in trans-fac-**2** and trans-fac-**3** appear as singlets, which evidence a similar magnetic environment for these atoms because of free rotation of the N_al_-C bond of the pyrene group.

It is worth mentioning the deshielding effect exerted by the chlorido ligand over the pyridylic protons H1 of the ligand (δ = 9.7 ppm) in trans-fac-**2** isomer (Figure S5a) with regard to the isomer trans-fac-**3** (δ = 9.4 ppm), which appears upfield influenced by the lower deshielding exerted by the water ligand (Figure S6a).

The UV–vis spectra of the complexes in CH_2_Cl_2_ (Figure S7) show the ligand-based π–π* bands below 350 nm and the dπ(Ru)-π*(L) MLCT transitions above 350 nm. For the Ru–Cl complex, the MLCT band is shifted to the red region due to the lower stabilization of the dπ(Ru) levels caused by the Cl ligand in comparison with the OH_2_ ligand. In both compounds, the presence of an alkyl pyrene substituent at the *N*-tridentate ligand induces an hypsochromic shift of the MLCT absorptions with regard to the analogous complex bearing the bpea ligand,^41,42^ which is consistent with the lower electron density donation from the ligand to the metal. A similar behavior is observed in other complexes containing the pyrene group.^22^

#### Electrochemical Properties

**Figure 2:**
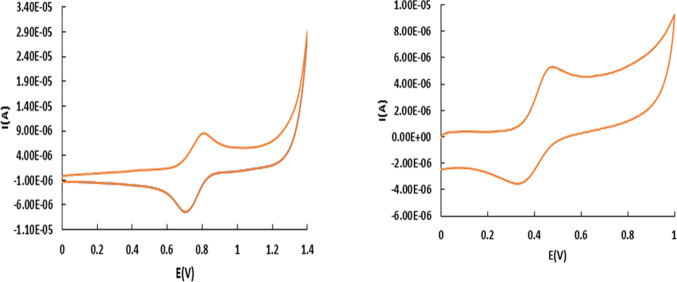
(left) CV of trans-fac-**2** in CH_2_Cl_2_ (TBAH 0.1 M) vs SCE and (right) trans-fac-**3** in phosphate buffer (pH= 6.8) vs SCE.

Electrochemistry of the ligand bpea-pyrene and complexes trans-fac-**2** and **3** was done in CH_2_Cl_2_ + 0.1 M tetrabutylammonium hexafluorophosphate (TBAH) or phosphate buffer (pH = 6.8) using glassy carbon electrodes as working electrodes. Figure S8 shows the CV of the bpea-pyrene ligand with three irreversible peaks at 1.11, 1.33, and 1.45 V vs SCE. The peak at 1.11 V corresponds to the oxidation of the aliphatic nitrogen of the ligand, and the peak at 1.45 V most probably is due to the oxidation of the pyridine nitrogen. This agrees with the electrochemical behavior observed for the free ligand bpea and the greater π-acceptor character of the bpea-pyrene ligand.^43^ Then, the peak at 1.33 V is assigned to the electro-oxidation of the pyrene monomer to its cationic radical.^44^Figure 2 and Figure S9 show the cyclic voltammetry (CV) and differential pulse voltammetry (DPV) experiments for the chlorido trans-fac-**2** and aqua trans-fac-**3** complexes, respectively. For both complexes, a quasireversible oxidation wave was observed; in the case of trans-fac-**2** assigned to the Ru(III)/Ru(II) redox couple, *E*_1/2_ = 0.75 vs SCE and, in the case of trans-fac-**3**, the wave is assigned to the Ru(IV/II) bielectronic redox process at *E*_1/2_ = 0.40 vs SCE at pH = 6.8. This behavior has been observed through a wide pH range in aqueous medium (Figure S10), which is in accordance with the simultaneous transfer of two-electron and two-proton, 2e^−^/2H^+^ or two overlapping 1e^−^/1H^+^,^45^ and it has been observed previously with other N-tridentate ligands.^46^ This redox behavior is attributed to the disproportionation of the Ru^III^–OH species, due to the presence of strong π-accepting ligands as the bpea-pyrene. Both compounds display an irreversible oxidation peak around 1.3–1.4 V corresponding to the irreversible oxidation of the pyrene group. The electrochemistry of trans-fac-**3** in CH_2_Cl_2_ displays also one quasireversible process at *E*_1/2_ = 1.11 vs SCE (see Electropolymerization onto GC Electrodes Section).

#### Functionalization of GO with Trans-fac-**3**

**Scheme 3:**
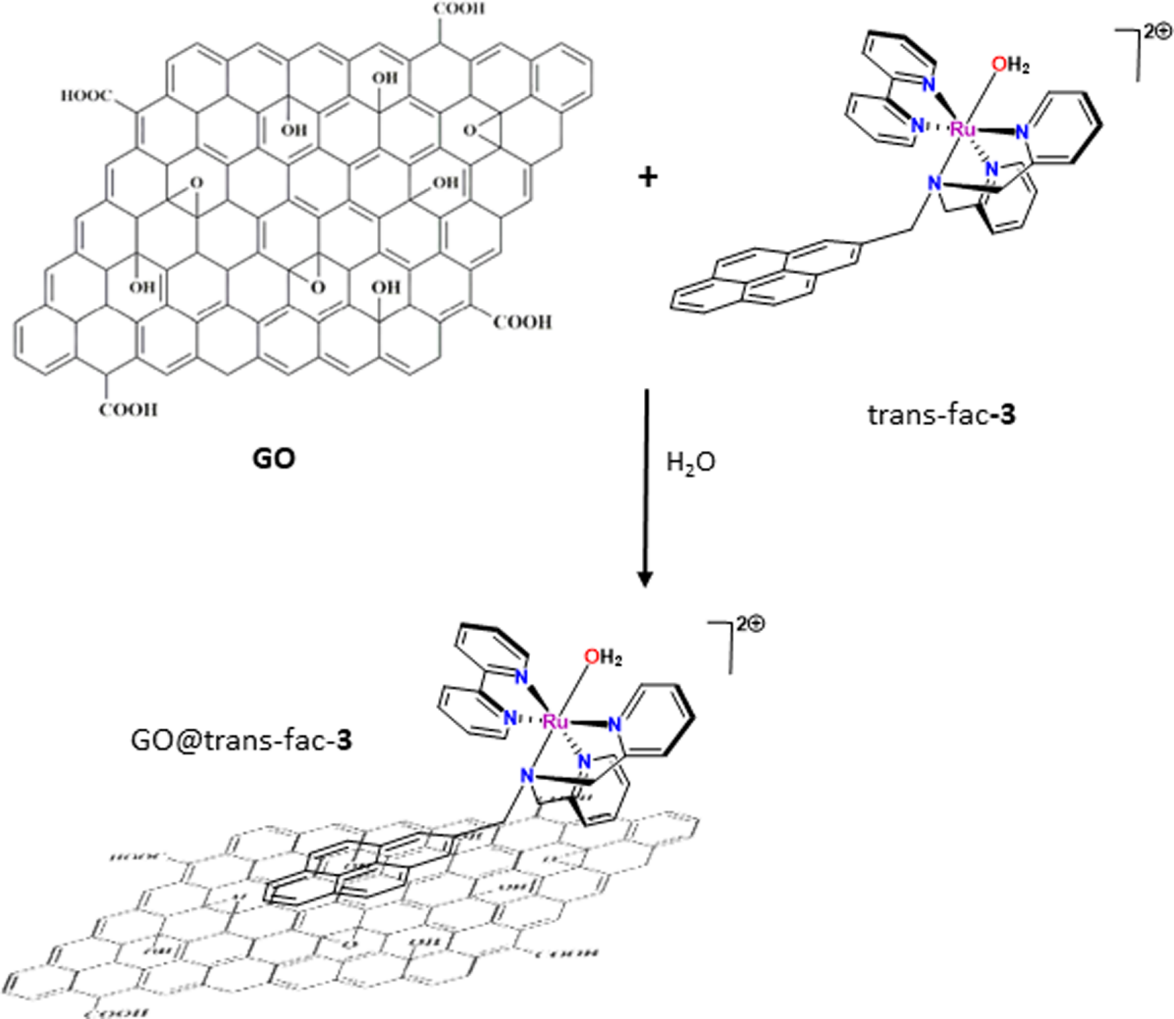
Synthetic Strategy Used for the Immobilization of Trans-fac-**3** onto GO

The trans-fac-3 molecular aqua ruthenium complex was supported on graphene oxide (GO) in one step, as is shown in Scheme 3. The synthetic strategy consisted in the preparation of a dispersion of 50 mg of GO in water (20 mL) that was sonicated for 30 min; afterward, 24 mg of complex trans-fac-**3** was added. The dispersion was stirred for 12 h at RT to afford the immobilized complex GO@trans-fac-[Ru(bpea-pyrene)(bpy)OH_2_](PF_6_)_2_; GO@trans-fac-**3** was filtered, washed with water, and dried. GO@trans-fac-**3** can also be obtained using dichloromethane as solvent, but the amount of the immobilized molecular aqua complex was lower than in the case of using water as solvent. The synthetic strategy is based on the formation of π-stacking interactions between the pyrene group of the bpea-pyrene ligand in the aqua complex trans-fac-**3** and the GO support, leading to the anchoring of compound to GO in a noncovalently bonded way. To the best of our knowledge, this is the first example of a molecular aqua ruthenium complex anchored on GO support (see below) using an easy and green synthetic method, since in previous works a heterogeneous aqua complex was generated by dissolving in water the chlorido precursor supported previously on reduced graphene oxide (rGO).^22^ The ruthenium complex loaded in GO@trans-fac-**3** was measured by inductively coupled plasma-atomic emission spectrometry (ICP-AES), and the amount anchored was 22.01 μmol/100 mg of GO for the synthesis done in water and 9.7 μmol/100 mg when the synthesis was in dichloromethane. In parallel, the UV–vis spectrum of trans-fac-**3** in water corroborated that after 24 h of reaction, 94% of molecular complex was anchored to GO (Figure S11).

**Figure 3:**
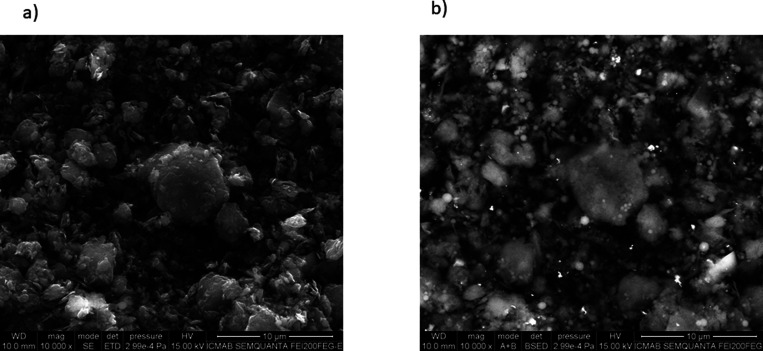
SEM images (a) using an ET detector and (b) using a BSE detector of GO@trans-fac**-3**.

To validate the successful synthesis of the hybrid material, GO@trans-fac-**3**, it was characterized using various techniques, such as transmission electron microscopy (TEM), scanning electron microscopy (SEM), X-ray photoelectron spectroscopy (XPS), UV–vis, and DPV. The TEM image of the supported complex (Figure S12a) and SEM images of the naked GO support (Figure S12b) together with the support after functionalization (Figure 3 and Figure S12c) were taken to know the morphology of the composite.

**Figure 4:**
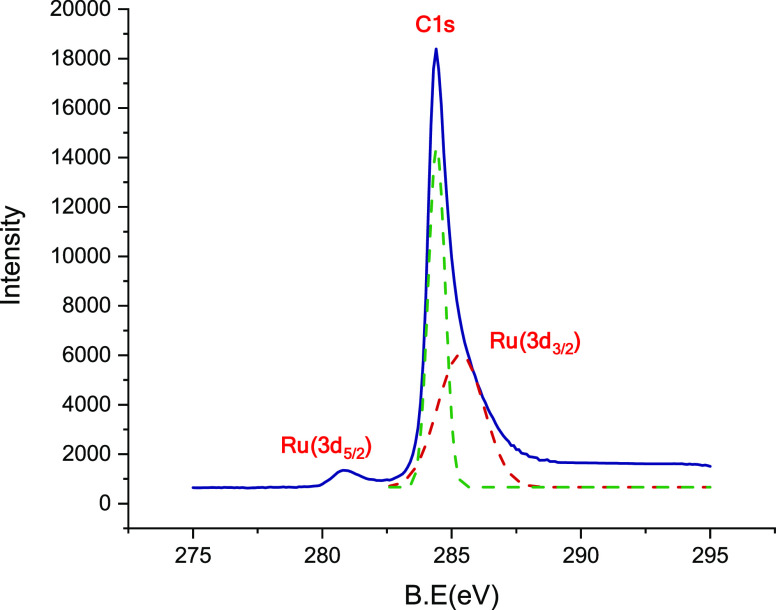
XPS spectrum of GO@trans-fac**-3** in the C 1s, Ru(3d_3/2_), and Ru(3d_5/2_) regions.

The images reveal that the hybrid material shows structures with a roughened surface and with irregular shapes. The use of a BSE (back scattered electron) detector (Figure 3b) allows to ensure the presence of ruthenium on the GO (white spots), and the energy-dispersive X-ray spectroscopy (EDX) analysis showed the existence of the uniform distribution of ruthenium in the modified support (Figure S13). XPS displays the surface composition of the GO@trans-fac**-3** (Figure 4 and Figure S14) corroborating the presence of C, O, N, and Ru. The spectra display the strong peak of C 1s at ∼284 eV that corresponds to C=C, C=O, and C–OH present in the support and complex. At 532 eV, the peak of O 1s of the oxygen present in the GO appears and the peak of N 1s present in the complex appears at ∼400 eV. The peaks at ∼281 and ∼286 eV were identified due to Ru(3d_5/2_) and Ru(3d_2/3_). The deconvolution of this last peaks near the C 1s signal has been done (Figure 4). Another peak observed at ∼463 eV could be assigned to Ru(3p_3/2_) (Figure S14). These values of binding energies observed in the XPS spectra for ruthenium reveal the presence of Ru(II) species in the hybrid material.^47,48^

The UV–visible spectrum of GO@trans-fac-**3** registered on a suspension of the heterogeneous support in dichloromethane exhibits a similar pattern to that of the homogeneous compound, observing the band corresponding to dπ(Ru)-π*(L) MLCT transitions around 460 nm, in agreement with that observed in the aqua complex trans-fac-**3** (Figure S15).

**Figure 5:**
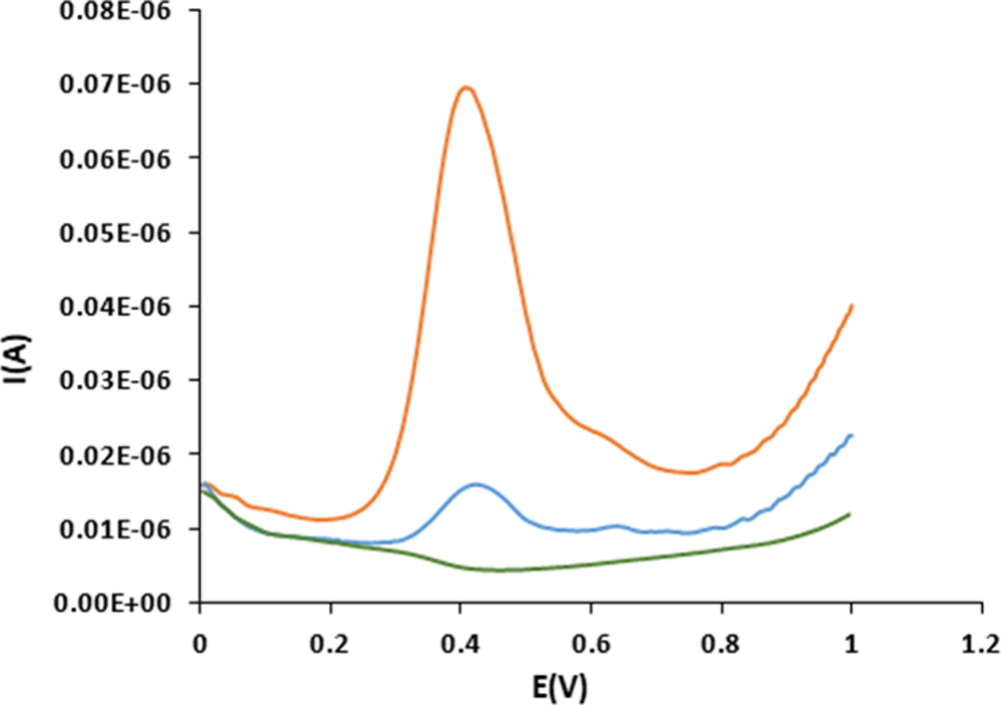
DPV of trans-fac-**3** (orange line), GO@trans-fac-**3** (blue line), and GO (green line) in phosphate buffer (pH 6.8) vs SCE (reference electrode). Glassy carbon electrode (3 mm diameter) was used as working electrode, platinum wire as auxiliary.

The electrochemical behavior of GO@trans-fac-**3** was studied by DPV. Figure 5 shows the DPV curves of the immobilized aqua complex GO@trans-fac**-3**, registered in phosphate buffer, together with that of homogeneous trans-fac**-3** for comparison. The DPV of the black solid displays a wave about 0.4 V vs SCE, at pH = 6.8, corresponding to the Ru(IV/II) redox couple, in accordance with the electrochemical behavior presented by the homogeneous complex (see above). This result evidences that the aqua complex has been satisfactorily immobilized on the surface of the GO support, with the labile aqua ligand remaining intact and their redox properties maintained after the anchorage.

### Functionalization of GC and GR with Trans-fac-**2** and Trans-fac-**3** Complexes

Complexes trans-fac-**2** and trans-fac-**3** can undergo oxidative electropolymerization leading to the formation of polymerized pyrene films on the surface of GC electrodes (3 mm diameter) or GR (1 cm height, 3.15 mm diameter).

#### Electropolymerization onto GC Electrodes

We have investigated the electrosynthesis of poly-trans-fac-**2** at the surface of the GC electrode by 10 successive scans between 0 and 1.4 V in a 1 mM solution of trans-fac-**2** in CH_2_Cl_2_ + 0.1 M TBAH, *v* = 100 mV·s^–1^. The formation and growth of the polymer are confirmed by the increase of the oxidation waves corresponding to the reversible Ru(III)/Ru(II), *E*_1/2_ = 0.75 V vs SCE, Δ*E* = 130 mV (Figure S16a). One small new peak appears at 0.57 V, which could be assigned to the electroactivity of the polypyrene backbone.^49^Figure S16b depicts the response of the GC/poly trans-fac-**2**-modified electrode upon immersion in a fresh electrolyte solution. Again, an electrochemical response is observed for the polypyrene backbone *E*_pa_ = 0.62 V and for the Ru(III)/Ru(II) redox couple at *E*_1/2_ = 0.75 V (Δ*E* = 70 mV). The film obtained is stable after repeated scanning over the potential range of 0 to 1 V. In the second scan, the intensity of the anodic peak slightly decreases, but in the following scans, the intensity of both anodic and cathodic peaks remains practically constant for the following six cycles. Figure S17 shows the electrochemical response of the film obtained to different scan rates and the linear dependence of the oxidation and reduction wave intensity as a function of the scan rate, the latter indicating the stable anchorage of the complex onto the electrode surface. The amount of polymerized complex on the electrode surface was of Γ = 1.0 × 10^–**9**^ mol cm^–2^ and was calculated by integration of the charge of the Ru(III/II) anodic peak.

**Figure 6:**
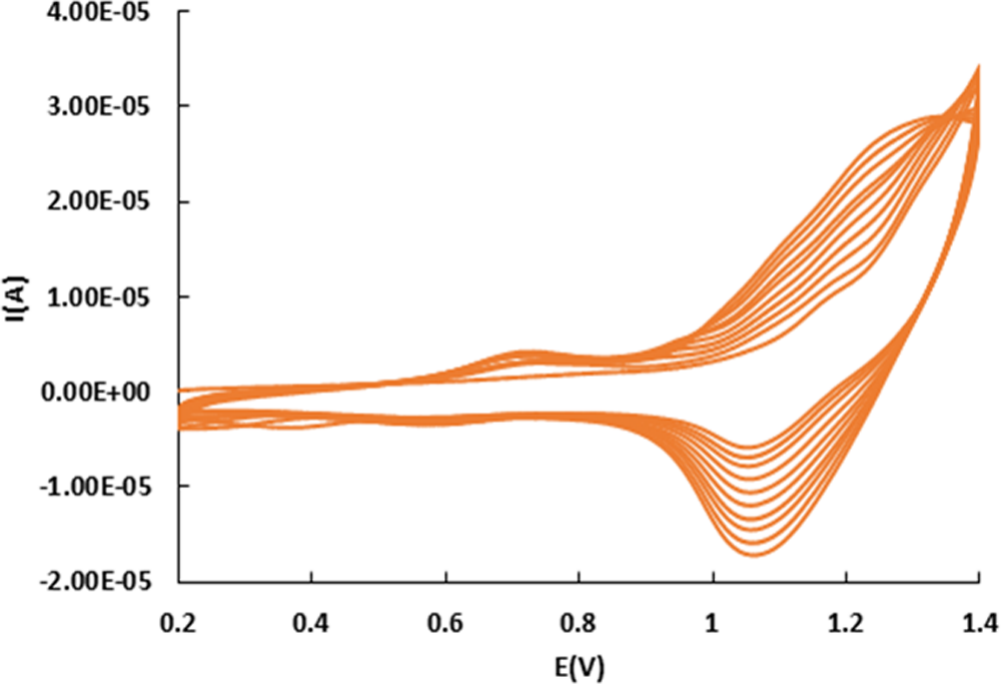
CV for the electropolymerization of trans-fac-**3** on a GC electrode.

The voltammograms recorded during the electropolymerization of trans-fac-**3** onto GC electrodes are displayed in Figure 6. Electropolymerization was carried out through 10 successive scans between 0 and 1.4 V in a 0.5 mM solution of trans-fac-**3** in CH_2_Cl_2_ + 0.1 M TBAH, *v* = 100 mV·s^–1^ with the formation of the GC/poly**-**trans-fac-**3**-modified electrode. The first scan shows a reversible system at *E*_1/2_ = 1.11 vs SCE, followed by an irreversible oxidation of a pyrene group at *E*_pa_ = 1.3–1.4 V. The first one single oxidation wave of the aquo complex corresponds to the oxidation of Ru(II) to Ru(IV), with a Δ*E* = 67 mV; this value is approximately half of that obtained for the chloride complex in the same medium, suggesting the transfer of two electrons, consistent with the results previously obtained in a buffer medium. The confirmation of the electropolymerized film on the electrode surface is indicated by a 10-scan increase in the intensity of the Ru(IV)/Ru(II) peak, attributed to the oxidative electropolymerization of the aqua complex. This process is accompanied by the development of a quasireversible system at *E*_1/2_ = 0.65 V vs SCE, potentially corresponding to the electroactivity of the polypyrene skeleton, thereby substantiating the formation of the GC/poly-trans-fac-**3**. After transferring the electrode to a metal-free solution, the CV displayed two closely located waves corresponding to the electroactivity of ruthenium aqua-polymer (Figure S18). This fact shows that the redox properties of the polymerized aqua complex has been slightly modified with respect to the redox behavior presented by the molecular trans-fac-**3** dissolved in dichloromethane. This behavior has been observed in other electropolymerized ruthenium compounds.^50^ The amount of polymerized complex onto the electrode surface was of Γ = 0.73 × 10^–**9**^ mol cm^–2^.

#### Electropolymerization onto GR

**Figure 7:**
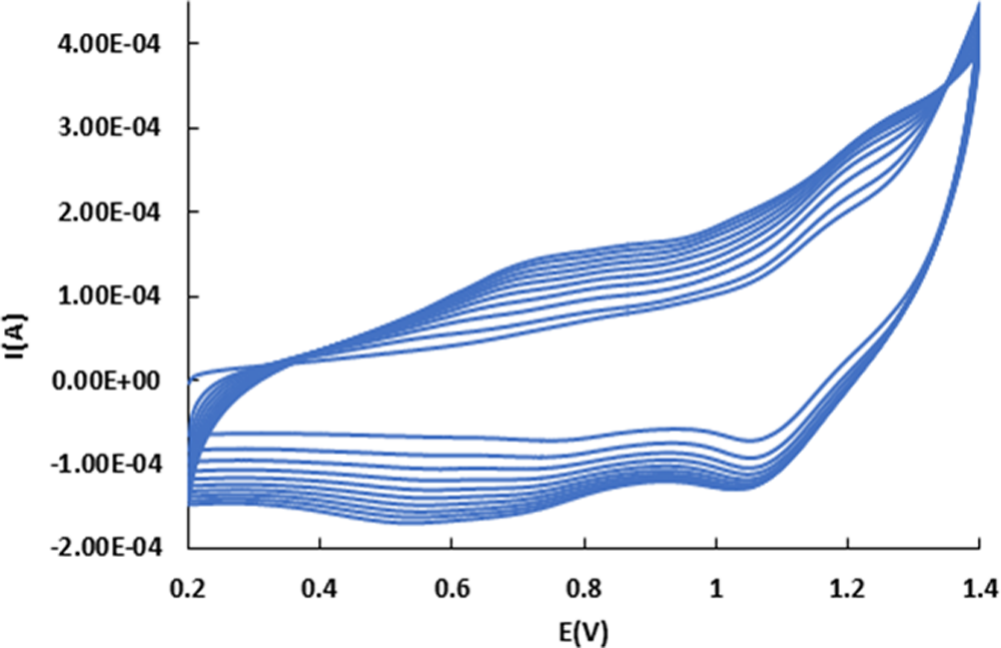
CV for the electropolymerization of trans-fac-**3** on GR.

We have used graphite rods of 1 cm height and 3.15 mm diameter to polymerize both the chlorido and the aqua ruthenium pyrene compounds. Electropolymerization was made by 10 and 15 successive scans between 0 and 1.4 V in a 1 mM solution of trans-fac-**2** or -**3** in CH_2_Cl_2_ + 0.1 M TBAH at *v* = 20 mV·s^–1^, respectively. The formation and growth of the polymers GR/poly**-**trans-fac-**2** or -**3** are confirmed by the increase of the oxidation waves corresponding to the reversible Ru(III)/Ru(II), *E*_1/2_ = 0.75 V vs SCE, for trans-fac-**2** and to the Ru(IV)/Ru(II), *E*_1/2_ = 1.11 V vs SCE for trans-fac-**3** (Figure S19 and Figure 7, respectively).

The SEM images obtained for GR/poly**-**trans-fac-**3** show the morphology of the generated polymer onto the graphite surface (Figures S20), with the irregular coating formed on the graphite and the formation of agglomerates. The use of a BSE detector (Figure S20b) allows to ensure the presence of ruthenium on the graphite surface (white spots). This image reveals a homogeneous distribution of the trans-fac-**3** aqua complex on the surface of the graphite rods.

### Photocatalytic Oxidation

We have studied the photocatalytic activity of the molecular aqua complex trans-fac-**3** and the hybrid heterogeneous systems GO@trans-fac-**3** and GR/poly-trans-fac-**3**, all acting as both photosensitizers and catalysts, in the oxidation of several alcohols in water. For both trans-fac-**3** and GO@trans-fac-**3**, the experiments were conducted by exposing a solution containing 2.5 mL of water (K_2_CO_3_ pH = 7), the substrate, and 1 mol % of catalyst to visible irradiation, in the presence of Na_2_S_2_O_8_ as an oxidizing agent. The reactions were carried out at room temperature and atmospheric pressure for 6 and 8 h. Then, the reaction products were extracted with dichloromethane three times, dried using Na_2_SO_4_, and quantified by means of ^1^H NMR spectroscopy. The results were further confirmed through GC-MS analysis.

Initially, we investigated the photooxidation of 1-phenylethanol using a phosphate buffer at pH = 7 as the reaction medium. However, the yield obtained was slightly lower (52%) compared to using only water and K_2_CO_3_ (55%). Consequently, we opted to use the latter medium due to its economic and practical advantages.

We tested various reaction times using 1-phenylethanol as the substrate (Figure S21) and determined that conducting the catalytic experiments for 6 and 8 h was optimal. Control experiments demonstrated that no significant oxidation of alcohol occurred in the absence of photocatalyst, light, or oxidizing agent after 6 h of reaction. Additionally, a blank control using the naked GO as a catalyst under the same conditions was carried out; in all cases, the conversion was below 10%.

Also, we have tested two different concentrations of trans-fac-**3** as a photoredox catalyst, 0.25 and 0.49 mM, for 8 h, maintaining constant the concentrations of substrate (1-phenylethanol, 49 mM) and Na_2_S_2_O_8_ (98 mM); these results showed that an increase of acetophenone is produced at higher load of the catalyst (34 vs 55%). Thus, we have taken the highest tested concentration of photocatalyst (0.49 mM) as the optimal one to pursue the study toward other alcohols. We have also observed that, maintaining these conditions but decreasing the amount of oxidizing agent to 74 mM, the amount of acetophenone decreased to 40%. In all cases, we have observed an increase in acidity in the reaction medium after the catalysis.

Table 1 shows the results of photocatalytic activity of the molecular trans-fac-**3** compound and the heterogeneous GO@trans-fac-**3** compound. In general, moderate yields have been achieved in the photooxidation of 1-phenylethanol (entry 1), benzyl alcohol (entry 2), and 4-methylbenzyl alcohol (entry 3) by trans-fac-**3** after 6 h. The presence of an electron-donating substituent in the aromatic ring of the benzyl alcohol enhanced the yield of the corresponding aldehyde. When operating with all components and under light, we observed the formation of aldehyde (for the primary benzyl alcohols) or ketone (for the secondary alcohols) as the sole product of the oxidation reaction, achieving a remarkable selectivity of >99%.

**Table 1:**
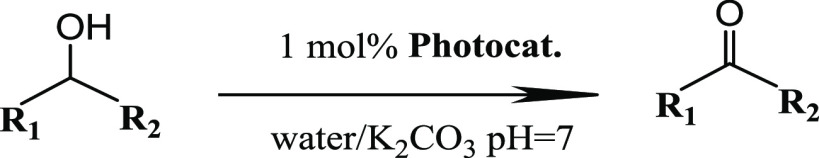

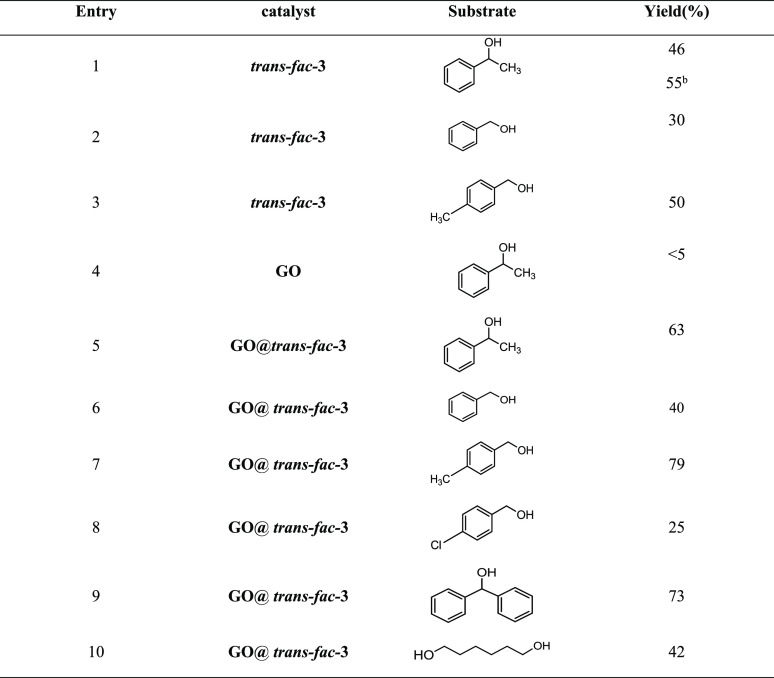
Photocatalytic Oxidation of Alcohols^a^

a Conditions: catalyst (0.49 mM), substrate (49 mM), Na_2_S_2_O_8_ (98 mM), 2.5 mL of water (K_2_CO_3_ pH 7), 6 h of catalysis at RT.

b After 8 h of catalysis. Light irradiation using a xenon lamp with λ = 400–700 nm.

The heterogeneous GO@ trans-fac-**3** exhibited superior performance compared to the homogeneous trans-fac-**3** in the photooxidation of various primary and secondary aromatic alcohols, generally producing good yields. This enhancement in photocatalytic performance suggests that supporting the photocatalyst on GO could facilitate electron transfer and prevent the recombination of hole–electron pairs formed during light excitation.^29^ Another possible explanation could be a lower deactivation of the catalyst when it is supported on GO. These results demonstrate the positive effect of the GO support on the photooxidation catalysis. We can observe that secondary aromatic alcohols as 1-phenylethanol (entry 5) and diphenylmethanol (entry 9) were found to be more reactive than the primary benzyl alcohol (entry 6). Similar to the observations with the molecular photocatalyst, the yield value was enhanced when an electron-donating methyl substituent was present on the aromatic ring of the benzyl alcohol (entry 7). Conversely, the yield decreased in the presence of electron-withdrawing substituents such as Cl (entry 8). Under identical reaction conditions, we conducted the photooxidation of an industrially significant diol, namely 1, 6-hexanediol (entry 10). This resulted in a moderate yield of the corresponding diacid at 42%. Notably, the selectivity values for all the products were remarkably high, exceeding 99% in each case.

To verify the occurrence of photoredox catalysis in the heterogeneous phase, we interrupted the photooxidation of 1-phenylethanol after 3 h and removed GO@trans-fac-**3** through filtration. Then, the catalysis was allowed to proceed for an additional 3 h. At the 3 h mark, the yield reached 30% and no further conversion was observed without the presence of the catalyst after the full 6 h. Notably, when the catalysis was complete, we conducted an ICP test on the filtrate solution but no detectable traces of Ru were found. These results confirm that the leaching of Ru from our heterogeneous photocatalyst is negligible.

**Figure 8:**
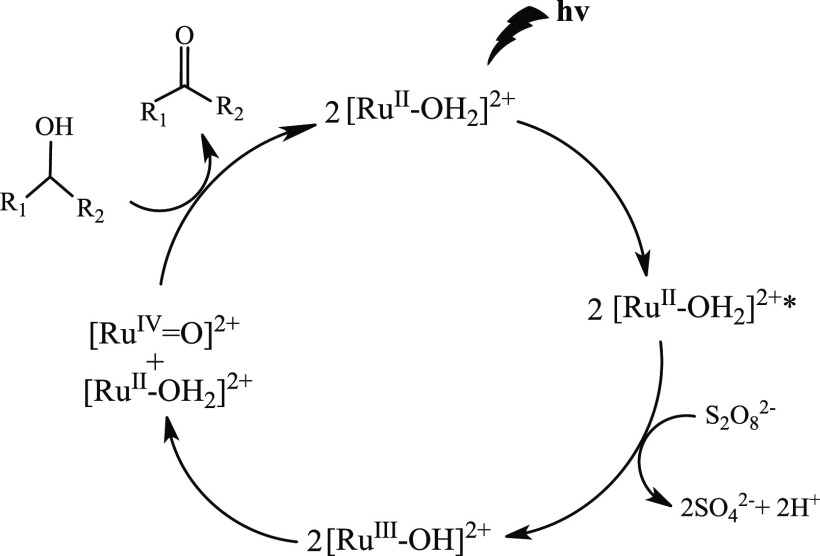
Sugested mechanism of photooxidation.

It is worth mentioning that in both homogeneous and heterogeneous catalysis, the photooxidation of primary alcohols takes place with total selectivity for the corresponding aldehydes, whereas we have observed lower selectivity values with other supported ruthenium aqua complex previously studied.^22^ The observed behavior can be attributed to the thermodynamic instability of the Ru^III^–OH species, which favors two-electron two-proton transfer processes (2e^–^/2H^+^) during the photocatalytic oxidation. Our electrochemical studies have supported this finding, indicating that such processes promote selective photooxidations. On the contrary, the presence of monoelectronic processes favor pathways associated with the presence of radical species that leads to a decrease in selectivity values.^23^ Generally, as the π-acceptor character of the ligands increases, it stabilizes the Ru^II^ species, resulting in higher Ru^III/II^ potentials and lower Ru^IV/III^ potentials. On the basis of the photocatalytic and electrochemical results, the proposed mechanism is consistent with the formation of high-valent Ru(IV)=O species formed via PCET processes (see Figure 8) and it is in agreement with the proposed mechanism by Rocha et al.^21^ The [Ru^II^–OH_2_]^2+^ complex was first activated by visible light to form the excited [Ru^II^–OH_2_]^2+^* species. Then, the oxidative quenching by the sacrificial acceptor S_2_O_8_^2–^ generates the [Ru^III^–OH]^2+^ species that disproportion to [Ru^IV^=O]^2+^ and [Ru^II^–OH_2_]^2+^ both thermodynamically more stable, with the oxo complex being the one that oxidizes the corresponding alcohol. With the proposed pathway, the exchange of (2e^–^/2H^+^) occurs in this photoredox process, leading to an increase in acidity in the catalytic medium, as we have observed in the different catalytic tests.

**Figure 9:**
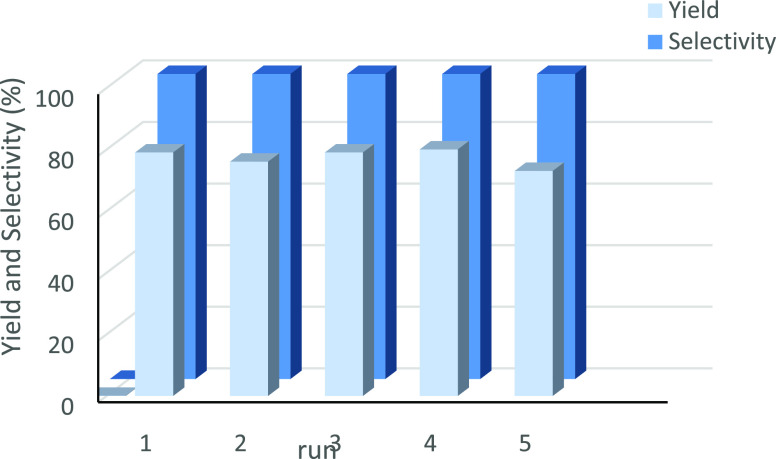
Yield (light blue) and selectivity (blue) values obtained throughout five consecutive reuses of GO@trans-fac-**3** in the photooxidation of 4-methylbenzylalcohol. Conditions: GO@trans-fac-**3** (1.22 μmol), substrate (122 μmol), Na_2_S_2_O_8_ (245 μmol), 2.5 mL of water (K_2_CO_3_ pH 7); light irradiation using a xenon lamp with λ = 400–700 nm. 6 h per run.

One of the notable advantages of the GO@trans-fac-**3** photocatalyst is its ease of recycling and reusability, which contributes to a reduction in heavy metal pollution and overall costs. In light of this benefit, we proceeded to test the photocatalyst in the oxidation of 4-methylbenzyl alcohol in water. The results show (Figure 9) that the hybrid material could be reused at least five times, showing high conversion efficiency (79%) and selectivity (>99%), without significant loss of catalytic activity. Overall turnover numbers of 387 for the photooxidation of 4-methylbenzyl alcohol were achieved. The morphology of the recovered catalyst was analyzed, after five runs, in the photooxidation of 4-methylbenzyl alcohol. The TEM obtained after the catalysis was compared with that of the catalyst before the catalysis (Figure S22). The results show that the morphology is maintained. The SEM images after five runs also corroborate that the morphology is maintained after catalysis (Figure S23).

We have also studied the performance of the obtained modified graphite rods (GR/poly trans-fac-**3**) in the photooxidation of some alcohols (see Table 2). The procedure for the electropolymerization of the complex has been previously described above, and the corresponding amount of ruthenium electropolymerized on each graphite rod is provided in Table 2. The hybrid material demonstrates excellent performance as evidenced by the high yields achieved in the photooxidation of 4-methylbenzyl alcohol (entry 2) and diphenylmethanol (entry 3), although it was slightly lower in the case of 1-phenylethanol (entry 1). Nevertheless, the selectivity observed in all cases was consistently >99%. It is worth mentioning the high TON observed with these systems, being among the highest reported for the photooxidation of alcohols in the heterogeneous phase.^13,22,51,52^ We attempted to reuse the aqua ruthenium graphite rod in the photooxidation of 4-methylbenzyl alcohol. However, the GR@trans-fac-**3** photocatalyst exhibited a remarkable decrease in activity after the third run, with conversions of 68% in the first run, 48% in the second run, and 23% in the third run. To investigate further, we conducted an analysis of the solution after catalysis using ICP spectrometry but no traces of ruthenium were detected. Currently, our laboratory is conducting additional studies to enhance the stability of these systems.

**Table 2:**
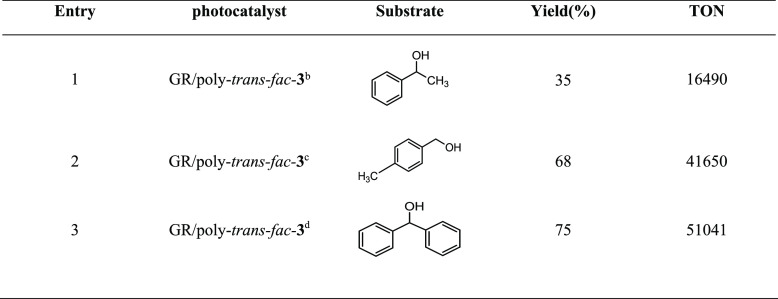
Photocatalytic Oxidation of Alcohols Using the Modified Graphite Rods GR/Poly-trans-fac-**3**^a^

a Conditions: substrate (0,12 mmol), Na_2_S_2_O_8_ (0,24 mmol), 2.5 mL of water (K_2_CO_3_ pH 7), at RT and 6 h of catalysis. Light irradiation using a xenon lamp with λ= 400–700 nm.

b Catalyst: 2.6 × 10^–6^ mmol.

c Catalyst: 2 × 10^–6^ mmol.

d Catalyst: 1.8 × 10^–6^ mmol.

## Conclusions

In summary, this study presents the synthesis and the photocatalytic oxidation behavior of novel homo- and heterogeneous ruthenium aqua complexes in water under visible-light conditions. Trans-fac-[Ru(bpea-pyrene)(bpy)OH_2_](PF_6_)_2_ (trans-fac-**3**) has been obtained from the chlorido complex trans-[Ru^II^Cl(bpea-pyrene)(bpy)]PF_6_ (trans-fac-**2**). In both cases, only a single isomer, the trans-fac, has been isolated. The aqua complex (trans-fac-**3**) can be readily anchored on GO in water forming GO@trans-fac-**3**, through supramolecular π interactions facilitated by the pyrene group of the *N*-tridentate bpea-pyrene ligand. Additionally, both the aqua (trans-fac-**3**) and chlorido (trans-fac-**2**) complexes can be grafted onto GC and GR by electrogeneration of redox polymers, resulting in GC/poly-trans-fac-**2** or **-3** and GR/poly-trans-fac-**2** or -**3**, respectively. Comprehensive spectroscopic, structural, and electrochemical characterizations have been performed on both the homogeneous and heterogeneous complexes.

Trans-fac-**3** and GO@trans-fac-**3** showed catalytic efficiency in the photooxidation of alcohols in water, acting both as oxidation catalyst and as photosensitizer, via proton-coupled electron transfer processes (PCET), displaying total selectivity values for the corresponding aldehydes or ketones, in accordance with the presence of bielectronic processes (2e^–^/2H^+^). The heterogeneous GO@trans-fac-**3** showed an enhancement in yields compared to the homogeneous trans-fac-**3**, probably due to a better electron transfer in the former, facilitated by the GO support. GO@trans-fac-**3** can be readily recycled as it can be easily recovered through filtration and reused at least in five consecutive test runs without a significant loss of its catalytic reactivity.

Modified graphite rods GR/poly**-**trans-fac-**3** were also tested in the heterogeneous photooxidation of some alcohols in water, showing high TON and selectivity values, among the highest reported for the photooxidation of alcohols in heterogeneous phase.

To the best of our knowledge, we have presented the first description and comprehensive study of a molecular ruthenium aqua complex supported on GO and GR. This unique complex serves as an efficient oxidation catalyst and photosensitizer, facilitating the photooxidation of alcohols in water under mild and environmentally friendly conditions. Throughout our research, we have proposed a plausible pathway for these photooxidation reactions.

## Experimental Section

### Materials

All reagents used in the present work were obtained from Sigma-Aldrich and were used without further purification. Reagent-grade organic solvents were obtained from Carlo Erba and high-purity deionized water was obtained by passing distilled water through a nanopure Milli-Q water purification system.

### Instrumentation and Measurements

IR spectra were recorded on an Agilent Cary 630 FTIR spectrometer equipped with an ATR MK-II Golden Gate Single Reflection system. UV–vis spectroscopy was performed on a Cary 50 Scan (Varian) UV–vis spectrophotometer with 1 cm quartz cells. CV and DPV experiments were performed in an IJ Cambria 660C potentiostat using a three-electrode cell. A GC electrode (3 mm diameter) from BAS was used as a working electrode, platinum wire as auxiliary, and SCE as the reference electrode. All cyclic voltammograms presented in this work were recorded under a nitrogen atmosphere. The complexes were dissolved in solvents containing the necessary amount of *n*-Bu_4_NPF_6_ (TBAH) as a supporting electrolyte to yield a 0.1 M ionic strength solution. All *E*_1/2_ values reported in this work were estimated from CV experiments as the average of the oxidative and reductive peak potentials (E_pa_+E_pc_)/2, or directly from DPV. Unless explicitly mentioned, the concentration of the complexes was approximately 1 mM. NMR spectroscopy was performed on Bruker DPX 300 and 400 MHz spectrometers. Samples were registered in CDCl_3_, CD_2_Cl_2_, or *d*_6_-DMSO. Elemental analyses were performed using a CHNS-O Elemental Analyzer EA-1108 from Fisons. ESI-MS experiments were performed on a Navigator LC/MS chromatograph from Thermo Quest Finnigan, using acetonitrile as the mobile phase. TEM studies were carried out using JEOL JEM 1210 at 120 kV. Scanning electron SEM and EDX analyses were done using the QUANTA FEI 200 FEG-ESEM device and also a FESEM Hitachi S4100. For metal content determination, a sequential inductively coupled plasma-atomic emission spectrometer (ICP-AES, Agilent 7500c, Agilent Technologies, Tokyo, Japan) was used. Prior to measurements, samples were digested with HCl/H_2_O/HNO_3_ at room temperature. XPS measurements were performed at room temperature with a SPECS PHOIBOS 150 hemispherical analyzer (SPECS GmbH, Berlin, Germany) in a base pressure of 5 × 10–10 mbar using monochromatic Al Kα radiation (1486.74 eV) as the excitation source operated at 300 W. The energy resolution as measured by the FWHM of the Ag 3d5/2 peak for a sputtered silver foil was 0.62 eV. GC measurements were taken with a Shimadzu GC-2010 gas chromatography apparatus equipped with an Astec CHIRALDEX G-TA column and a flame ionization detector (FID) detector.

### Crystallographic Data Collection and Structure Determination

The X-ray intensity data were measured on a Bruker D8 QUEST ECO three-circle diffractometer system equipped with a ceramic X-ray tube (Mo Kα, λ = 0.71076 Å) and a doubly curved silicon crystal Bruker Triumph monochromator, using the APEX3 software package.^53^ The frames were integrated with the Bruker SAINT.^54^ Data were corrected for absorption effects using the multi-scan method (SADABS).^55^ The structures were solved and refined using the Bruker SHELXTL.^56^

The crystallographic data as well as details of the structure solution and refinement procedures are reported in the Supporting Information. CCDC 2273724 (for trans-fac-**2**) contain the supplementary crystallographic data for this paper.

### Preparations

#### Synthesis of the Ligand and Complexes

The ligand bpea-pyrene was synthesized following a different method described in the literature.^38^

#### Synthesis of 1-[Bis(pyridine-2-ylmethyl)amino]methylpyrene, bpea-Pyrene

An aqueous solution (20 mL) of 2-picolyl chloride hydrochloride (4.1 g, 25 mmol) and 1-pyrenemethylamine hydrochloride (3.34 g, 12.5 mmol) was heated to 40–45 °C, while an aqueous solution (5 mL) of NaOH (2 g, 50 mmol) was quickly added. The resulting solution was stirred at the same temperature for 24 h. The reaction mixture was extracted with CHC1_3_ (3 × 30 mL). The extracts were dried over anhydrous MgSO_4_ and filtered, and a red solid was obtained via rotary evaporation of the solvent. Finally, the product was purified by means of a column of alumina eluting with chloroform. This procedure provided 5.52 g (75%) of pure ligand. IR (ν, cm^–1^): 3008, 2935, 2815, 1587, 1421, 843.

##### ^1^H NMR (400 MHz, CDCl_3_)

3.92 (s, 4H, H6), 4.40 (s, 2H, H7), 7.11 (dd, *J*_2–1_ = 1.2 Hz; J_2–3_ = 7.8 Hz;, 2H, H2), 7.49 (d, *J*_4–3_ = 7.6 Hz, 2H, H4), 7.60 (dd, *J*_3–4_ = 7.9 Hz; *J*_3–2_ = 7.8 Hz; 2H, H3), 7.98 (d, 1H, H12; t, 1H, He), 8.05 (d, 1H, Hb), 8.09 (d, 1H, Hk), 8.12 (d, 1H, Hl), 8.14 (d, 1H, Hf), 8.17 (d, 1H, Hh; d, 1H, Hd), 8.37 (d, 1H, Ha), 8.54 (d, 2H, H1).^13^C NMR (400 MHz, CDCl_3_): δ = 57.2 (C7), 60.6 (C6), 122.1 (C2), 123.3 (C4), 124.1 (Ca), 124.5 (Ck), 124.8 (*Cc*, Co), 124.9 (Ch, Cd), 125 (Cf), 125.8 (Ci), 125.9 (Ce), 127.1 (Cb), 128.1 (Cl), 129.9 (Cn), 130.8 (Cj), 131.3 (Cg, Cp), 132.6 (*Cm*), 136.4 C(3), 148,9 (C1), 159.6 (C5). *E*_1/2_ (CH_2_Cl_2_ + 0.1 M TBAH) = 1.11, 1.33, and 1.45 V vs SCE.

#### [RuCl_3_(bpea-pyrene)], **1**

A solution of RuCl_3_·2.5 H_2_O (0.609 g, 2.41 mmol) and bpea-pyrene (1 g, 2.41 mmol) in 200 mL of absolute MeOH was refluxed for 2 h under a N_2_ atmosphere. Afterward, a brown precipitate was filtered, washed with cold methanol and diethyl ether, and dried under vacuum. Yield: 0.850 g (57%).

Anal. found (calc.) for **2**: C, 55.78 (56.09); H, 3.91 (3.73); N, 6.54 (6.77). IR (ν, cm^–1^): 3029, 1737, 1606, 1438, 813.

#### Trans-fac-[RuCl(bpea-pyrene)(bpy)](PF_6_), Trans-fac-**2**

A sample of **1** (0.2 g, 0.32 mmol) and LiCl (0.03 g, 0.70 mmol) was dissolved in 25 mL of EtOH/H_2_O (9:1) under magnetic stirring. Then, NEt_3_ (0.08 mL, 0.70 mmol) was added and the reaction mixture was stirred at room temperature for 30 min. Afterward, 2,2′-bipyridine (0.049 g, 0.32 mmol) was added and the mixture was heated at reflux for 3 h. The hot solution was then filtered off and the volume was reduced in a rotary evaporator. After addition of 2 mL of a saturated aqueous solution of NH_4_PF_6_, a precipitate was formed, which was filtered off and washed with water. The solid obtained was purified by column chromatography (SiO_2_, CH_2_Cl_2_/MeOH, 98/2). Yield of trans-fac-**2**: 0.200 g (73%). Anal. found (calc.) for **2**·(Et)_2_O: C, 55.91(55.70); H, 4.38(4.43); N, 7.08 (7.56). IR (ν, cm^–1^): 3036, 1599,1439,835, 757. ESI-MS: [M-PF_6_]^+^ = 706.18.

Suitable crystals of trans-fac-**2** were grown as pale-yellow plates by diffusion of diethyl ether into a CHCl_3_ solution of the solid.

##### ^1^H NMR (CD_2_Cl_2_, 400 MHz)

δ = 3.68 (d, *J*_6b–6a_ = 16.0 Hz, 2H, H6b), 3.98 (s, 1H, H7), 4.48 (d, *J*_6a–6b_ = 16.0 Hz, 2H, H6a), 7.17 (d, *J*_4–3_ = 7.8 Hz, 2H, H4), 7.32 (d, *J*_8–9_ = 9.2 Hz, 2H, H8), 7.37 (t, *J*_2–1_= 5.6 Hz, *J*_2–3_ = 7.7 Hz 2H, H2), 7.63(td, *J*_10–11_ = Hz; *J*_10–9_ = 7.7 Hz; *J*_10–8_ = Hz, 2H, H10), 7.69(td, *J*_3–2_ = 7.7 Hz; *J*_3–4_ = 7.7 Hz; *J*_2–1_ = 1.6 Hz, 2H, H3), 7.92 (d, *J* = 7.9 Hz, 1H, Ha), 8.05(m, 2H, H9), 8.08(d, 1H, Hl),8.01–8.28 (m, 5H, H_pyrene_), 8.3 (d, J = 7.9 Hz, 1H, Hb), 8.45(d, 2H, H_k_), 8.50 (d, 2H, H11), 9.67 (d, J_1–2_ = 5.5 Hz, 2H, H1).

##### ^13^C NMR (CD_2_Cl_2_, 400 MHz)

δ = 153.8 (C1), 151.6(C11), 136.2 (C3), 135.3 (Cl), 129.4 (Ca), 129.1 (C9), 126.9–125.3 (Cd,e,f,h,i), 126.4 (Cb), 126.3 (C10), 125.5 (Ck), 124.4 (C2), 121 (C4), 120.5 (C8), 67 (C6a, C6b), 62.3 C(7). UV–vis (CH_2_Cl_2_) [λ_max_, nm (ε, M^–1^ cm^–1^)]: 243(23410), 278(14390), 346(11460), 388(3280), 512(1350). *E*_1/2_ (CH_2_Cl_2_ + 0.1 M TBAH) = 0.75 V vs SCE.

#### Trans-fac-[Ru(bpea-pyrene)(bpy)(OH_2_)](PF_6_)_2_, Trans**-**fac- **3**

##### Method **1**

A sample of fac-**2** (0.039 g 0.045 mmol) and AgNO_3_ (0.015 g, 0.092 mmol) was dissolved in 20 mL of a mixture water/acetone (3:1); the resulting solution was heated at reflux for 4 h in the absence of light. Then, the solution was filtered through Celite, and after reduction of the volume in a rotary evaporator, a saturated aqueous solution of NH_4_PF_6_ was added. The precipitate formed was filtered off and washed several times with cold water. The solid obtained in this manner was trans-fac-**3**. Yield: 0.034 g (70%). Anal. found (calc.) for 3·H_2_O: C, 46.81 (47.0); H, 3.3 (3.54); N, 6.85 (7.03). IR (ν, cm^–1^): 3280, 2900, 2820, 1600, 1420, 1300, 1220, 1100, 820, 780. ^1^H NMR (DMSO-*d*_6_, 400 MHz): δ = 3.85 (s, 2H,H7), 4.07 (d, *J*_6b-6a_ = 16.6 Hz, 2H, H6b), 4.92 (d, *J*_6a–6b_ = 16.5 Hz, 2H,H6a), 7.00 (d, *J*_a–b_ = 9.4 Hz, 1H, Ha), 7.43 (d, *J*_4–3_ = 7.8 Hz, 2H, H4), 7.52 (t, *J* = 6.7 Hz, 2H, H3), 7.97–7.84 (m, 4H, H2, H9), 8.09 (d, *J*_a–b_ = 9.4 Hz, 1H, Hb), 8.15 (t, *J* = 7.6 Hz, 1H, He), 8.4–8.2 (m, 6H, H_d,f,h,j,k,l_), 8.62 (d, *J*_8–9_ = 5.2 Hz, 2H, H8), 8.93 (d, *J*_11–10_ = 8.3 Hz, 2H, H11), 9.41 (d, *J*_1–2_= 6.3 Hz, 2H, H1). ^13^C NMR (DMSO-*d*_6_, 400 MHz): δ = 155(C1), 153(C8), 138(C10), 137(C2), 130(Cl), 128.5(Cb), 128 (C9), 127.5–123.5(Cd,f,h,I,k), 125.5 (Ce), 125 (C11), 124 (C3), 122 (C4), 120 (Ca), 65 (C6a, C6b), 57.5 C(7). UV–vis (phosphate buffer pH = 6.8) [λ_max_, nm (ε, M^–1^ cm^–1^)]: 242(19680), 278(14390), 348(11010), 462(1230). *E*_1/2_ (IV/II), phosphate buffer pH = 0.40 V vs SCE.

##### Method **2**

A sample of trans-fac-**2** (0.100 g, 0.115 mmol) was dissolved in 25 mL of a mixture water/acetone (3:1); the resulting solution was heated at 80 °C for 8 h in the absence of light. Then, the volume was reduced; after reduction of the volume in a rotary evaporator, a saturated aqueous solution of NH_4_PF_6_ was added. The precipitate formed was filtered off and washed several times with cold water. The solid obtained in this manner was the trans-fac-**3**. Yield: 0.042 mg (85%).

#### Functionalization of GO with Complex Trans-fac-**3**

##### GO@trans-fac-[Ru(bpea-pyrene)(bpy)(OH_2_)](PF_6_)_2_, GO@trans-fac-**3**

50 mg of GO suspended in 20 mL of water or CH_2_Cl_2_ was sonicated for 30 min. Then, 24 mg of the complex trans-fac-**3** was added. The suspension was stirred for 12 h at room temperature. The black solid was filtrated and washed with water or CH_2_Cl_2_. The corresponding solids were analyzed by ICP-MS in order to calculate the anchored complex. *E*_1/2_ (VI/II), phosphate buffer pH 6.8, 0.40 V vs SCE.

#### Functionalization of GC and GR with Complexes Trans-fac-**2** and Trans-fac-**3**

GC disk electrodes (3 mm of diameter) and GR electrodes (1 cm of height, 3.15 mm of diameter) were used as working electrodes in a three-electrode cell in the presence of the corresponding complexes trans-fac-**2** or trans-fac-**3** (0.5 or 1 mM) in degassed CH_2_Cl_2_ 0.1 M TBAH solution. Electropolymerization was performed through oxidation of the pyrene group using CV and recording different cycles at different rates (5–100 mV·s^–1^) between 0 and 1.4 V vs SCE. Afterward, the corresponding electrodes were rinsed with CH_2_Cl_2._

The amount of complex anchored was determined from the charge integrated under the oxidation peak in each case.

### Photocatalytic Oxidation Studies

#### Homogeneous Catalysis

A glass vessel containing a K_2_CO_3_ pH 7 aqueous solution (2.5 mL) together with the photocatalyst (0.49 mM), substrate (49 mM), and Na_2_S_2_O_8_ (98 mM) as sacrificial acceptor was stirred and exposed to continuous irradiation with a xenon lamp (150, Hamamatsu L8253), equipped with a 400–700 nm large band filter, at room temperature and atmospheric pressure, during different times. The resulting solution was extracted with dichloromethane (3 × 10 mL). The products were quantified by NMR and confirmed by gas chromatography analysis.

#### Heterogeneous Catalysis Using GO@trans-fac-**3**

Substrate (122 μmol) and Na_2_S_2_O_8_ (245 μmol) were dissolved in 2.5 mL of water (K_2_CO_3_ pH 7), together with the GO@trans-fac-**3** photocatalyst (0.12 μmol). The amount of heterogenized photocatalyst was calculated considering the functionalization of the GO support. The general photocatalytic oxidation experiments were all performed by exposing the resulting solution to continuous irradiation with a xenon lamp (150, Hamamatsu L8253), equipped with a 400–700 nm large band filter, at room temperature and atmospheric pressure, during different times. Afterward, the solution was centrifuged and the photocatalyst was separated by filtration. The reaction products were extracted with dichloromethane (4 × 10 mL). The combined organic phases were dried over sodium sulfate, and the solvent was evaporated under reduced pressure. The reaction products were quantified by means of NMR and confirmed by gas chromatography analysis.

#### Recycling Experiments

The reaction conditions indicated above were used in these experiments.

For every run, and after 6 h, the photocatalyst was recovered from the mixture of reaction by centrifugation, washed with water, and dried. Afterward, the solid was exposed to a new load of substrate under the same experimental conditions.

#### Heterogeneous Catalysis Using GR/Poly-trans-fac**-3**

Substrate (0.12 mmol) and Na_2_S_2_O_8_ (0.24 mmol) were dissolved in 2.5 mL of water (K_2_CO_3_ pH 7), and the modified graphite rod was added to the solution. The photocatalytic oxidation experiments were all performed by exposing the resulting solution to continuous irradiation with a xenon lamp (150, Hamamatsu L8253), equipped with a 400–700 nm large band filter, at room temperature and atmospheric pressure, during 6 h. Afterward, the photocatalyst was separated by filtration. The reaction products were extracted with dichloromethane (4 × 10 mL), evaporated, and quantified by means of NMR and confirmed by gas chromatography analysis (GC).

In the recycling experiment, after 6 h, the modified GR was recovered of the reaction by filtration, washed with water and dried, and exposed to a new load of substrate under the same experimental conditions.

## Supporting Information Available

Crystallographic information, spectroscopic and electrochemical characterization of the homogeneous and heterogeneous photocatalysts; and additional catalytic data (PDF)

## References

[ref1] Crisenza G. E. M.; Melchiorre P. Chemistry Glows Green with Photoredox Catalysis. Nat. Commun. 2020, 11, 803 10.1038/s41467-019-13887-8. 32029742 PMC7005190

[ref2] Renger G. Energy Transfer and Trapping in Photosystem II, in The Photosystems: Structure, Function and Molecular Biology. ed. Barber J., Elsevier Science Publishers: Amsterdam, The Netherlands,1992, 45–99.

[ref3] Turner J. A. Sustainable Hydrogen Production. Science 2004, 305, 972–974. 10.1126/science.1103197. 15310892

[ref4] Crabtree R. H. Energy Production and Storage– Inorganic Chemical Strategies for a Warming World, in Encyclopedia of Inorganic Chemistry, ed. John Wiley & Sons, Inc., 2nd ed, 2010; pp 73–87.

[ref5] Shaw M. H.; Twilton J.; MacMillan D. W. C. Photoredox Catalysis in Organic Chemistry. J. Org. Chem. 2016, 81, 6898–6926. 10.1021/acs.joc.6b01449. 27477076 PMC4994065

[ref6] Esswein A. J.; Nocera D. G. Hydrogen Production by Molecular Photocatalysis. Chem. Rev. 2007, 107, 4022–4043. 10.1021/cr050193e. 17927155

[ref7] Chen W.; Rein F. N.; Rocha R. C. Homogeneous Photocatalytic Oxidation of Alcohols by a Chromophore–Catalyst Dyad of Ruthenium Complexes. Angew. Chem., Int. Ed. 2009, 48, 9672–9675. 10.1002/anie.200904756. 19918829

[ref8] Chen W.; Rein F. N.; Scott B. L.; Rocha R. C. Catalytic photooxidation of Alcohols by an Unsymmetrical Tetra (pyridyl) pyrazine-Bridged Dinuclear Ru Complex. Chem. - Eur. J. 2011, 17, 5595–5604. 10.1002/chem.201002168. 21452180

[ref9] Farràs P.; Maji S.; Benet-Buchholz J.; Llobet A. Synthesis, Characterization, and Reactivity of Dyad Ruthenium-Based Molecules for Light-Driven Oxidation Catalysis. Chem. - Eur. J. 2013, 19, 7162–7172. 10.1002/chem.201204381. 23553647

[ref10] Lang X.; Zhao J.; Chen X. Cooperative Photoredox Catalysis. Chem. Soc. Rev. 2016, 45, 3026–3038. 10.1039/C5CS00659G. 27094803

[ref11] Hockin B. M.; Li C.; Robertson N.; Zysman-Colman E. Photoredox Catalysts Based on Earth-Abundant Metal Complexes. Catal. Sci. Technol. 2019, 9, 889–915. 10.1039/C8CY02336K.

[ref12] Guerrero I.; Kelemen Z.; Viñas C.; Romero I.; Teixidor F. Metallacarboranes as Photoredox Catalysts in Water. Chem. - Eur. J. 2020, 26, 5027–5036. 10.1002/chem.201905395. 31999000

[ref13] Guerrero I.; Saha A.; Xavier J.; Viñas C.; Romero I.; Teixidor F. Noncovalently Linked Metallacarboranes on Functionalized Magnetic Nanoparticles as Highly Efficient, Robust, and Reusable photocatalysts in Aqueous Medium. ACS Appl. Mater. Interfaces 2020, 12 (50), 56372–56384. 10.1021/acsami.0c17847. 33284598

[ref14] Guerrero I.; Viñas C.; Romero I.; Teixidor F. A stand-alone Cobalt bis(dicarbollide) Photoredox Catalyst Epoxidates Alkenes in Water at Extremely Low Catalyst Load. Green Chem. 2021, 23, 10123–10131. 10.1039/D1GC03119H.

[ref15] Huo J.; Zhang Y.-B.; Zou W.-Y.; Hu X.; Deng Q.; Chen D. Mini-review on an Engineering Approach Towards the Selection of Transition Metal Complex-based Catalysts for Photocatalytic H_2_ Production. Catal. Sci. Technol. 2019, 9, 2716–2727. 10.1039/C8CY02581A.

[ref16] Guerrero I.; Viñas C.; Fontrodona X.; Romero I.; Teixidor F. Aqueous Persistent Noncovalent Ion-Pair Cooperative Coupling in a Ruthenium Cobaltabis(dicarbollide) System as a Highly Efficient Photoredox Oxidation Catalyst. Inorg. Chem. 2021, 60, 8898–8907. 10.1021/acs.inorgchem.1c00751. 34096276 PMC8485323

[ref17] Zhu M.; Dong Y.; Xiao B.; Du Y.; Yang P.; Wang X. Enhanced Photocatalytic Hydrogen Evolution Performance Based on Ru-trisdicarboxybipyridine-Reduced Graphene Oxide Hybrid. J. Mater. Chem. 2012, 22, 23773–23779. 10.1039/c2jm35322a.

[ref18] Cano-Yelo H.; Deronzier A. Photo-oxidation of some Carbinols by the Ru(II) polypyridyl Complex-aryl Diazonium Salt System. Tetrahedron Lett. 1984, 25 (48), 5517–5520. 10.1016/S0040-4039(01)81614-2.

[ref19] Latorre-Sánchez M.; Lavorato C.; Puche M.; Fornés V.; Molinari R.; Garcia H. Visible-Light Photocatalytic Hydrogen Generation by Using Dye-Sensitized Graphene Oxide as a photocatalyst. Chem. - Eur. J. 2012, 18, 16774–16783. 10.1002/chem.201202372. 23111951

[ref20] Li J.; Wang D. Z. Visible-Light-Promoted Photoredox Syntheses of α,β-Epoxy Ketones from Styrenes and Benzaldehydes under Alkaline Conditions. Org. Lett. 2015, 17, 5260–5263. 10.1021/acs.orglett.5b02629. 26491880

[ref21] Rocha F.; Chen W.; Scott B.; Rocha R. Sunlight-Driven Dehydrogenative Oxidation Photocatalysis by a Mononuclear Complex Acting as both Chromophore and Catalyst. J. Braz. Chem. Soc. 2020, 31 (11), 2421–2429. 10.21577/0103-5053.20200161.

[ref22] Clerich E.; Affès S.; Anticó E.; Fontrodona X.; Teixidor F.; Romero I. Molecular and Supported Ruthenium Complexes as Photoredox Oxidation Catalysts in Water. Inorg. Chem. Front. 2022, 9, 5347–5359. 10.1039/D2QI01504H.

[ref23] Thompson M. S.; DeGiovani W. F.; Moyer B. A.; Meyer T. J. Novel Electrocatalytic Procedure for the Oxidation of Alcohols, Aldehydes, Cyclic Ketones, and C-H Bonds Adjacent to Olefinic or Aromatic Groups. J. Org. Chem. 1984, 49, 4972–4977. 10.1021/jo00199a043.

[ref24] Cherevatskaya M.; König B. Heterogeneous photocatalysts in Organic Synthesis. Russ. Chem. Rev. 2014, 83, 183–195. 10.1070/RC2014v083n03ABEH004427.

[ref25] Georgakilas V.; Otyepka M.; Bourlinos A. B.; Chandra V.; Kim N.; Kemp K. C.; Hobza P.; Zboril R.; Kim K. S. Functionalization of Graphene: Covalent and Non-Covalent Approaches, Derivatives and Applications. Chem. Rev. 2012, 112, 6156–6214. 10.1021/cr3000412. 23009634

[ref26] Kitanosono T.; Xu P.; Kobayashi S. Chiral Lewis Acids Integrated with Single-Walled Carbon Nanotubes for Asymmetric Catalysis in Water. Science 2018, 362 (6412), 311–315. 10.1126/science.aap7883. 30337405

[ref27] Sabater S.; Mata J. A.; Peris E. Catalyst Enhancement and Recyclability by Immobilization of Metal Complexes onto Graphene Surface by Noncovalent Interactions. ACS Catal. 2014, 4, 2038–2047. 10.1021/cs5003959.

[ref28] Wang J.; Kondrat S. A.; Wang Y.; Brett G. L.; Giles C.; Bartley J. K.; Lu L.; Liu Q.; Kiely C. J.; Hutchings G. J. Au–Pd Nanoparticles Dispersed on Composite Titania/Graphene Oxide-Supports as a Highly Active Oxidation Catalyst. ACS Catal. 2015, 5, 3575–3587. 10.1021/acscatal.5b00480.

[ref29] Li X.; Yu J.; Wageh S.; Al-Ghamdi A. A.; Xie J. Graphene in Photocatalysis: A Review. Small 2016, 12 (48), 6640–6696. 10.1002/smll.201600382. 27805773

[ref30] Le Goff A.; Gorgy K.; Holzinger M.; Haddad R.; Zimmerman M.; Cosnier S. Tris(bispyrene-bipyridine)iron(II): A Supramolecular Bridge for the Biofunctionalization of Carbon Nanotubes via p-Stacking and Pyrene/b-Cyclodextrin Host–Guest Interactions. Chem. - Eur. J. 2011, 17, 10216–1022. 10.1002/chem.201101283. 21818797

[ref31] Lalaoui N.; Reuillard B.; Philouze C.; Holzinger M.; Cosnier S.; Le Goff A. Osmium(II) Complexes Bearing Chelating N-Heterocyclic Carbene and Pyrene-Modified Ligands: Surface Electrochemistry and Electron Transfer Mediation of Oxygen Reduction by Multicopper Enzymes. Organometallics 2016, 35, 2987–2992. 10.1021/acs.organomet.6b00508.

[ref32] Georgakilas V.; Tiwari J. N.; Kemp K. C.; Perman J. A.; Bourlinos A. B.; Kim K. S.; Zboril R. Noncovalent Functionalization of Graphene and Graphene Oxide for Energy Materials, Biosensing, Catalytic and Biomedical Applications. Chem. Rev. 2016, 116, 5464–5519. 10.1021/acs.chemrev.5b00620. 27033639

[ref33] Feliz M.; Atienzar P.; Amela-Cortés M.; Dumait N.; Lemoine P.; Molard Y.; Cordier S. Supramolecular Anchoring of Octahedral Molybdenum Clusters onto Graphene and Their Synergies in Photocatalytic Water Reduction. Inorg. Chem. 2019, 58, 15443–15454. 10.1021/acs.inorgchem.9b02529. 31663340

[ref34] Zhang S.; Li B.; Wang X.; Zhao G.; Hu B.; Lu Z.; Wen T.; Chen J.; Wang X. Recent Developments of Two-dimensional Graphene-based Composites in Visible-Light Photocatalysis for Eliminating Persistent Organic Pollutants from Wastewater. Chem. Eng. J. 2020, 390, 124642 10.1016/j.cej.2020.124642.

[ref35] Arcas R.; Koshino Y.; Mas-Marzá E.; Tsuji R.; Masutani H.; Miura-Fujiwara E.; Haruyama Y.; Nakashima S.; Ito S.; Fabregat-Santiago F. Pencil Graphite Rods Decorated with Nickel and Nickel–Iron as Low-Cost Oxygen Evolution Reaction Electrodes. Sustainable Energy Fuels 2021, 5, 3929–3938. 10.1039/D1SE00351H.

[ref36] Kawde A.-N.; Baig N.; Sajid M. Graphite Pencil Electrodes as Electrochemical Sensors for Environmental Analysis: a Review of Features, Developments, and Applications. RSC Adv. 2016, 6, 91325–91340. 10.1039/C6RA17466C.

[ref37] Bachman J. C.; Kavian R.; Graham D. J.; Kim D. Y.; Noda S.; Nocera D. G.; Shao-Horn Y.; Lee S. W. Electrochemical Polymerization of Pyrene Derivatives on Functionalized Carbon Nanotubes for Pseudocapacitive Electrodes. Nat. Commun. 2015, 6, 7040 10.1038/ncomms8040. 25943905 PMC4432658

[ref38] Mullice L. A.; Laye R. H.; Harding L. P.; Buurma N. J.; Pope S. J. A. Rhenium Complexes of Chromophore-appended Dipicolylamineligands: Syntheses, Spectroscopic Properties, DNA Binding and X-Ray Crystal Structure. New J. Chem. 2008, 32, 2140–2149. 10.1039/b800999f.

[ref39] Serrano I.; López M. I.; Ferrer I.; Poater A.; Parella T.; Fontrodona X.; Solà M.; Llobet A.; Rodríguez M.; Romero I. New Ru(II) Complexes Containing Oxazoline Ligands as Epoxidation Catalysts. Influence of the Substituents on the Catalytic Performance. Inorg. Chem. 2011, 50, 6044–6054. 10.1021/ic200053f. 21650155

[ref40] Mola J.; Romero I.; Rodríguez M.; Bozoglian F.; Poater A.; Solà M.; Parella T.; Benet-Buchholz J.; Fontrodona X.; Llobet A. Mechanistic Insights into the Chemistry of Ru(II) Complexes Containing Cl and DMSO Ligands. Inorg. Chem. 2007, 46, 10707–10716. 10.1021/ic701421w. 18001116

[ref41] Romero I.; Rodríguez M.; Llobet A.; Collomb-Dunand-Sauthier M.-N.; Deronzier A.; Parella T.; Stoeckli-Evans H. Synthesis, Structure and Redox Properties of a New Ruthenium(II) Complex Containing the Flexible Tridentate Ligand N,N-bis(2-pyridylmethyl)ethylamine, cis-fac-Ru(bpea)^2+,^ and its Homologue Attached Covalently to a Polypyrrole Film. J. Chem. Soc., Dalton Trans. 2000, 1689–1694. 10.1039/a909511j.

[ref42] Rodríguez M.; Romero I.; Llobet A.; Deronzier A.; Biner M.; Parella T.; Stoeckli-Evans H. Synthesis, Structure, and Redox and Catalytic Properties of a New Family of Ruthenium Complexes Containing the Tridentate bpea Ligand. Inorg. Chem. 2001, 40, 4150–4156. 10.1021/ic010064q. 11487317

[ref43] Dunand-Sauthier M. N. C.; Deronzier A.; Romero I. Electrochemical generation of Binuclear Complexes [Mn_2_^III,III^(μ-O)(μ-OAc)_2_(bpea)_2_]^2+^ and [Mn_2_^IV,IV^(μ-O)_2_(μ-OAc)(bpea)_2_]^3+^ from the Mononuclear [Mn^II^(bpea)_2_]^2+^ Complex. J. Electroanal. Chem. 1997, 436, 219–225. 10.1016/S0022-0728(97)00314-8.

[ref44] Waltman R. J.; Diaz A. F.; Bargon J. The Electrochemical Oxidation and Polymerization of Polycyclic-Hydrocarbons. J. Electrochem. Soc. 1985, 132, 631–634. 10.1149/1.2113918.

[ref45] Costentin C. Electrochemical Approach to the Mechanistic Study of Proton-Coupled Electron Transfer. Chem. Rev. 2008, 108, 2145–2179. 10.1021/cr068065t. 18620365

[ref46] Ferrer Í.; Fontrodona X.; Roig A.; Rodríguez M.; Romero I. A Recoverable Ruthenium Aqua Complex Supported on Silica Particles: An Efficient Epoxidation Catalyst. Chem. - Eur. J. 2017, 23, 4096–4107. 10.1002/chem.201604463. 28075501

[ref47] Morgan D. J. Resolving Ruthenium: XPS Studies of Common Ruthenium Materials. Surf. Interface Anal. 2015, 47, 1072–1079. 10.1002/sia.5852.

[ref48] Manrique E.; Ferrer I.; Lu C.; Fontrodona X.; Rodríguez M.; Romero I. A Heterogeneous Ruthenium dmso Complex Supported onto Silica Particles as a Recyclable Catalyst for the Efficient Hydration of Nitriles in Aqueous Medium. Inorg. Chem. 2019, 58, 8460–8470. 10.1021/acs.inorgchem.9b00664. 31188583

[ref49] Wenjuan Y.; Le Goff A.; Spinelli N.; Holzinger M.; Diao G.-W.; Shan D.; Defrancq E.; Cosnier S. Electrogenerated Trisbipyridyl Ru(II)-/Nitrilotriacetic-Polypyrene Copolymer for the Easy Fabrication of Label-Free Photoelectrochemical Immunosensor and Aptasensor: Application to the Determination of Thrombin and Anti-Cholera Toxin Antibody. Biosens. Bioelectron 2013, 42, 556–562. 10.1016/j.bios.2012.11.013. 23261689

[ref50] Dakkach M.; Fontrodona X.; Parella T.; Atlamsani A.; Romero I.; Rodríguez M. Polypyrrole-Functionalized Ruthenium Carbene Catalysts as Efficient Heterogeneous Systems for Olefin Epoxidation. Dalton Trans. 2014, 43, 9916–9923. 10.1039/C4DT00698D. 24848551

[ref51] Si C.; Liu X.; Zhang T.; Xu J.; Li J.; Fu J.; Han Q. Constructing a photocatalyst for Selective Oxidation of Benzyl Alcohol to Benzaldehyde by Photo-Fenton-like Catalysis. Inorg. Chem. 2023, 62, 4210–4219. 10.1021/acs.inorgchem.2c04318. 36856314

[ref52] Xu J.; Luo L.; Xiao G.; Zhang Z.; Lin H.; Wang X.; Long J. Layered C_3_N_3_S_3_ Polymer/Graphene Hybrids as Metal-Free Catalysts for Selective Photocatalytic Oxidation of Benzylic Alcohols under Visible Light. ACS Catal. 2014, 4, 3302–3306. 10.1021/cs5006597.

[ref53] APEX3 V2018 1–0. *APX3 v*2018 1–0. *Bruker AXS.*, 2018.

[ref54] SAINT V8.38A. Bruker AXS. *SAINT V8.38A. Bruker AXS.*, 2017.

[ref55] SADABS-2016/2 - Bruker AXS Area Detector Scaling andAbsorption Correction. *SADABS*-2016/2 - *Bruker AXS área detector scaling and absorption correction*.

[ref56] Sheldrick G. M. A Short History of SHELX. Acta Crystallogr., Sect. A: Found. Crystallogr. 2008, 64 (1), 112–122. 10.1107/S0108767307043930. 18156677

