# Supplementary material for: Enhancing Photoredox Catalysis in Aqueous Environments: Ruthenium Aqua Complex Derivatization of Graphene Oxide and Graphite Rods for Efficient Visible-Light-Driven Hybrid Catalysts
Source: ACS Appl Mater Interfaces. 2023 Dec 19;16(1):507–19. doi: 10.1021/acsami.3c13156 (PMC10788860; doi:10.1021/acsami.3c13156)

SUPPORTING INFORMATION

Enhancing Photoredox Catalysis in Aqueous Environments: Ruthenium Aqua Complex Derivatization of Graphene Oxide and Graphite Rods for Efficient Visible Light-Driven Hybrid Catalysts

Syrine Affès,[a,b] *Akrivi Stamatelou,*[a] *Xavier Fontrodona,*[a] *Ahlem Kabadou,*[b] *Clara Viñas,*[c] *Francesc Teixidor**[c] *and Isabel Romero**[a]

[a] Departament de Química and Serveis Tècnics de Recerca, Universitat de Girona, C/ M. Aurèlia Campmany, 69, E-17003 Girona, Spain.
[b] Laboratoire des Sciences des Matériaux et d'Environnement, Faculté des Sciences, Université de Sfax, 3000 Sfax, Tunisie.
[c] Institut de Ciencia de Materials de Barcelona, ICMAB-CSIC, Campus UAB, E-08193 Bellaterra, Spain.

Corresponding authors

*****E-mail for I.R.: marisa.romero@udg.edu; *****E-mail for F.T.: teixidor@icmab.es

Structural characterization

Table S1. Crystallographic data for the X-ray diffraction of the *trans-fac*-**2** complex.

Table S2. Selected bond lengths (Å) and angles (°) for *trans-fac*-**2** complex.

Scheme S1. Possible isomers for *trans-fac*-**2** and **3** complexes.

Figure S1. Hydrogen bond interactions for *trans-fac*-**2**. Circles in grey correspond to C atoms; in yellow to H atoms, in greenish yellow to F atoms, in orange to P atoms, in green to Cl, in blue to N atoms and, in greenish blue to Ru atom.

Figure S2. Packing arrangement for *trans-fac*-**2,** a) along axis *a* and b) along axis*;* $CHCl_3$ molecules of the solvent molecules as well as the ones of the $[PF_6]^-$ anions are omitted.

Figure S3. IR spectra of a) *bpea-pyrene*; b) complex **1**; c) complex *trans-fac*-**2** and complex *trans-fac*-**3.**

Figure S4. NMR spectra of *bpea-pyrene* (400 MHz, $CDCl_3$): a) ^{1}H-NMR; b) ^{13}C{^{1}H}-NMR; c) DEPT; d) COSY; e) NOESY; f) TOCSY; g)^{1}H-^{13}C HSQC.

Figure S5. NMR spectra of *trans-fac*-**2**, 400 MHz, CD_2Cl_2: a) ^{1}H-NMR; b) ^{13}C{^{1}H}-NMR; c) COSY; d) NOESY; e) HSQC.

Figure S6. NMR spectra of *trans-fac*-**3**, 400 MHz, d_6-DMSO: a) ^{1}H-NMR; b) COSY; c) ROESY; d) HSQC; e) HSQC and HMBC.

Figure S7. UV-*vis* spectra of *trans-fac*-**2** (blue line), *trans-fac*-**3** (orange line) in CH_2Cl_2.

Figure S8. CV of a) ligand *bpea-pyrene* in CH_2Cl_2 containing 0.1 M [*n*-Bu_4][NPF_6] (TBAH) *vs* SCE.

Figure S9. a) Differential Pulse Voltammetry (DPV) of a) *trans-fac*-**2** in CH_2Cl_2 containing 0.1 M [*n*-Bu_4N][PF_6] (TBAH) *vs* SCE and, b) *trans-fac*-**3** in phosphate buffer (pH=6.8) *vs* SCE.

Figure S10. Pourbaix diagram for *trans-fac*-**3** in the range pH=2-9.

Figure S 11. UV-*vis* spectrum of *trans-fac*-**3** (1mM) in water (blue line); UV-visible of the initial solution after 1 h in contact with GO (orange line); after 24 h (green line).

Figure S12. a) TEM image of GO@*trans-fac*-**3**; b) SEM images of GO support and b) GO@*trans-fac*-**3**.

Figure S13. EDX spectra of GO@*trans-fac*-**3**.

Figure S14. a) XPS spectra of GO@*trans-fac*-**3**; Magnification of the XPS spectrum of GO@*trans-fac*-**3** in the b) O1s region and c) N1s region

Figure S15. UV-*vis* spectra of *trans-fac*-**3** (blue line) (0.1mM), GO@*trans-fac*-**3** (orange line) and GO (green line) in CH_2Cl_2.

Figure S16. a) CV for the electropolymerization of *trans-fac*-**2** on GC electrode and b) CV of the polymerized GC/poly-*trans-fac*-**2** modified electrode in a blank solution, CH_2Cl_2 + 0.1 M TBAH.

Figure S17. a) CVs of GC electrodes functionalized with 1mM of *trans-fac*-**2** in CH_2Cl_2+0.1MTBAH; b) Plot of cathodic and anodic peaks current as function of the scan rate for GC/*trans-fac*-**2**.

Figure S18. CV of the polymerized GC/poly-*trans*-*fac*-**3** modified electrode in a blank solution, CH_2Cl+ 0.1 M TBAH.

Figure S19. CV for the electropolymerization of *trans*-*fac*-**2** on graphite rods.

Figure S20. SEM images of GR/poly-*trans*-*fac*-**3**, a) using a SE detector and b) using a BSE detector.

Figure S21. Plot of yield as a function of time for the photoredox catalysis of 1-phenylethanol using *trans*-*fac*-**3** as photocatalyst. Conditions: *trans*-*fac*-**3** (0,49 mM), substrate (49 mM), $Na_2S_2O_8$ (98 mM), 2.5ml water (K_2CO_3, pH=7), light irradiation using a lamp with λ= 400-700 nm.

Figure S22. TEM images of GO@*trans*-*fac*-**3** a) before the photooxidation of 4-methylbenzyl alcohol and b) after five reuses.

Figure S23. SEM images of GO@*trans*-*fac*-**3** in the photooxidation of 4-methylbenzyl alcohol after five reuses.

Structural characterization

The Ru metal centre is coordinated by one N-tridentate *bpea-pyrene* ligand and by a bidentate *bpy* ligand, respectively; being the sixth coordination site occupied by the chlorido ligand, adopting an octahedrally distorted type of coordination. The *bpea-pyrene* ligand is coordinated in a facial fashion with the aliphatic nitrogen, N_{al} (N22) situated *trans* to the chorido ligand and the aromatic nitrogens, N_{ar} (N15, N29) *trans* to the pyridyl nitrogen atoms of the *bpy* ligand (N3, N14), which corroborates the formation of the *trans-fac*-**2** isomer in solid state. All bond distances and angles are within the expected values for this type of compounds.[1,2] It is noteworthy that the distinct electronic nature of the nitrogen atoms of the *bpea-pyrene* ligand, when coordinated to the Ru center, result in varying Ru-N bond lengths. Specifically, the shortest distances are observed for the Ru-N_{ar} bonds (Ru-N15, 2.069 Å and Ru-N29, 2.054 Å) with regard to the Ru-N_{al} bond, Ru-N22, 2.119 Å). Weak hydrogen bond interactions can be observed between hydrogens of the pyridine rings of the *bpea-pyrene* ligand and the chlorido ligand (H16-Cl2, 2.738 Å and H28-Cl2, 2.840 Å) (Figure S1). Others hydrogen bonds have been observed between $[PF_6]^-$ anions and hydrogen atoms from the *bpy* ligand, (F3R-H5, 2.408 Å) and from the *bpea-pyrene* (F2S-H25, 2.752 Å) (see Figure S1). Figure S2 shows the packing of *trans-fac*-**2** along the axis *a* and *b*. The tridimensional arrangement of molecules in *trans-fac*-**2** displays a network in the *b* axis direction of intermolecular π-stacking interactions involving two-pyrene substituent and two *bpea-pyrene* ligands of two neighbouring molecules, (interplanar distance 3.49 Å).

---

(1) Romero, I.; Rodríguez, M.; Llobet, A.; Collomb-Dunand-Sauthier, M-N.; Deronzier, A.; Parella T.; Stoeckli-Evans, H. Synthesis, Structure and Redox Properties of a New Ruthenium(II) Complex Containing the Flexible Tridentate Ligand *N,N*-bis(2-pyridylmethyl)ethylamine, *cis-fac*-Ru(bpea)$^{2+,}$ and its Homologue Attached Covalently to a Polypyrrole Film. *J. Chem. Soc., Dalton Trans.* **2000**, 1689-1694.

(2) Rodríguez, M.; Romero, I.; Llobet, A.; Deronzier, A.; Biner, M.; Parella T.; Stoeckli-Evans, H. Synthesis, Structure, and Redox and Catalytic Properties of a New Family of Ruthenium Complexes Containing the Tridentate bpea Ligand. *Inorg. Chem.* **2001**, *40,* 4150-4156.

Table S1. Crystallographic data for the X-ray diffraction of *trans-fac*-**2** complex.

	Trans-fac-**2**
Empirical formula	$C_{41}H_{35}Cl_7F_6N_5OPRu$
Formula weight	1107.93 g/mol
Crystal system	triclinic
Space group	P-1
a[Å]	9.912(10)
b[Å]	11.808(12)
c[Å]	18.535(18)
α[º]	86.02(3)
β[º]	85.57(3)
γ[º]	82.13(3)
V [$Å^3$]	2139.(4)
Formula Units/ cell	2
Temp. [K]	100(2)
ρ_{calc}, [g/cm^{-3}]	1.720
μ[mm^{-1}]	0.910
Final R indices, [I>2σ(I)]	R_1 = 0.0519<br>wR_2 = 0.1345
R indices [all data]	R_1 = 0.0653<br>wR_2 = 0.1433

Table S2. Selected bond lengths (Å) and angles (°) for *trans*-*fac*-**2** complex.

	trans-**2**
Ru(1)-N(3)	2.050(3)
Ru(1)-N(15)	2.061(3)
Ru(1)-N(14)	2.056(4)
Ru(1)-N(22)	2.108(4)
Ru(1)-N(29)	2.065(4)
Ru(1)-Cl(2)	2.408(2)
N(3)-Ru(1)-N(14)	79.17(15)
N(14)-Ru(1)-N(15)	92.31(14)
N(15)-Ru(1)-N(29)	95.86(14)
N(29)-Ru(1)-N(3)	92.51(14)
N(3)-Ru(1)-N(15)	171.25(12)
N(14)-Ru(1)-N(29)	171.06(13)
N(3)-Ru(1)-Cl(2)	86.60(11)
N(14)-Ru(1)-Cl(2)	87.14(10)
N(3)-Ru(1)-N(22)	97.81(13)
N(14)-Ru(1)-N(22)	98.62(12)
N(15)-Ru(1)-N(22)	81.34(13)
N(29)-Ru(1)-N(22)	79.10(13)
N(22)-Ru(1)-Cl(2)	173.30(8)
N(15)-Ru(1)-Cl(2)	95.06(11)
N(29)-Ru(1)-Cl(2)	95.72(10)

Scheme S1. Possible isomers for *trans-fac*-**2** and **3**. N-N is bipyridine ligand and X is Cl^- or H_2O.

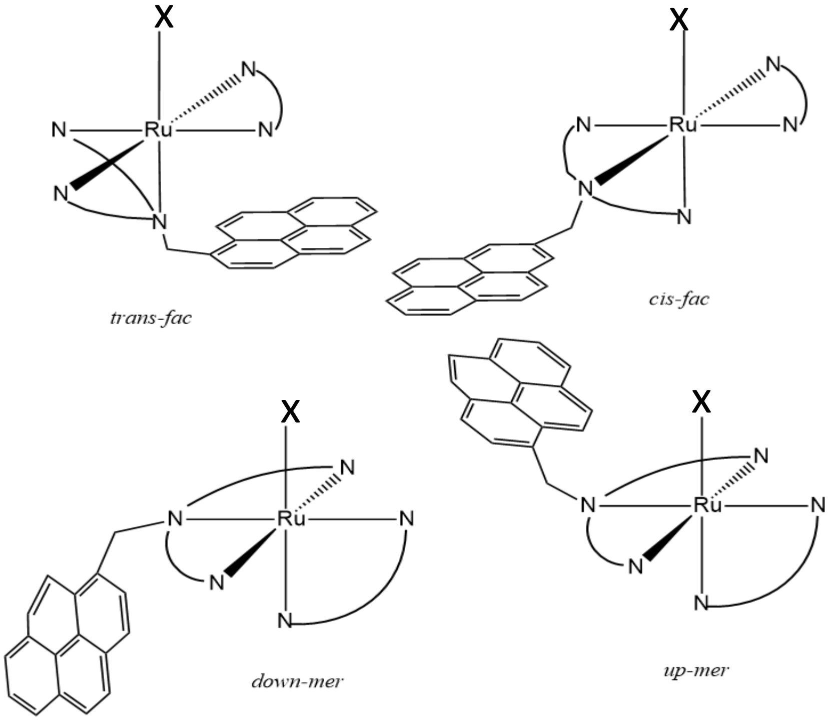

Meridional coordination of the *bpea-pyrene* ligand leads to the formation of two possible stereoisomers (*up-mer* and *down-mer*) where the difference between both stereoisomers would be the orientation of the pyrene group in the complex. In both cases, the protons in the aromatic rings of the bipyridine ligand would be magnetically non-equivalent. However, when the ligand is coordinated in facial fashion, the monodentate ligands (Cl^- or H_2O) can be located *trans* or *cis* with regard to the aliphatic nitrogen (N_{al}) of the ligand, and other different stereoisomers could be obtained, the *trans-fac* and *cis-fac*. Then the nomenclature *trans*- or *cis*- refers to the relative position of the monodentate ligands, Cl or H_2O. The *cis-fac* arrangement could lead to two possible stereoisomers, the *cis-fac-Λ* and the *cis-fac*-Δ and the pyridyl ring become non-equivalent. For the *trans-fac* isomer, the molecule would have a plane that would contain the Ru atom, the monodentate ligand (Cl^- or H_2O) and the aliphatic nitrogen, being equivalent all the pyridylic rings.

Figure S1. Hydrogen bond interactions for *trans*-*fac*-**2**. Circles in grey correspond to C atoms; in yellow to H atoms, in greenish yellow to F atoms, in orange to P atoms, in green to Cl, in blue to N atoms and, in greenish blue to Ru atom.

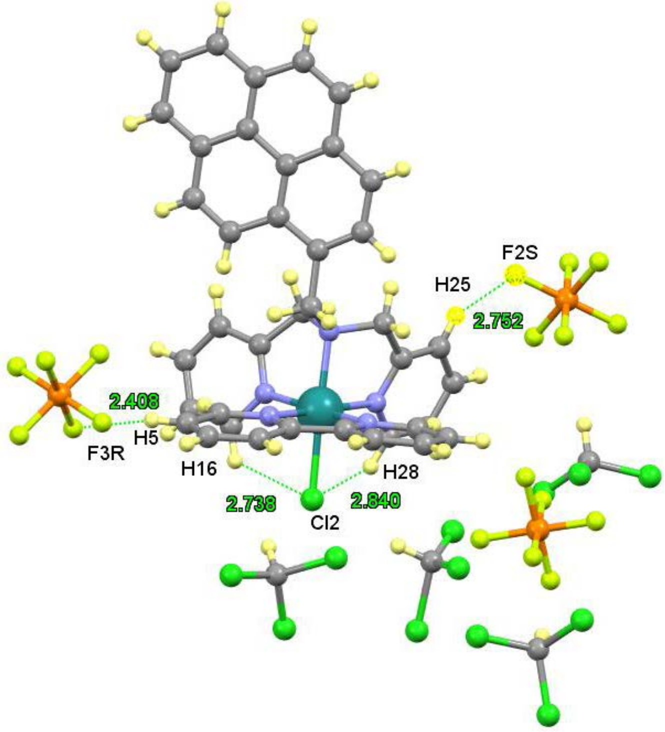

Figure S2. Packing arrangement for *trans-fac*-**2,** a) along axis *a* and b) along axis *b;* $CHCl_3$ molecules of the solvent molecules as well as the ones of the $[PF_6]^-$ anions are omitted.

a)

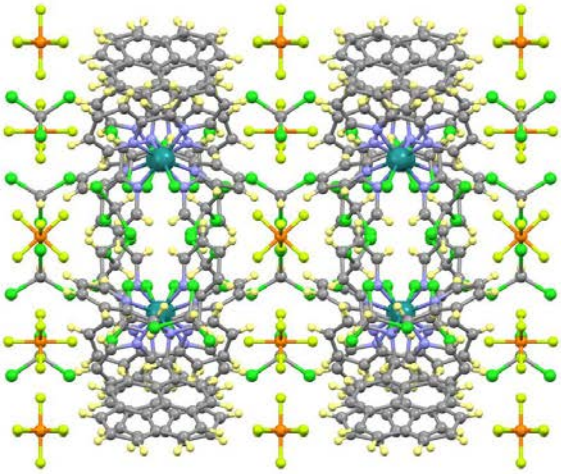

b)

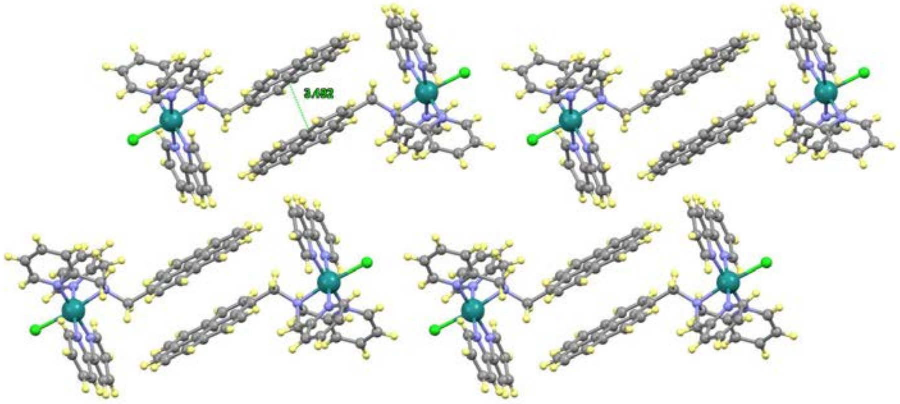

Figure S3. IR spectra of a) *bpea-pyrene*; b) complex **1**; c) complex *trans-fac*-**2** (blue) and complex *trans-fac*-**3** (orange).

a)

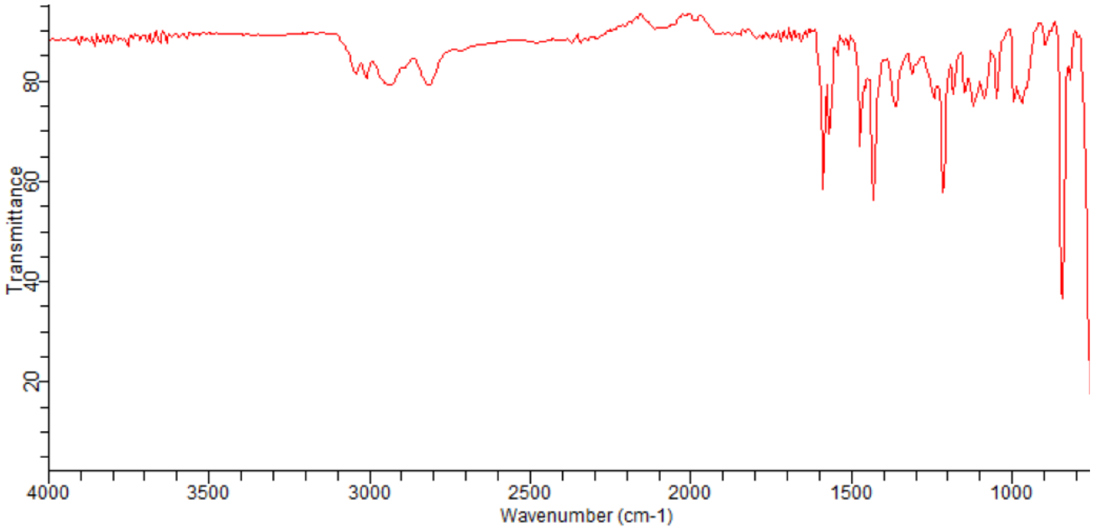

b)

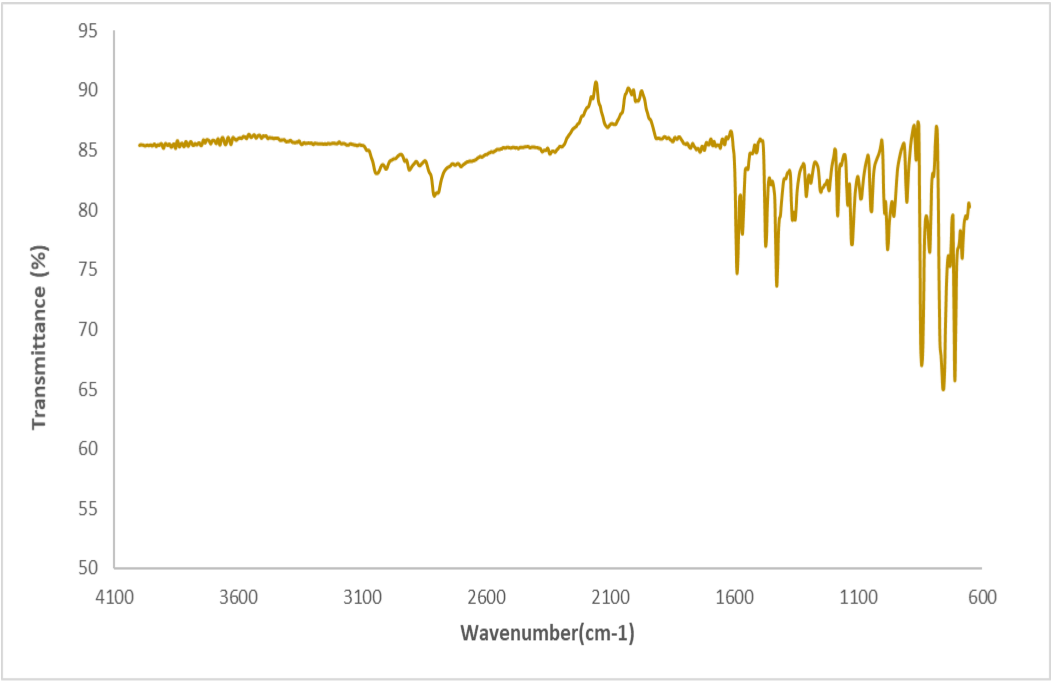

c)

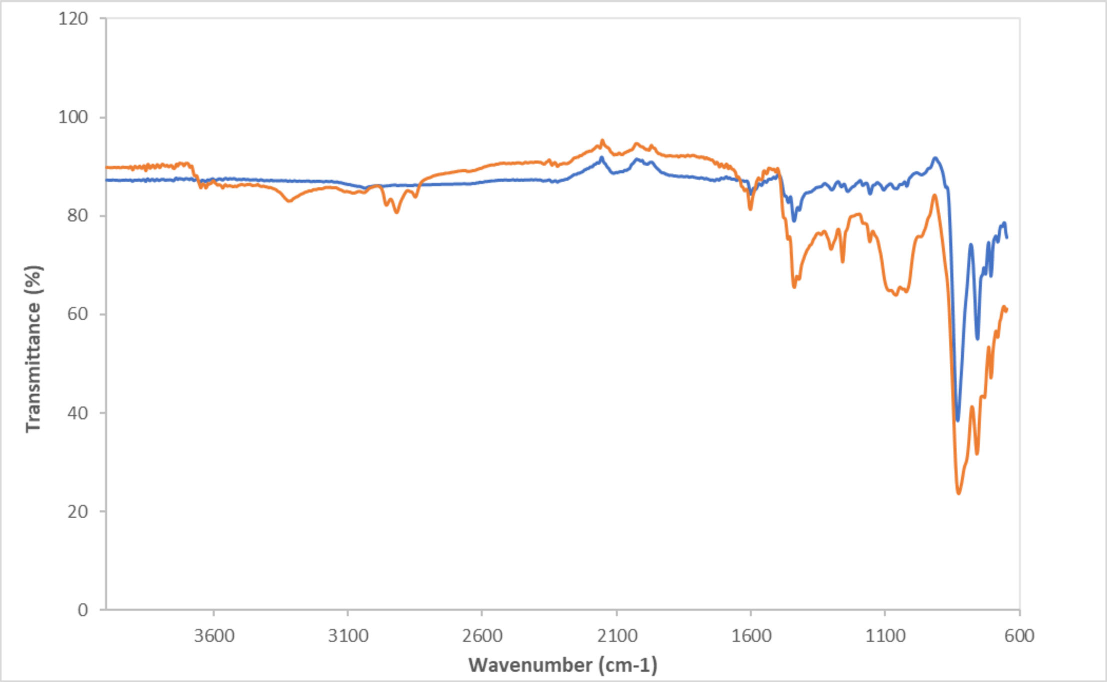

120
100
80
60
40
20
0
Transmittance (%)
3600
3100
2600
2100
1600
1100
600
Wavenumber (cm-1)

Figure S4. NMR spectra of *bpea-pyrene* (400 MHz, $CDCl_3$): a) ^{1}H-NMR; b) ^{13}C{^{1}H}-NMR; c) DEPT; d) COSY; e) NOESY; f) TOCSY; g)^{1}H-^{13}C HSQC.

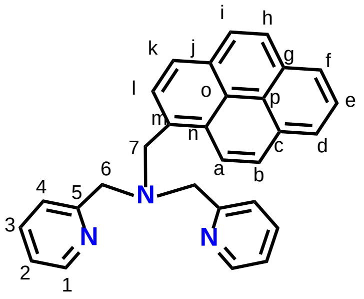

a)

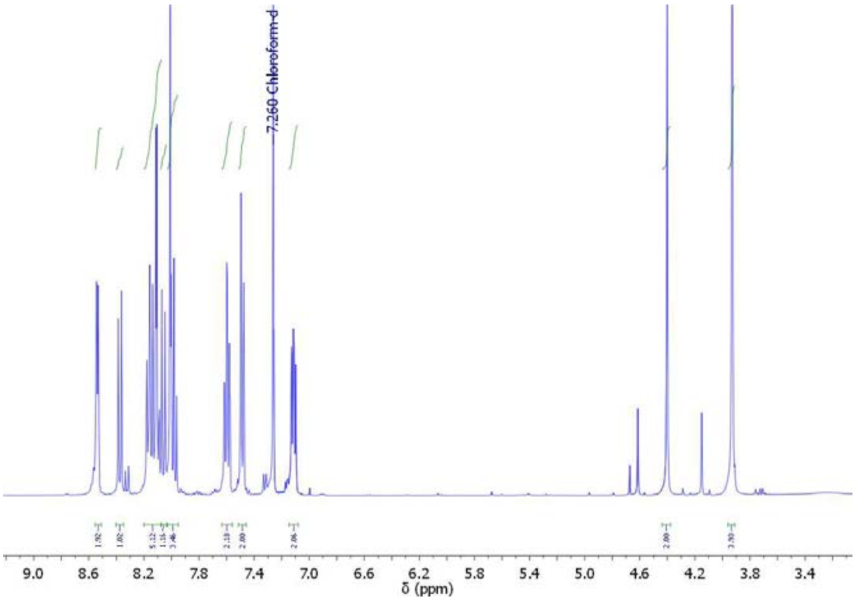

b)

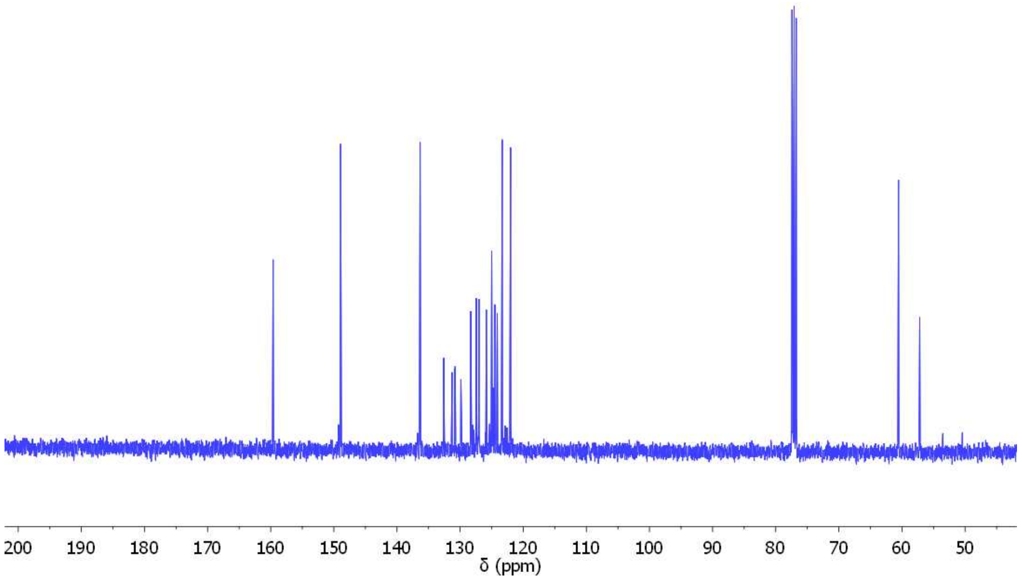

c)

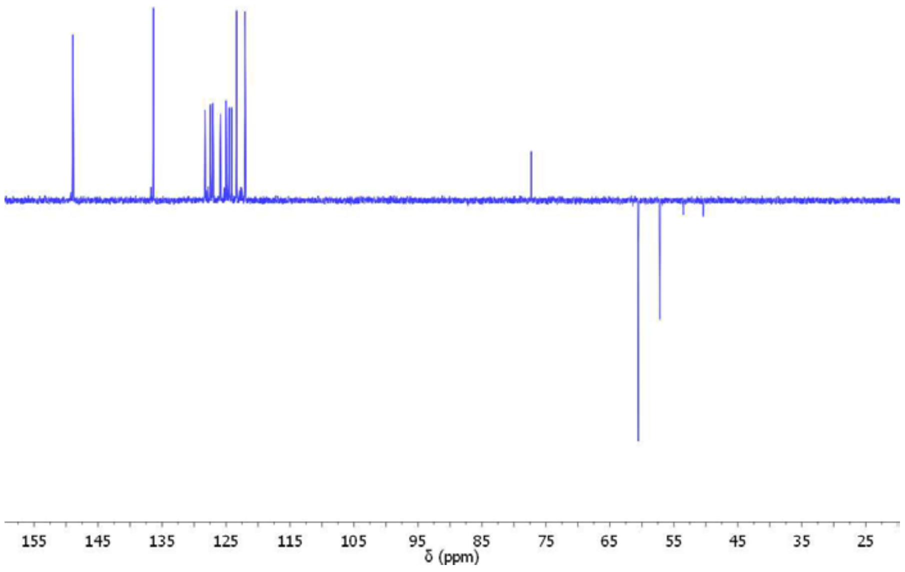

155 145 135 125 115 105 95 85 75 65 55 45 35 25
δ (ppm)

d)

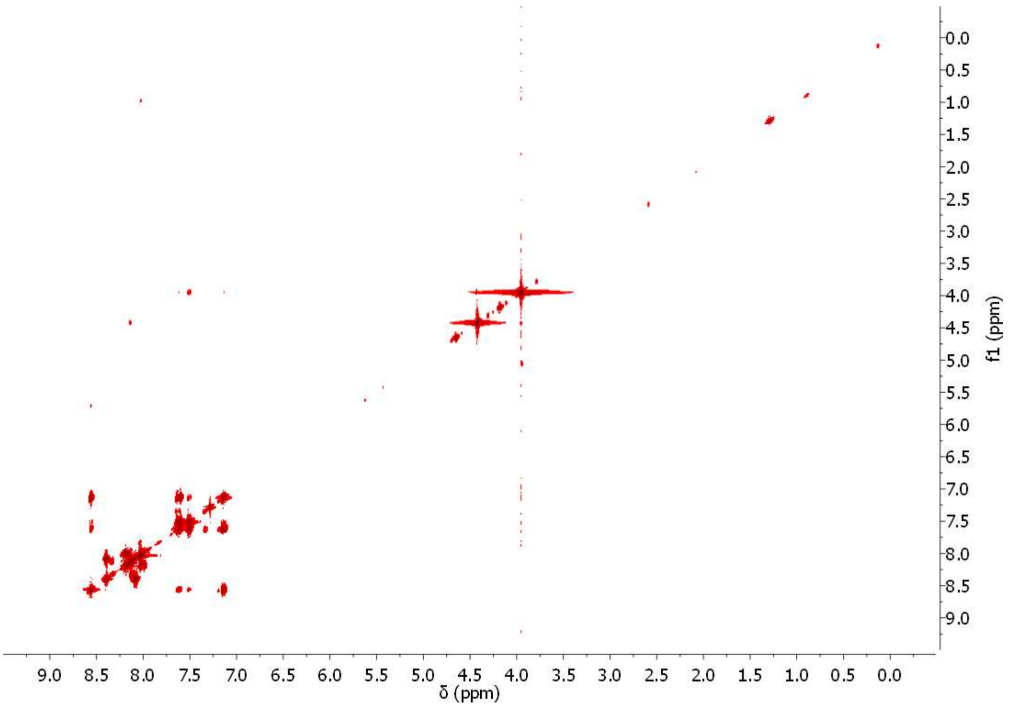

9.0 8.5 8.0 7.5 7.0 6.5 6.0 5.5 5.0 4.5 4.0 3.5 3.0 2.5 2.0 1.5 1.0 0.5 0.0
δ (ppm)
f1 (ppm)

e)

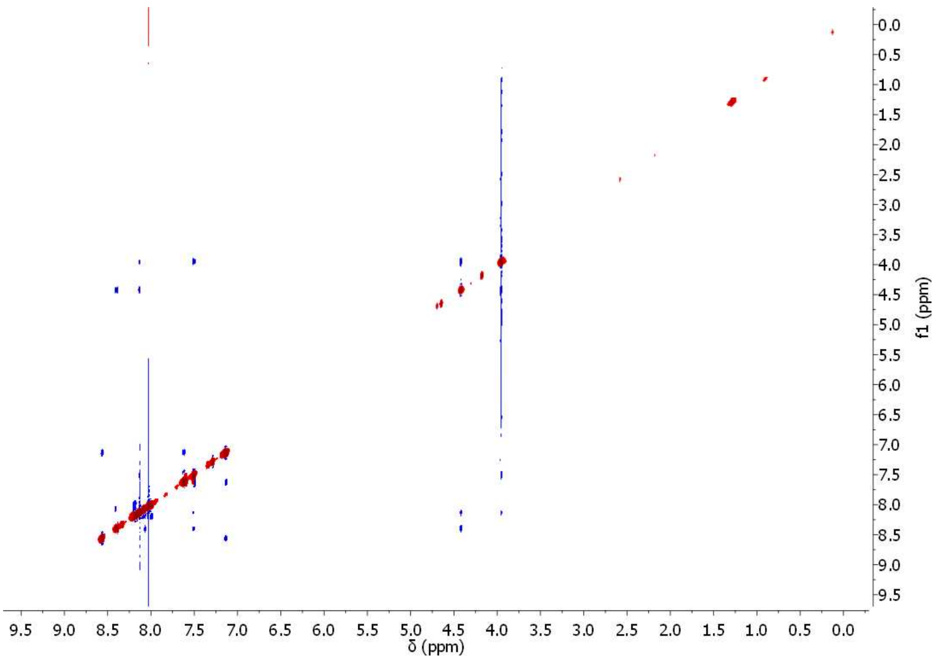

f)

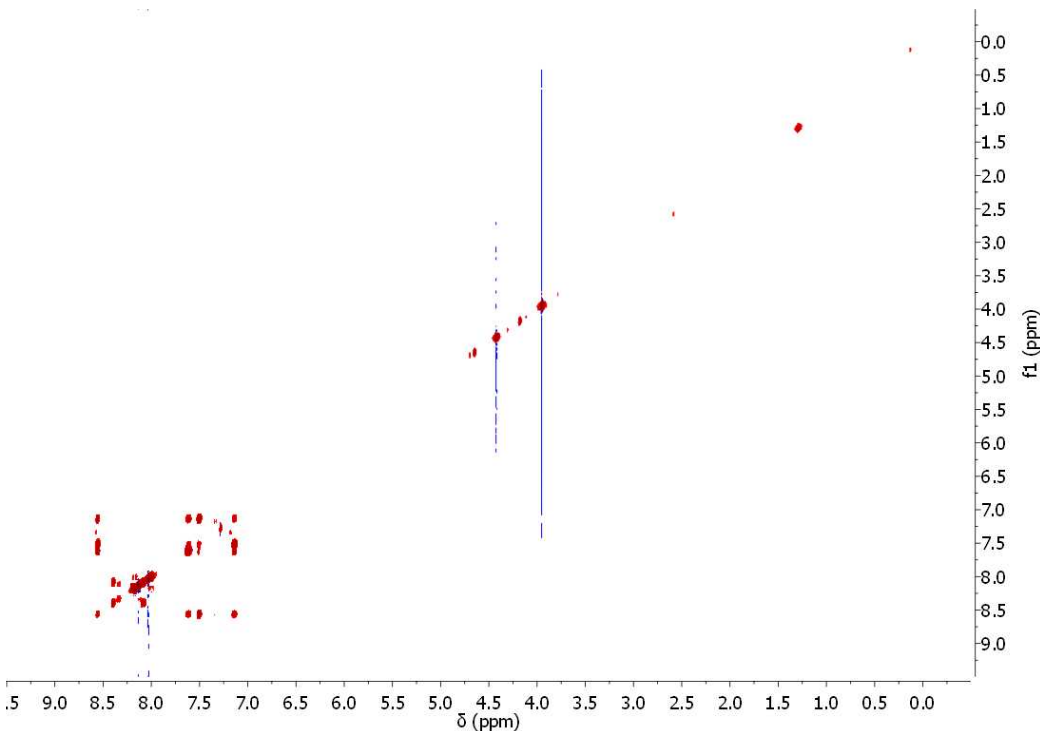

g)

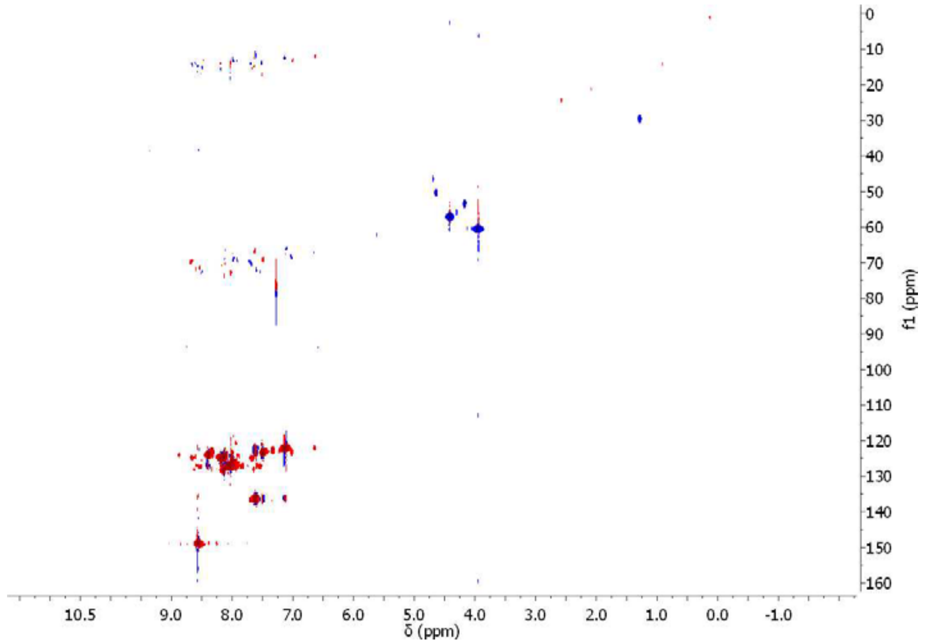

0
10
20
30
40
50
60
70
80
90
100
110
120
130
140
150
160
f1 (ppm)
10.5
9.0
8.0
7.0
6.0
5.0
4.0
3.0
2.0
1.0
0.0
-1.0
δ (ppm)

Figure S5. NMR spectra of *trans*-*fac*-**2**, 400 MHz, CD_2Cl_2: a) ^{1}H-NMR; b) ^{13}C{^{1}H}-NMR; c) COSY; d) NOESY; e) HSQC.

a)

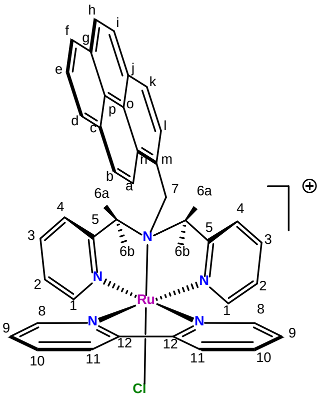

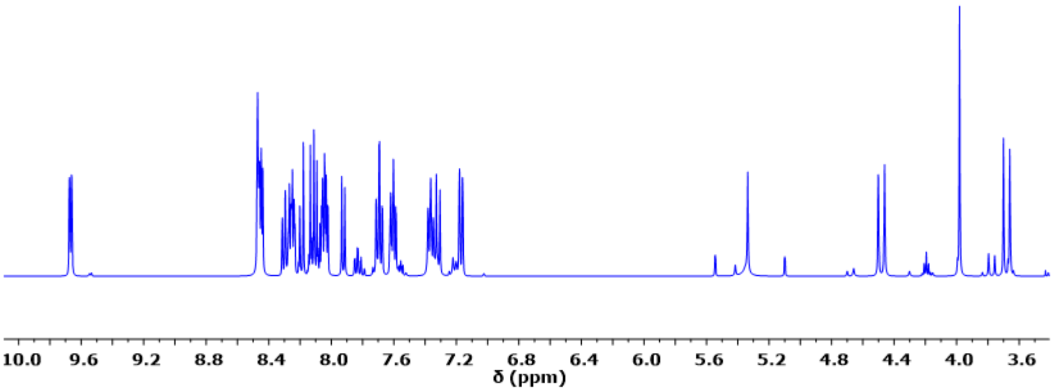

b)

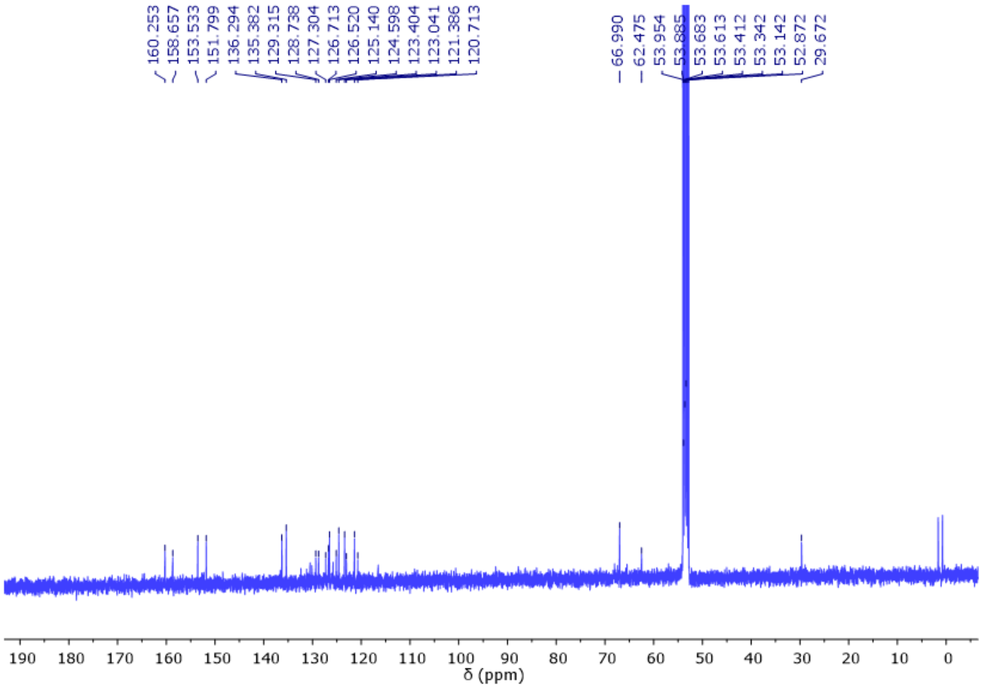

c)

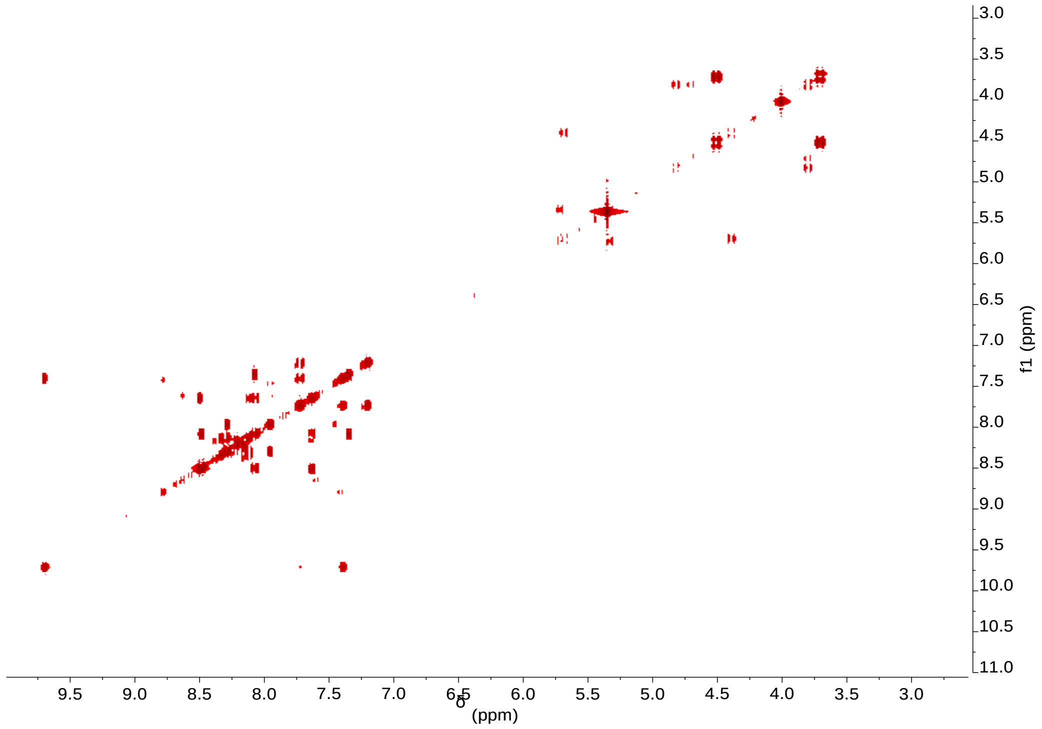

3.0
3.5
4.0
4.5
5.0
5.5
6.0
6.5
7.0
7.5
8.0
8.5
9.0
9.5
10.0
10.5
11.0
f1 (ppm)
9.5 9.0 8.5 8.0 7.5 7.0 6.5 6.0 5.5 5.0 4.5 4.0 3.5 3.0
δ (ppm)

d)

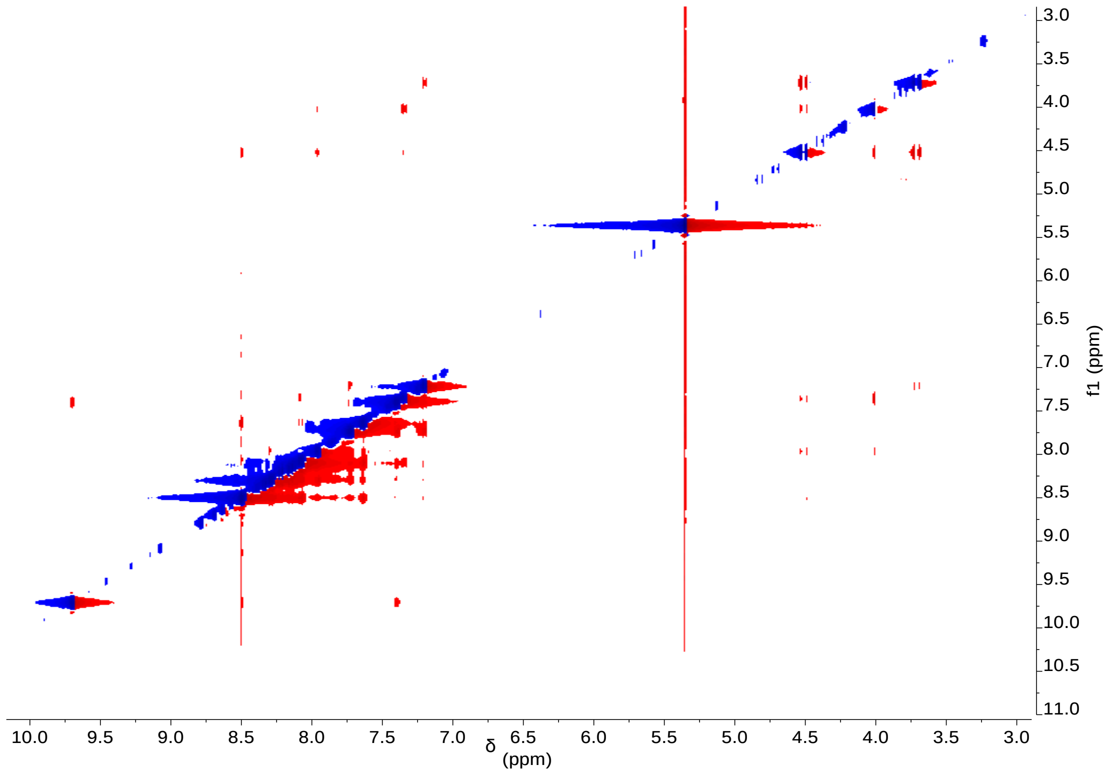

3.0
3.5
4.0
4.5
5.0
5.5
6.0
6.5
7.0
7.5
8.0
8.5
9.0
9.5
10.0
10.5
11.0
f1 (ppm)
10.0 9.5 9.0 8.5 8.0 7.5 7.0 6.5 6.0 5.5 5.0 4.5 4.0 3.5 3.0
δ (ppm)

e)

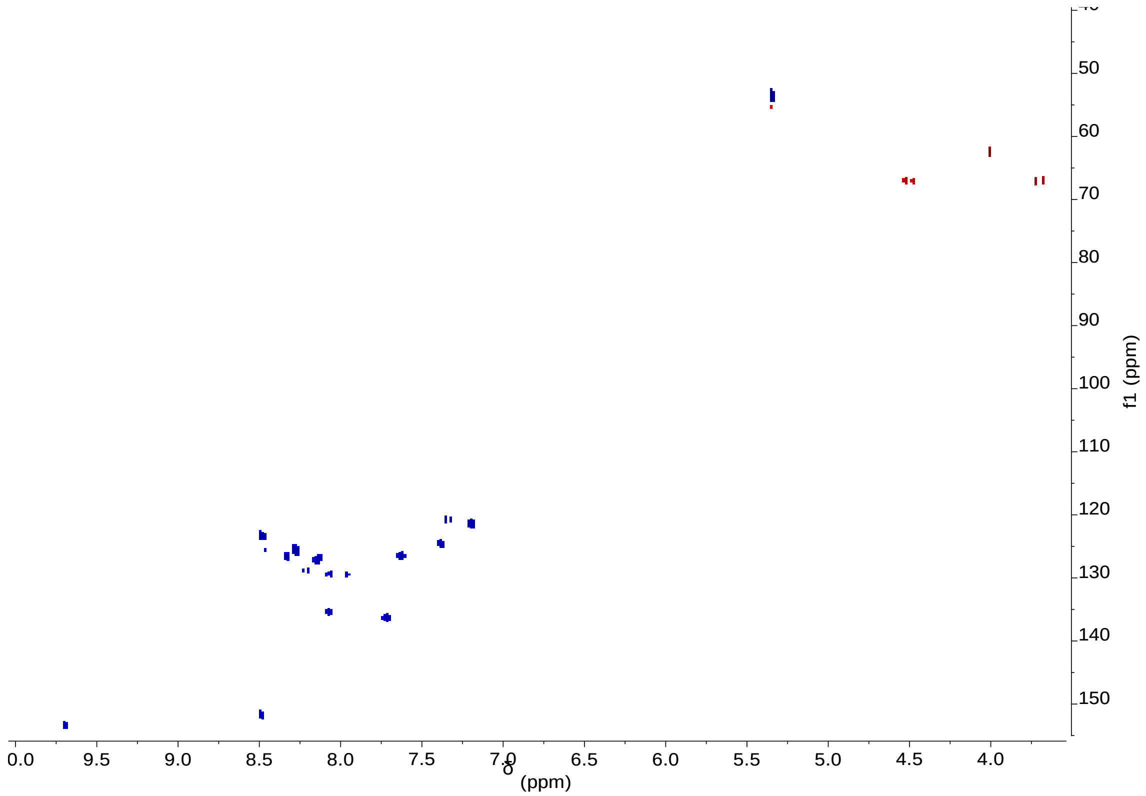

50
60
70
80
90
100
110
120
130
140
150
f1 (ppm)
0.0
9.5
9.0
8.5
8.0
7.5
7.0
6.5
6.0
5.5
5.0
4.5
4.0
δ (ppm)

Figure S6. NMR spectra of *trans-fac*-**3**, 400 MHz, d_6-DMSO: a) ^{1}H-NMR; b) COSY; c) ROESY; d) HSQC; e) HSQC and HMBC.

a)

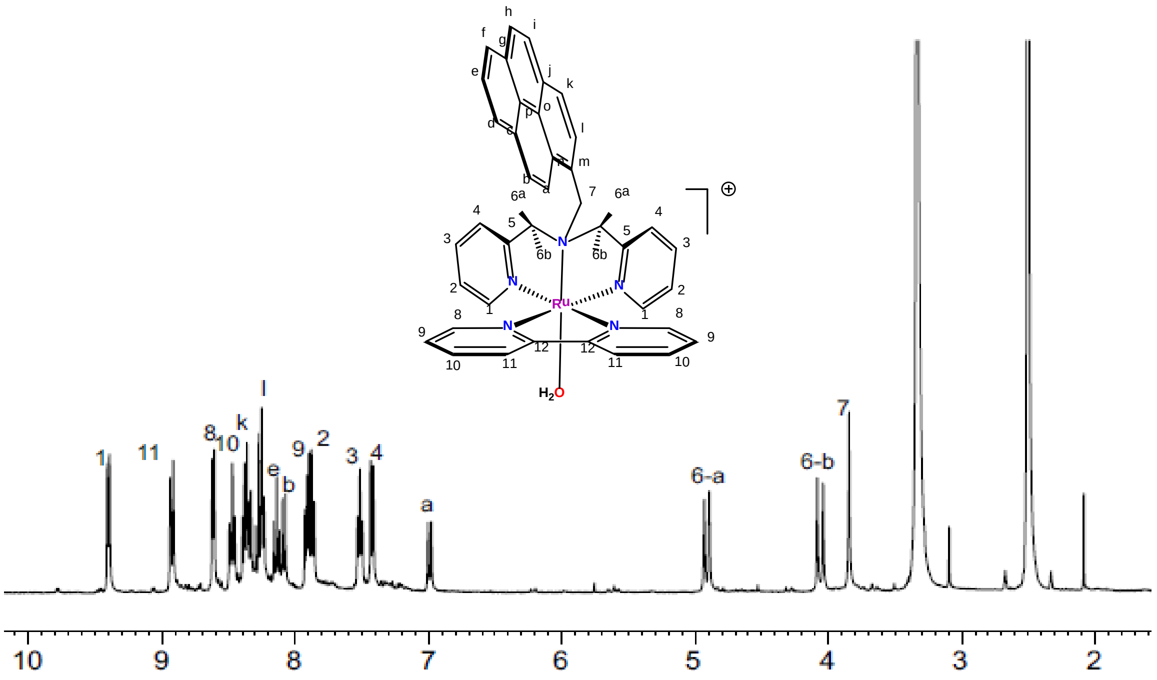

b)

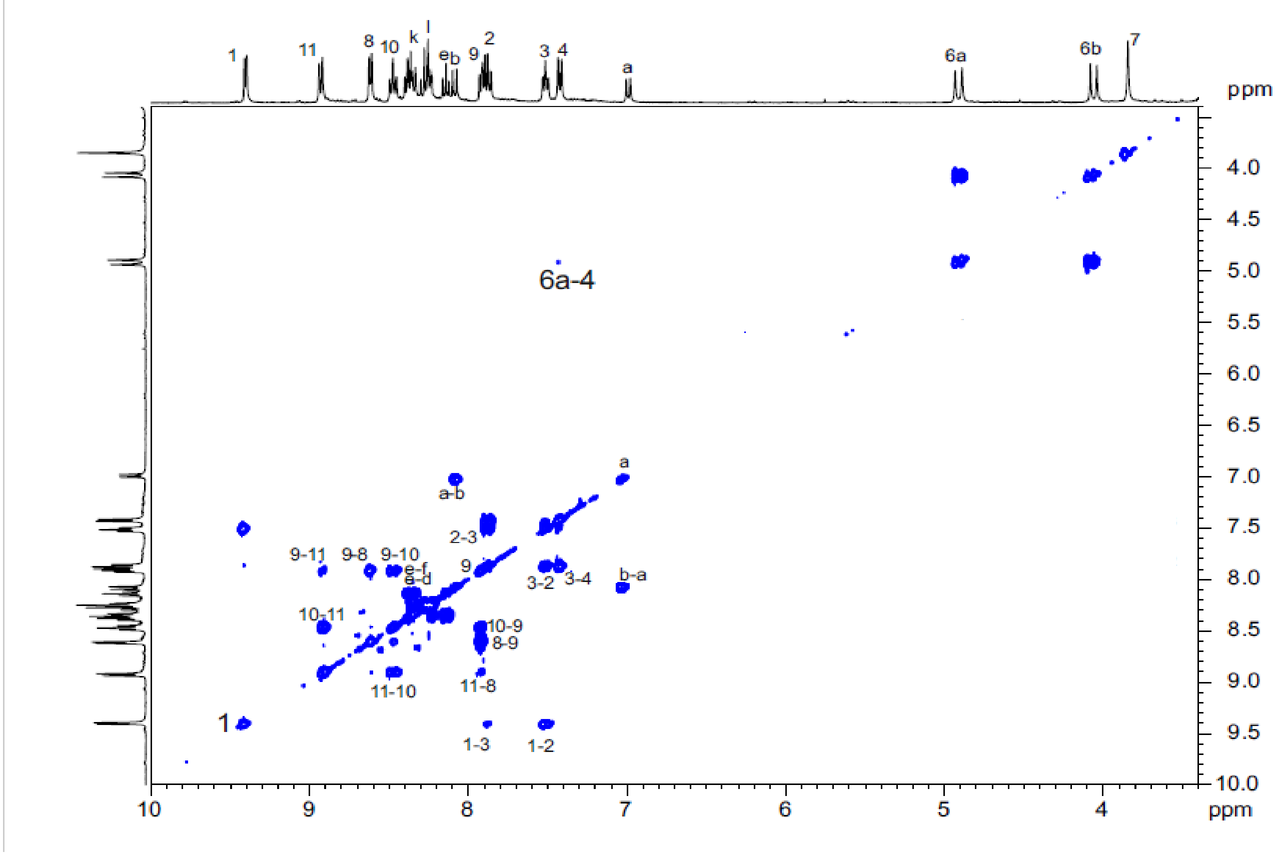

c)

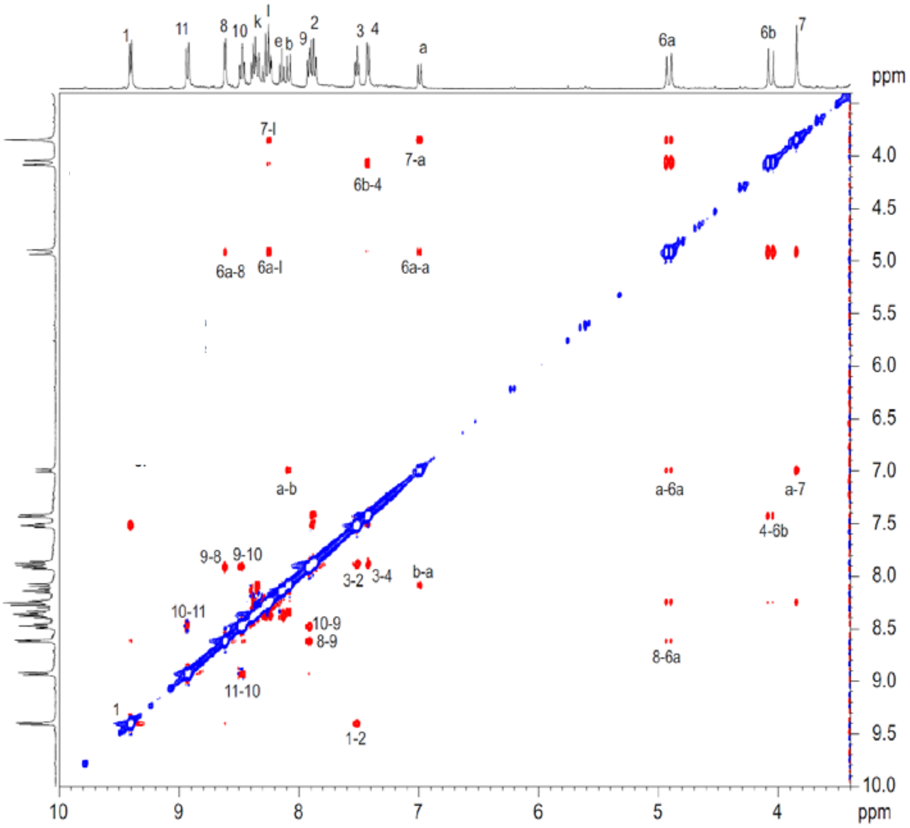
1
11
8
10
k
l
e
b
9
2
3
4
a
6a
6b
7
ppm
7-l
7-a
6b-4
6a-8
6a-l
6a-a
a-b
a-6a
a-7
4-6b
9-8
9-10
3-2
3-4
b-a
10-11
10-9
8-9
8-6a
11-10
1-2
4.0
4.5
5.0
5.5
6.0
6.5
7.0
7.5
8.0
8.5
9.0
9.5
10.0
ppm
10
9
8
7
6
5
4
ppm

d)

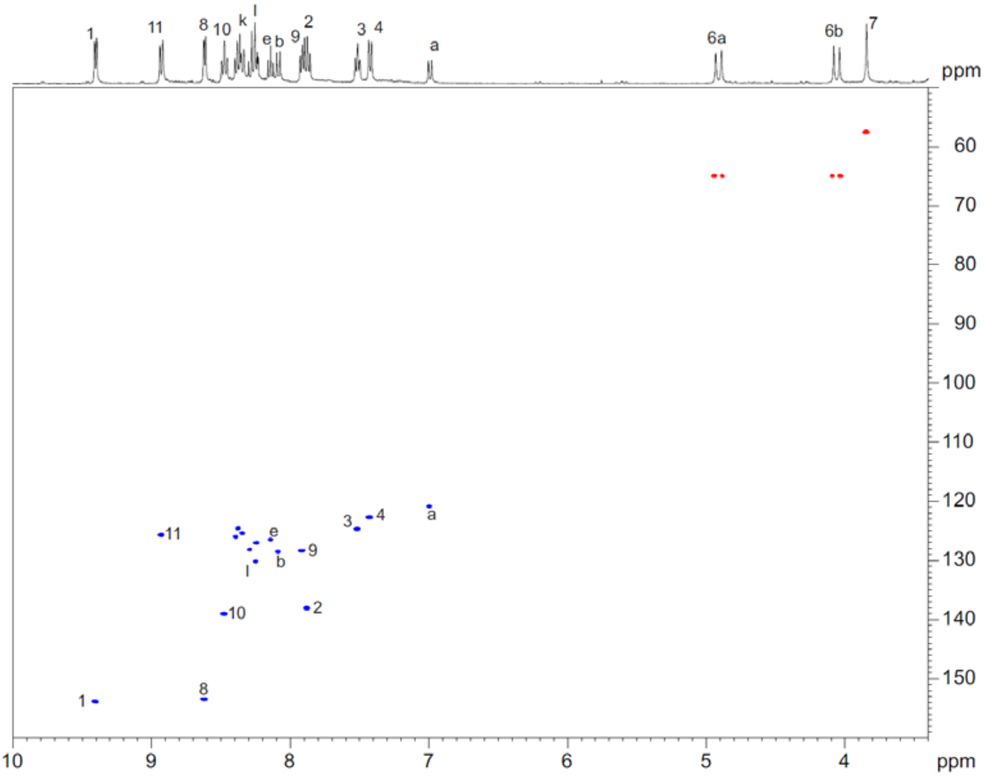
1
11
8
10
k
l
e
b
9
2
3
4
a
6a
6b
7
ppm
60
70
80
90
100
110
120
130
140
150
11
e
9
b
l
3
4
a
10
2
1
8
10
9
8
7
6
5
4
ppm

e)

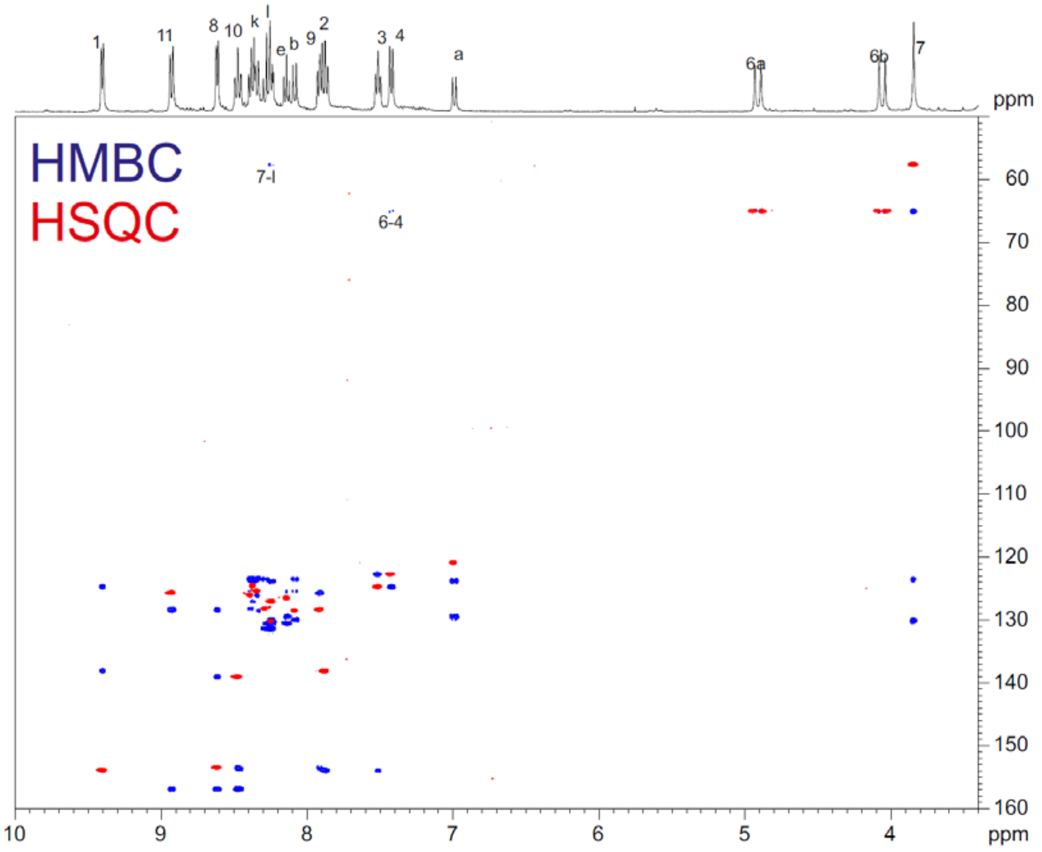
1
11
8
10
k
l
e
b
9
2
3
4
a
6a
6b
7
ppm
HMBC
HSQC
7-l
6-4
60
70
80
90
100
110
120
130
140
150
160
ppm
10
9
8
7
6
5
4
ppm

Figure S7. UV-*vis* spectra of *trans*-*fac*-**2** (blue line), *trans*-*fac*-**3** (orange line) in CH_2Cl_2.

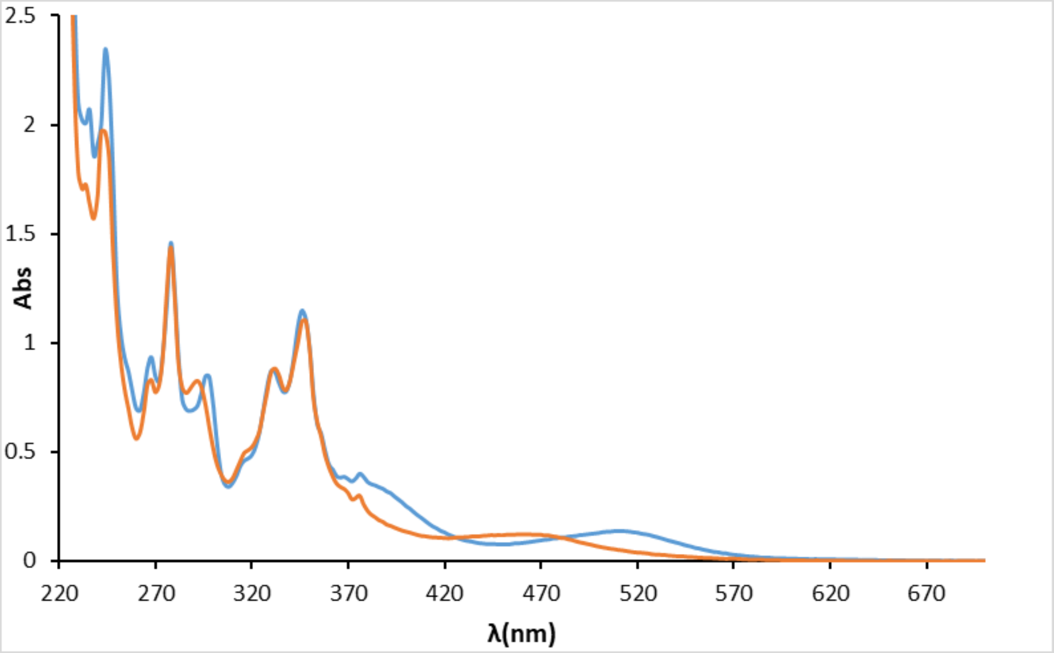

Figure S8. CV of a) ligand *bpea-pyrene* in CH_2Cl_2 containing 0.1 M [*n*-Bu_4N][PF_6] (TBAH) *vs* SCE.

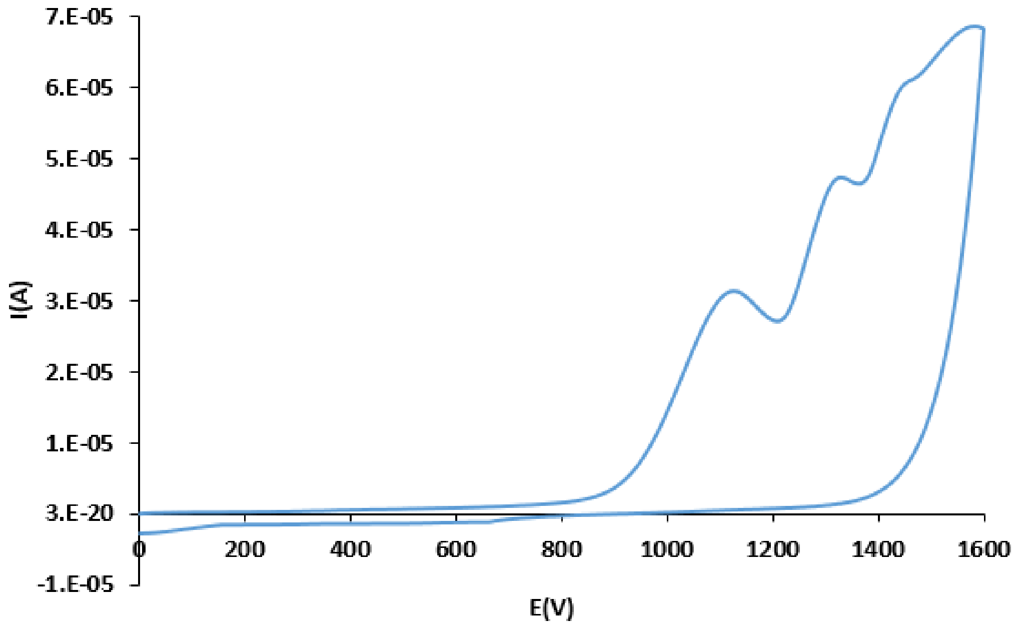

Figure S9. Differential Pulse Voltammetry (DPV) of a) *trans*-*fac*-**2** in CH_2Cl_2 containing 0.1 M [*n*Bu_4N][PF_6] (TBAH) *vs* SCE and, b) *trans*-*fac*-**3** in phosphate buffer (pH=6.8) *vs* SCE.

a)

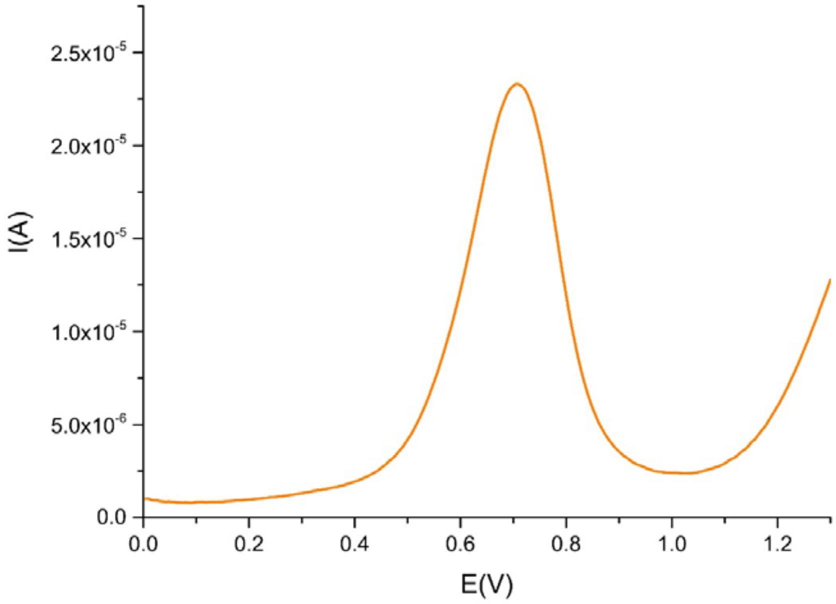

b)

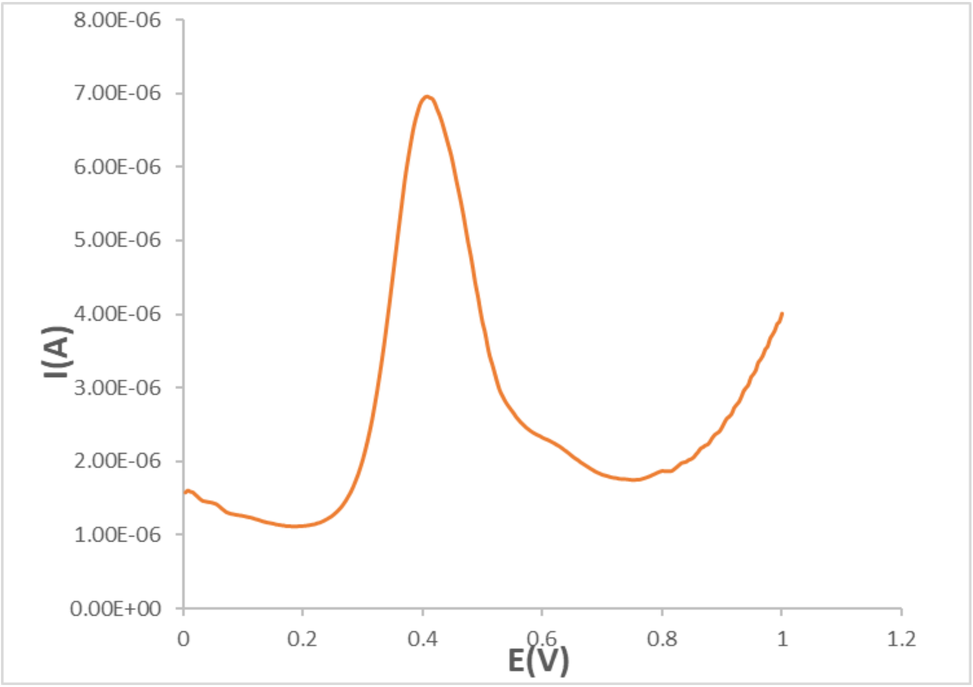

Figure S10. Pourbaix diagram for *trans-fac*-**3** in the range pH=2-9.

The Figure shows the Pourbaix diagrams for complex *trans-fac*-**3**, with the stability of the different ruthenium species as a function of the pH in the range pH 2-9. One pH-dependent redox processes throughout the whole pH range with a slope value of approximately 54 mV/pH unit are indicative of the occurrence of two-electron redox processes, Ru(IV/II).

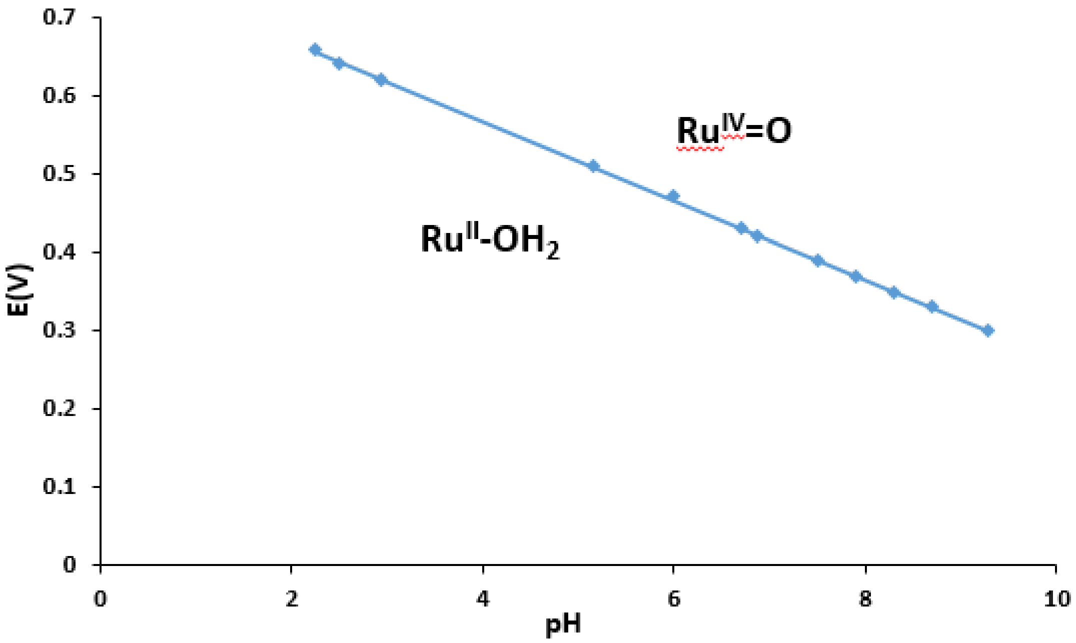

Figure S11. UV-*vis* spectrum of *trans*-*fac*-**3** **(**1mM) in water (blue line); UV-visible of the initial solution after 1 h in contact with GO (orange line); after 24 h (green line).

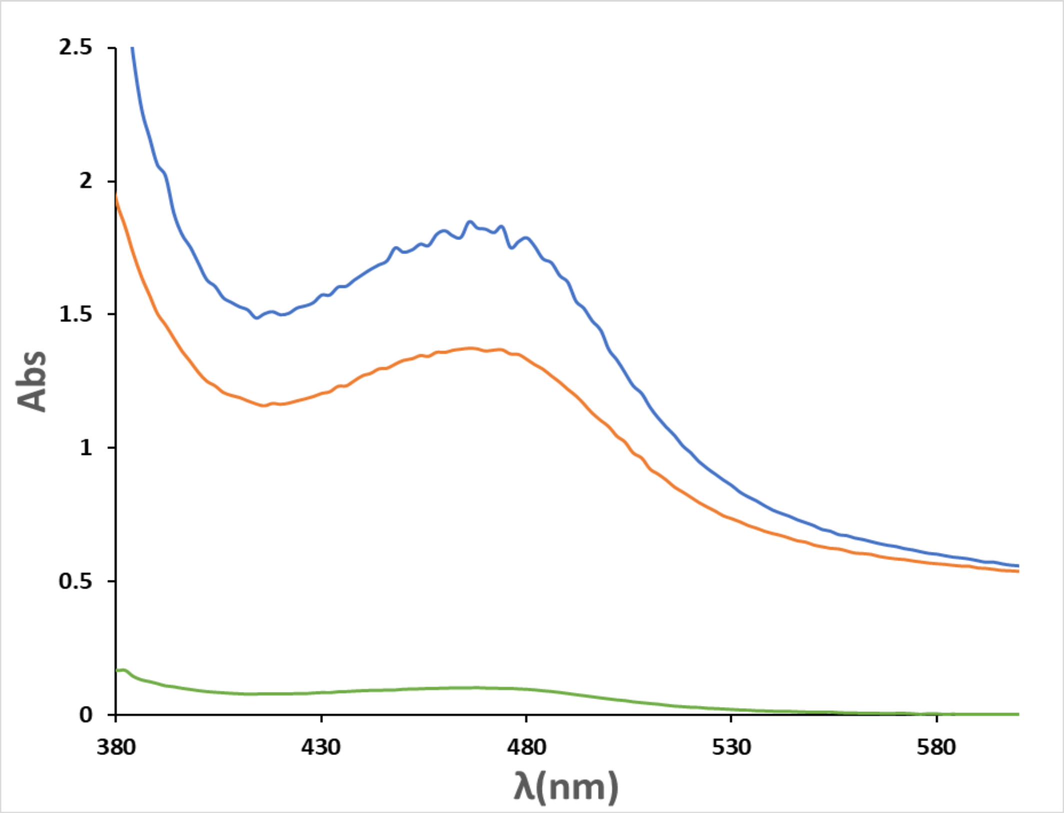

Figure S12. a) TEM image of GO@*trans*-*fac*-**3**; b) SEM images of GO support and b) GO@*trans*-*fac*-**3**.

a)

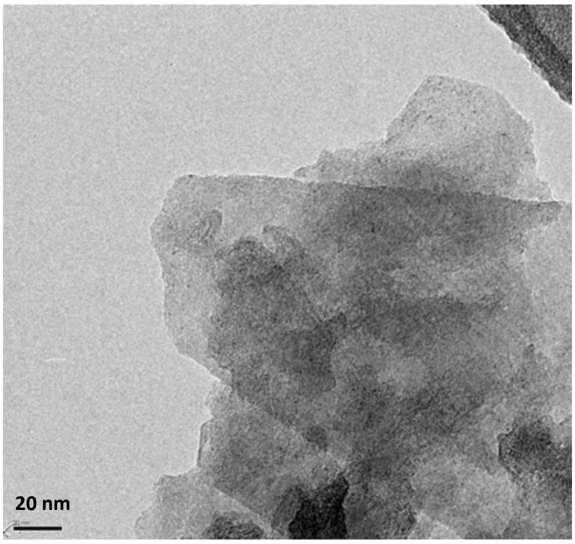

b)

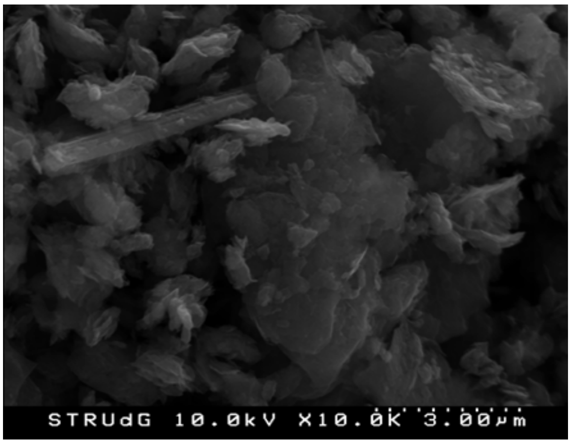

c)

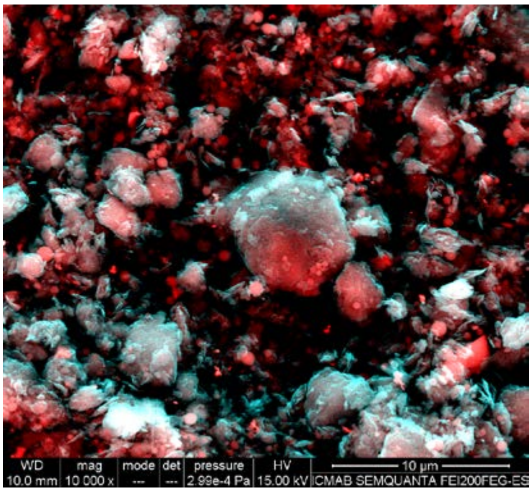

Figure S13. EDX spectra of GO@*trans-fac*-**3**.

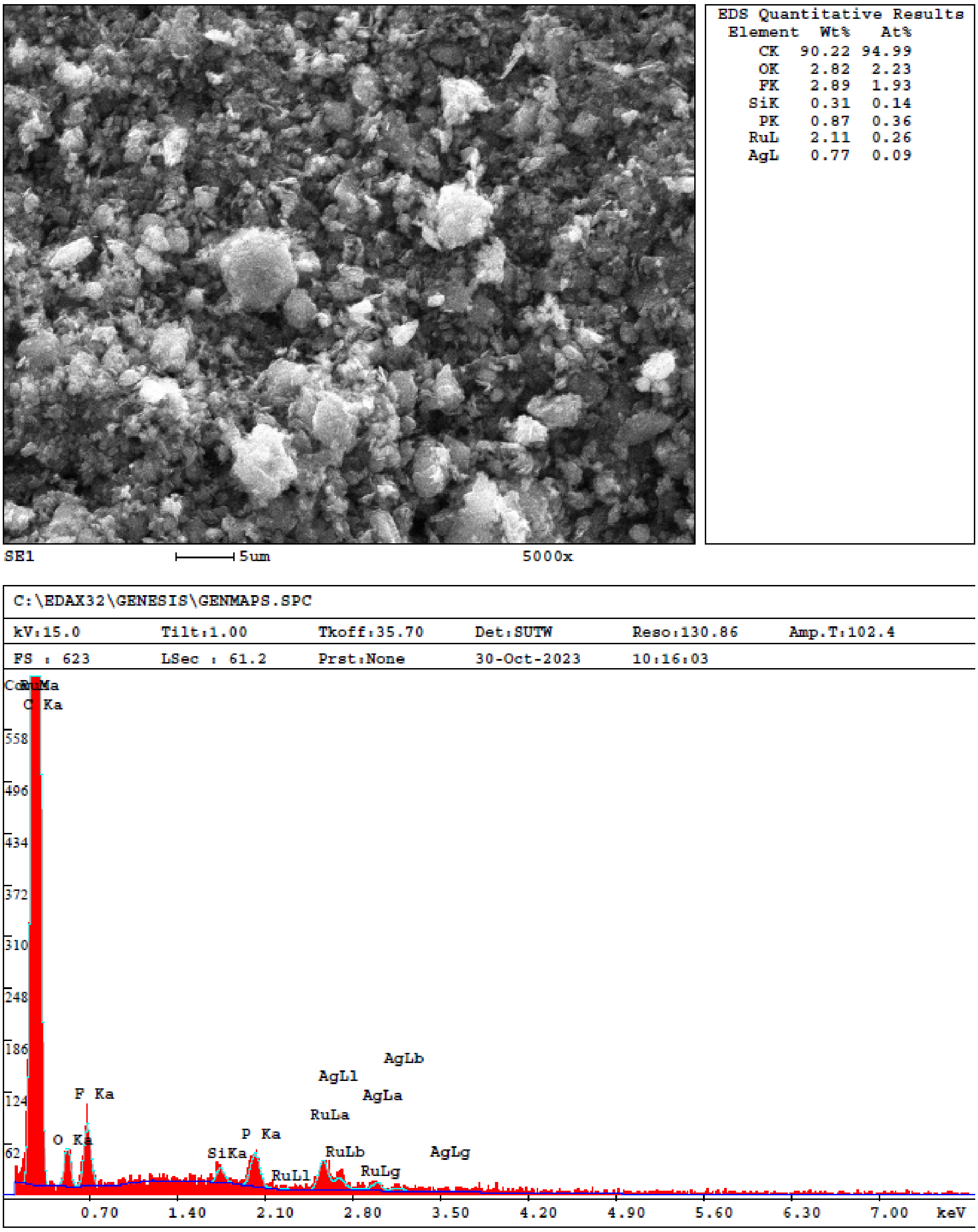

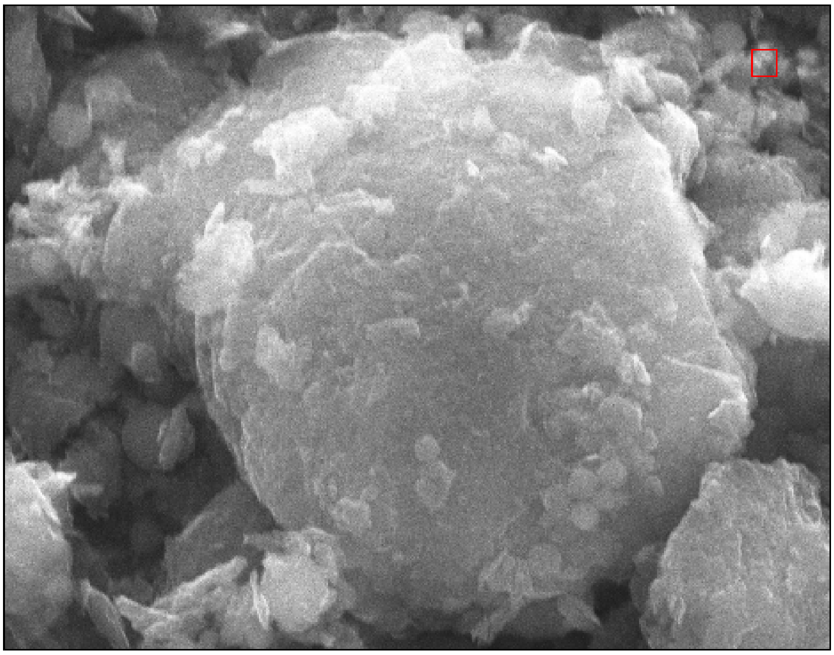

EDS Quantitative Results		
Element	Wt%	At%
CK	78.64	90.40
NK	3.06	3.02
OK	2.80	2.41
FK	2.95	2.15
SiK	0.43	0.21
PK	0.75	0.34
RuL	2.03	0.28
AgL	9.34	1.20

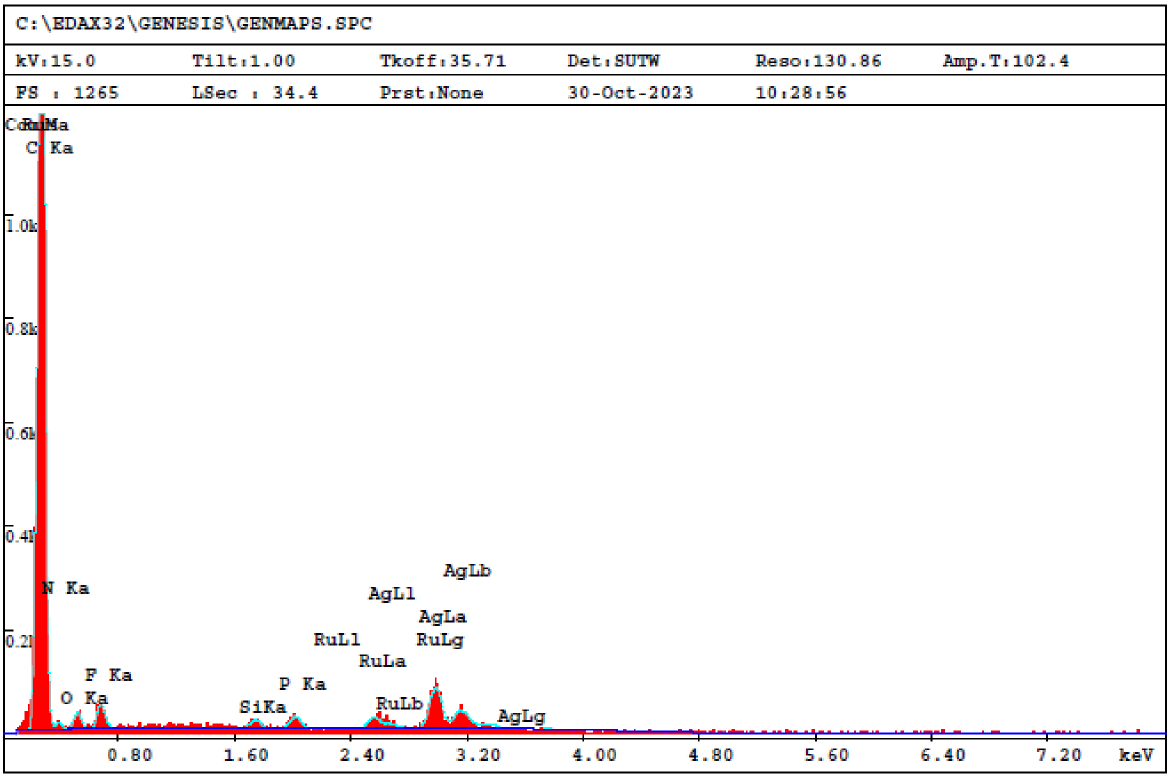

Figure S14. a) XPS spectra of GO@*trans*-*fac*-**3**; Magnification of the XPS spectrum of GO@*trans*-*fac*-**3** in b) the O1s region and c) the N1s region.

a)

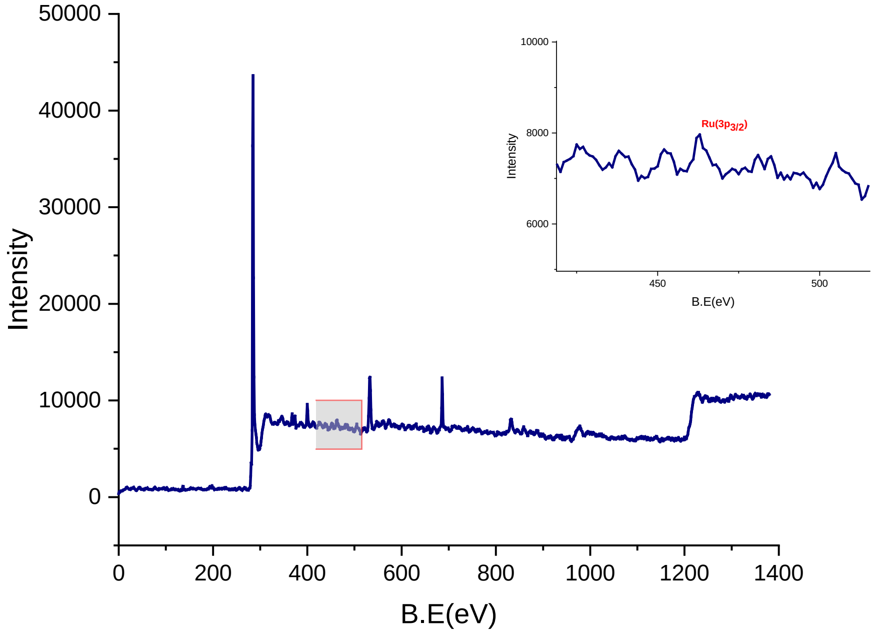

b)

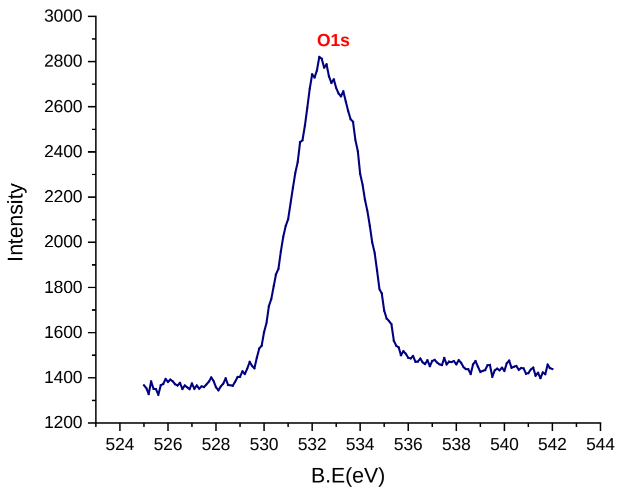

c)

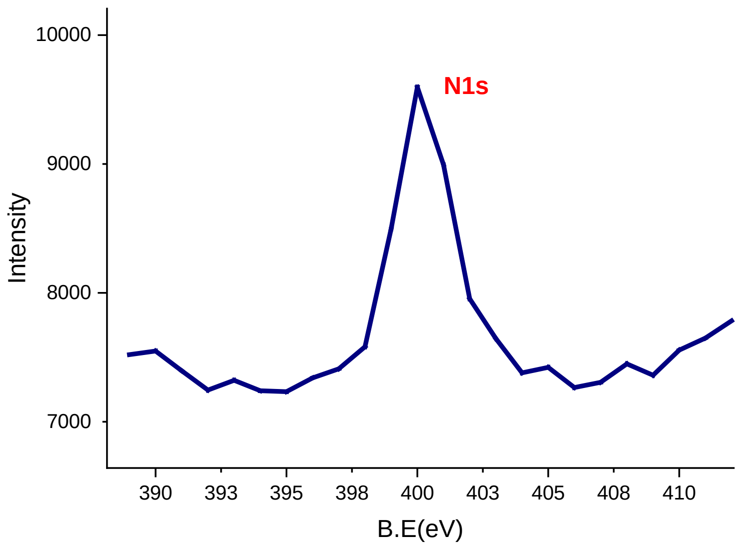

N1s
Intensity
10000
9000
8000
7000
390
393
395
398
400
403
405
408
410
B.E(eV)

Figure S15. UV-*vis* spectra of *trans*-*fac*-**3** (blue line) (0.1mM), GO@*trans*-*fac*-**3** (orange line) and GO (green line) in CH_2Cl_2.

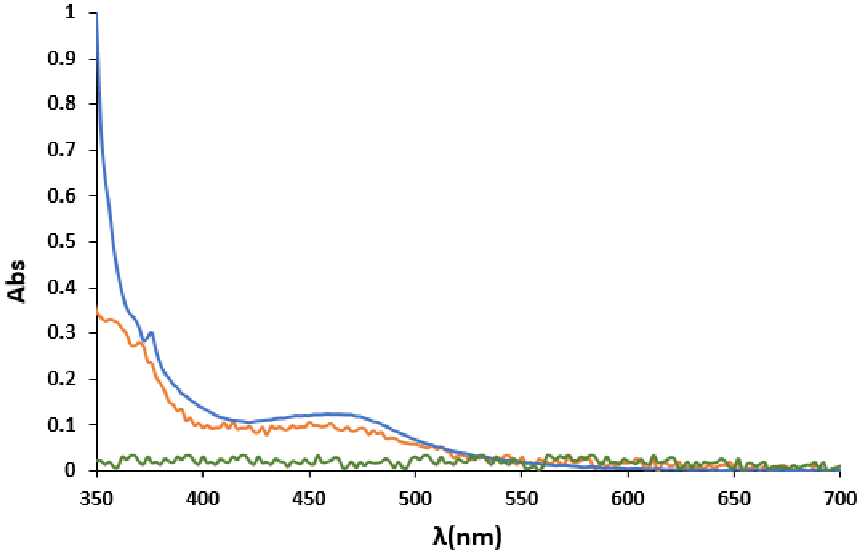

Figure S16. a) CV for the electropolymerization of *trans*-*fac*-**2** on GC electrode and b) CV of the polymerized GC/poly-*trans*-*fac*-**2** modified electrode in a blank solution, CH_2Cl_2 + 0.1 M TBAH.

a)

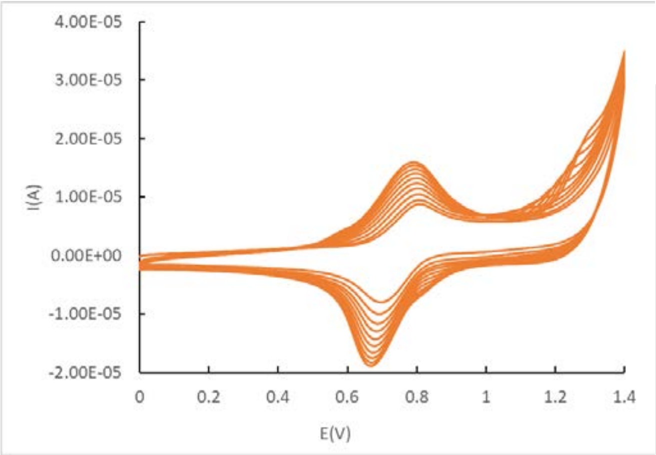

b)

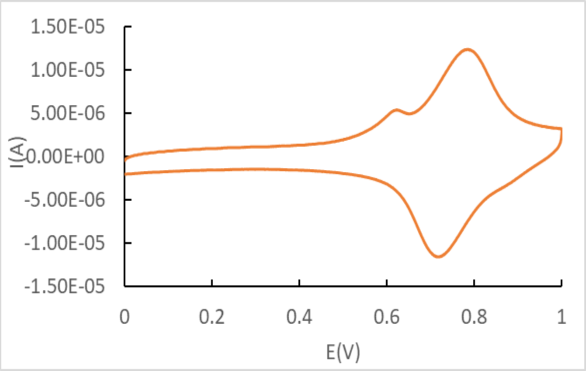

Figure S17. a) CVs of GC electrodes functionalized with 1mM of *trans-fac*-**2** in CH_2Cl_2+0.1MTBAH; b) Plot of cathodic and anodic peak current as function of the scan rate for GC/*trans-fac*-**2**.

a)

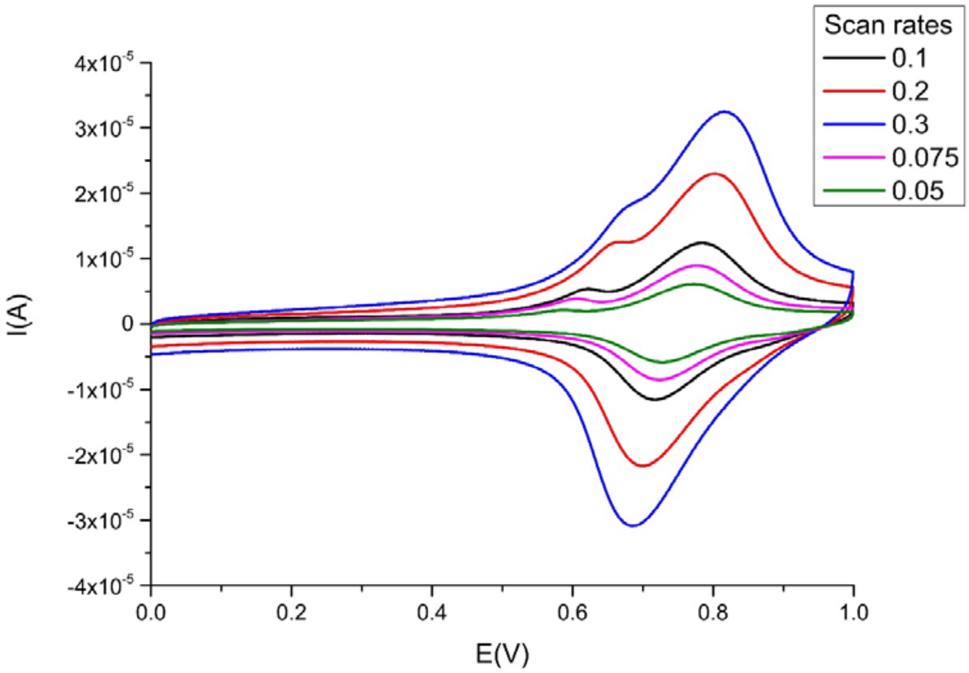

b)

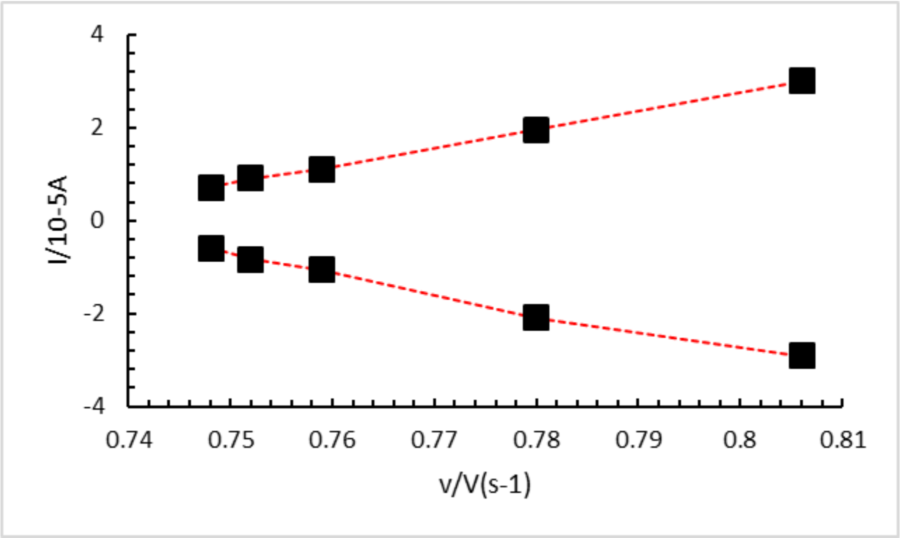

Figure S18. CV of the polymerized GC/poly-*trans*-*fac*-**3** modified electrode in a blank solution, CH_2Cl_2 + 0.1 M in TBAH.

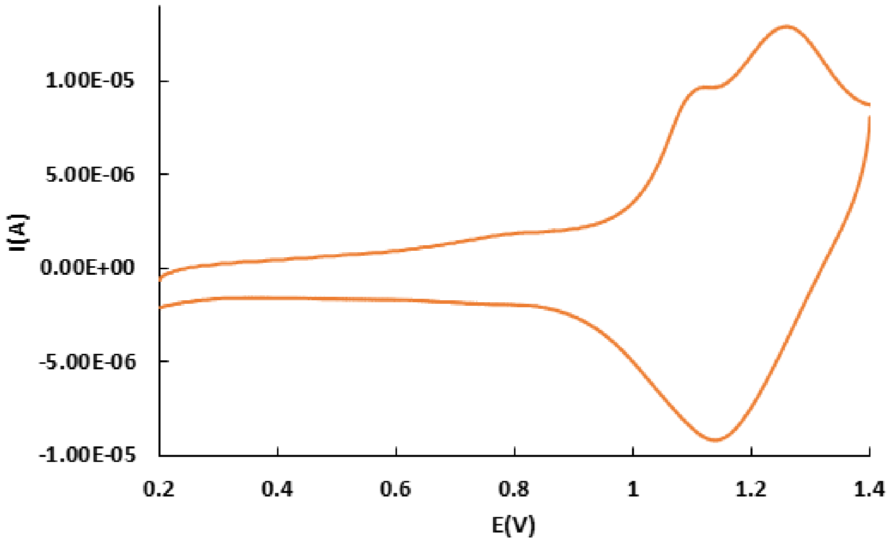

Figure S19. a) CV for the electropolymerization of *trans*-*fac*-**2** on graphite rods.

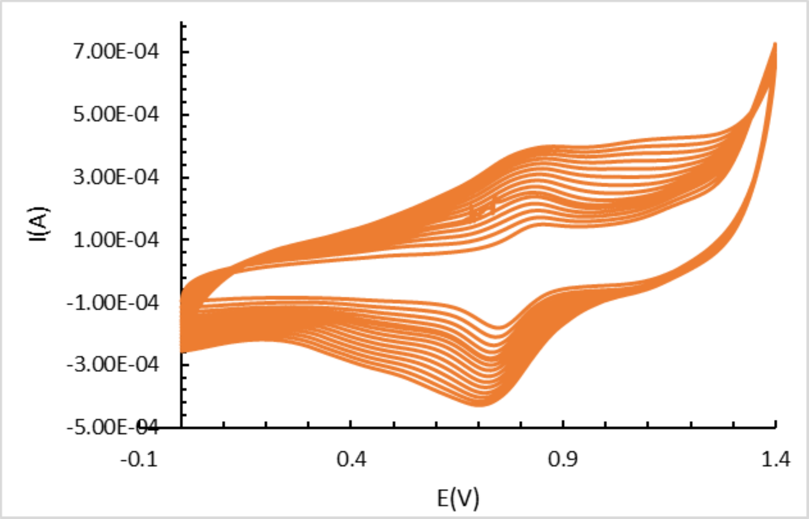

Figure S20. SEM images of GR/poly-*trans*-*fac*-**3**, a) using a SE detector and b) using a BSE detector.

a) **b)**

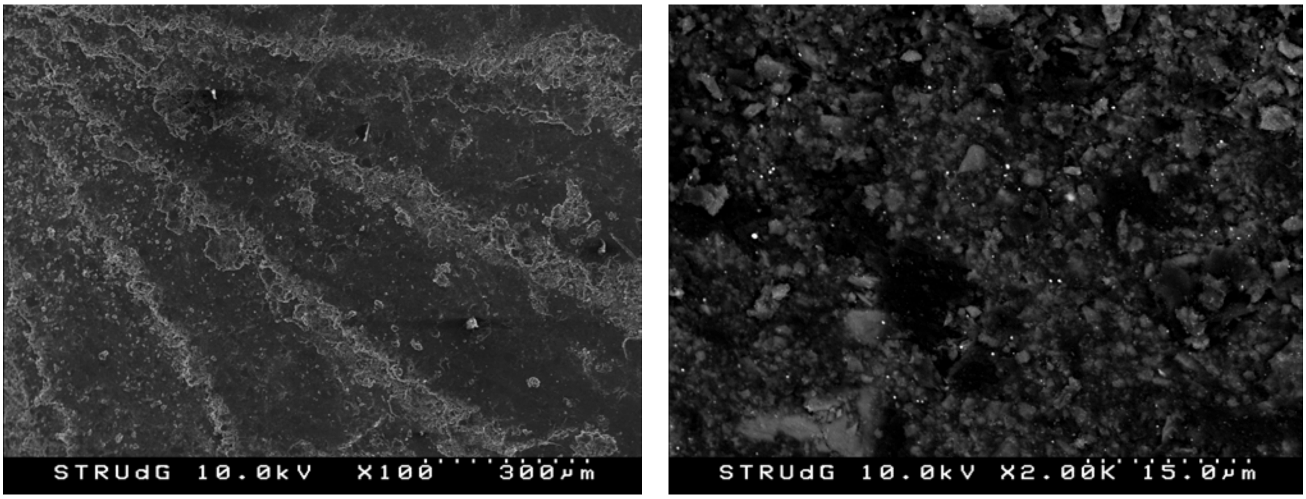

Figure S21. Plot of yield as a function of time for the photoredox catalysis of 1-phenylethanol using *trans*-*fac*-**3** as photocatalyst. Conditions: *trans*-*fac*-**3** (0,49 mM), substrate (49 mM), $Na_2S_2O_8$ (98 mM), 2.5 ml water (K$_2$CO$_3$, pH=7), light irradiation using a lamp with λ= 400-700 nm.

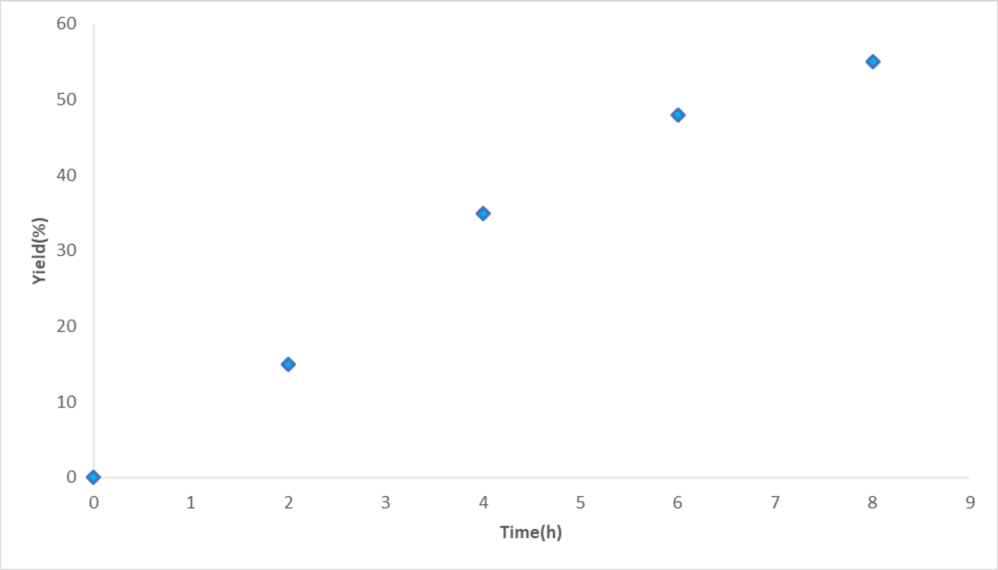

Figure S22. TEM images of GO@*trans*-*fac*-**3** a) before the photooxidation of 4-methylbenzyl alcohol and b) after five reuses.

a)

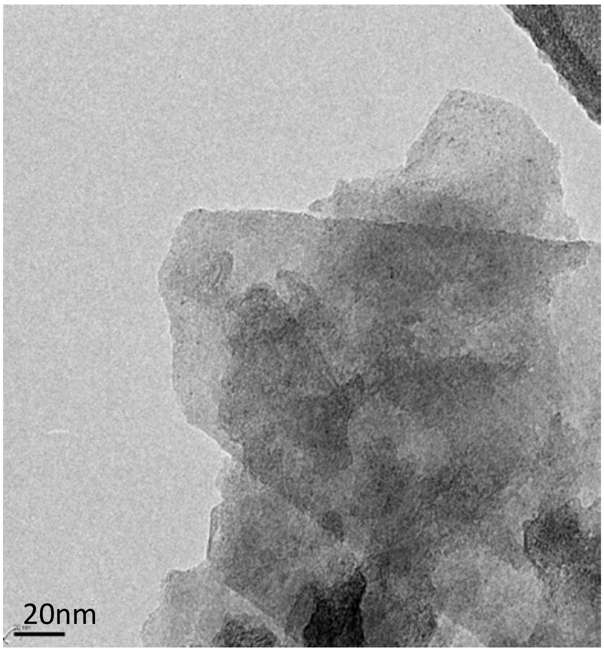

b)

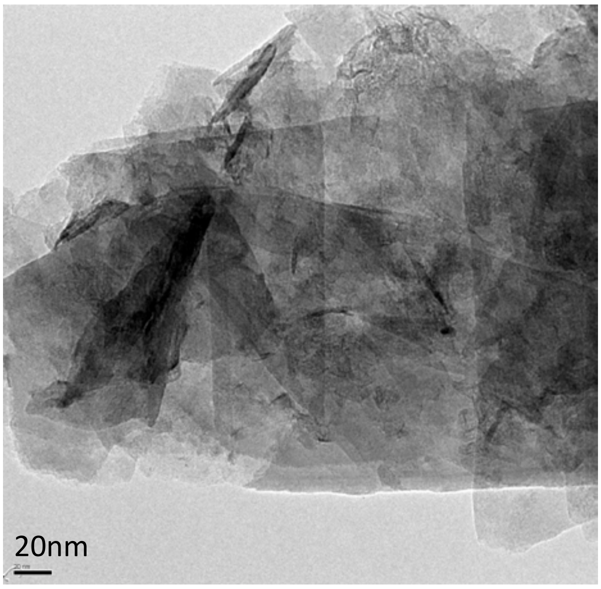

Figure S23. SEM images of GO@*trans*-*fac*-**3** in the photooxidation of 4-methylbenzyl alcohol after five reuses.

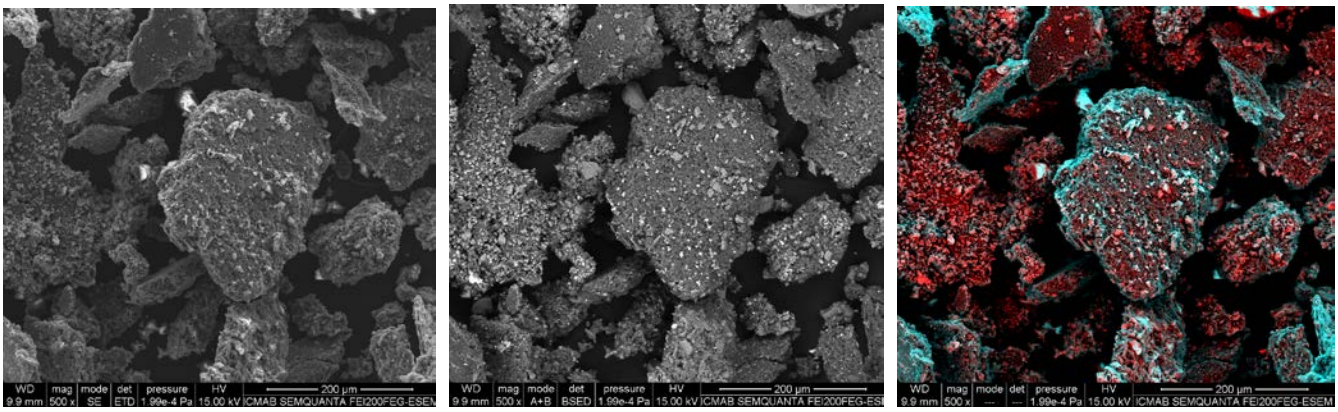

Supplement: Supplementary file 1 — am3c13156_si_001.pdf [file am3c13156_si_001.pdf]
